# Single-Chain Conformational Fluctuation Regulates Phase Separation of Intrinsically Disordered Proteins

**DOI:** 10.1021/jacsau.6c01044

**Published:** 2026-09-24

**Authors:** Cibo Feng, Shiqinrui Xu, Haibin Su, Xiakun Chu

**Affiliations:** † Advanced Materials Thrust, Function Hub, 567841 The Hong Kong University of Science and Technology (Guangzhou), Guangzhou 511453, China; ‡ Department of Chemistry, The Hong Kong University of Science and Technology, Clear Water Bay, Kowloon, Hong Kong SAR 999077, China; § Guangzhou Municipal Key Laboratory of Materials Informatics, The Hong Kong University of Science and Technology (Guangzhou), Guangzhou 511453, China; ⊥ Division of Life Science, The Hong Kong University of Science and Technology, Clear Water Bay, Kowloon, Hong Kong SAR 999077, China

**Keywords:** molecular dynamics simulation, coarse-grained model, intrinsically disordered protein, liquid−liquid phase separation, conformational fluctuation.

## Abstract

Intrinsically disordered proteins (IDPs) form biomolecular condensates through weak multivalent interactions, but the ensemble variables that control condensate stability and material properties remain incompletely defined. Most current descriptions emphasize sequence features or mean single-chain descriptors, such as the radius of gyration *R*_g_. Here, we ask whether the breadth of the single-chain ensemble can regulate liquid–liquid phase separation when the amino-acid sequence and mean chain size are fixed. We develop an all-atom-informed maximum-entropy coarse-grained model of the low-complexity domain of Fused in sarcoma (FUS-LCD) that preserves atomistic contact propensities while tuning the width of the isolated-chain *R*_g_ distribution. This design yields dynamically quenched (Qch), middle (Mid), and flexible (Flx) models with nearly identical ⟨*R*_g_⟩ but increasing conformational fluctuation. Increasing fluctuation produces chains that are locally compact yet globally extensible, and decouples condensate density from thermodynamic stability: Flx forms a dense low-temperature phase but has lower critical temperature and surface tension. Flx condensates also show faster diffusion, shorter contact lifetimes, accelerated stress relaxation, delayed early nucleation, and faster late-stage coalescence. This more dynamic state also exhibits weaker orientational organization, with Flx showing the lowest orientational order among the three models. Our results identify single-chain conformational fluctuation as an ensemble-level control variable that links local IDP organization to condensate thermodynamics, transport, viscoelasticity, assembly, and mesoscopic order.

## Graphical abstract

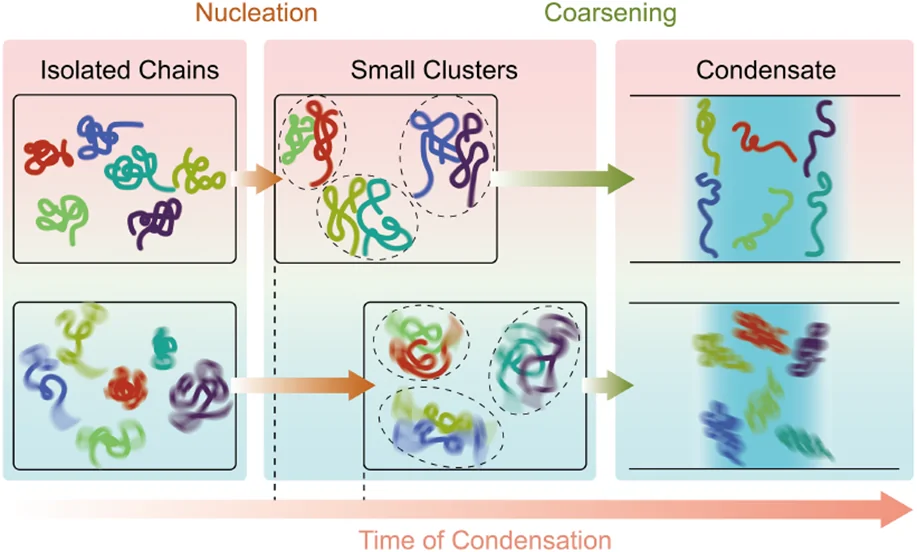

## Introduction

Liquid–liquid phase separation (LLPS) provides a physical mechanism by which cells concentrate selected proteins and nucleic acids into membraneless organelles. Rather than being enclosed by lipid membranes, these compartments arise through partitioning into dense, liquid-like phases that coexist with the surrounding dilute phase.^1–4^ Biomolecular condensates participate in RNA metabolism, transcriptional regulation, stress responses, DNA repair, and signaling, whereas aberrant changes in their composition or material state are associated with aging and neurodegenerative disease.^3,5–9^ These diverse functions raise a central physical question: how do condensates remain sufficiently stable to compartmentalize biomolecules while remaining sufficiently dynamic to support exchange, remodeling, and biochemical regulation?

Condensates are not passive droplets of concentrated molecules. They are dynamic polymeric and supramolecular networks whose phase boundaries, molecular exchange, surface tension, and viscoelastic response are governed by weak multivalent interactions, chain conformational entropy, and network connectivity.^10–14^ Measurements on LAF-1 droplets, for example, showed that disordered-protein condensates can exhibit tunable viscosity and molecular dynamics.^15^ Thus, the key challenge has shifted from identifying condensates as liquid-like assemblies to determining the molecular variables that encode their thermodynamic stability, internal organization, and material properties.

Intrinsically disordered proteins (IDPs) and intrinsically disordered regions (IDRs) provide a natural molecular basis for such regulation. Unlike folded proteins, IDPs do not adopt a single stable three-dimensional structure. Instead, they populate heterogeneous ensembles of interconverting conformations, so their biological behavior is often encoded statistically by the ensemble rather than structurally by one representative state.^16,17^ Conformational dynamics and conformational entropy can therefore actively regulate molecular recognition and condensate chemistry, rather than merely reflecting a lack of structure.^18,19^ In the sticker–spacer framework, distributed stickers, including aromatic, charged, polar, or short-motif residues, mediate transient intra- and intermolecular contacts, whereas spacers tune chain dimensions, flexibility, and the entropic cost of bringing stickers together.^12,20,21^ As a result, the same sequence features that shape the dilute single-chain ensemble also influence collective phase behavior. A fundamental question then follows: which properties of the single-chain ensemble are predictive of many-chain LLPS?

Substantial progress has been made in connecting single-chain properties to phase separation. Coarse-grained (CG) simulations and analytical theories have shown that sequence composition, charge patterning, and residue-specific interaction strengths can regulate both the radius of gyration, *R*_g_, of an isolated chain and the coexistence curve of the corresponding condensed phase.^22–25^ Models optimized against single-chain observables have further improved predictions of IDP phase diagrams, supporting the idea that dilute-phase conformational information provides a useful proxy for the interactions that operate inside condensates.^26–28^ Related design studies have shown that IDP variants can be engineered to display distinct compaction, long-range contact patterns, and phase propensities, further reinforcing a sequence–ensemble–phase relationship.^29^ These studies established an important principle: predicting IDP condensates requires not only quantifying how strongly chains attract one another, but also determining what conformational states the chains prefer before entering the dense phase.

A limitation of this view is that single-chain effects are often discussed in terms of average quantities, especially the mean radius of gyration, ⟨*R*_g_⟩.^24^ This focus is reasonable because ⟨*R*_g_⟩ is experimentally accessible and often correlates with phase propensity. Yet an IDP ensemble is a distribution, not a single number.^30^ Two ensembles can have nearly identical mean dimensions while differing in the width of their size distributions, the heterogeneity of intrachain distances, the anisotropy of conformations, or the time scale of structural reorganization.^22,31^ Such differences are likely to matter for LLPS because condensate formation requires chains to deform, reorganize contacts, exchange binding partners, relax stresses, and reshape interfaces across multiple length and time scales. Large-scale IDR simulations indicate that human disordered regions span diverse ensemble classes;^17^ charge patterning and residue-specific interactions can reshape intrachain contacts in ways that are not reducible to chain length or mean size;^21,22^ and theoretical analyses of small-angle X-ray scattering form factors show that conformationally distinct heterogeneous ensembles can exhibit similar apparent global dimensions.^32^ Together with prior work emphasizing the potential importance of conformational fluctuations in liquid-like protein assemblies,^33^ these observations motivate the central question of this study: can the breadth of the dilute single-chain ensemble regulate LLPS even when the amino-acid sequence and mean chain size are held fixed?

We address this question using Fused in sarcoma (FUS) as a model system. FUS is an RNA-binding protein involved in transcription, RNA processing, stress granule formation, and neurodegenerative disease. Its N-terminal low-complexity domain (FUS-LCD) forms hydrogels and liquid-like assemblies.^34–38^ Residue-level NMR, spectroscopy, mutagenesis, and simulations have shown that FUS-LCD remains largely disordered in the condensed phase and is stabilized by many weak, transient, and distributed contacts rather than by a unique folded structure.^20,21,39–43^ These properties make FUS-LCD well suited for testing whether ensemble fluctuation, rather than only mean compaction or sequence composition, can tune condensate behavior.

To isolate this variable, we construct a residue-level CG model of FUS-LCD using all-atom (AA) simulation data and a maximum-entropy (ME) framework. The ME strategy allows the CG model to reproduce AA-derived contact propensities while minimally perturbing the prior ensemble.^44,45^ By tuning the width of the *R*_g_ distribution while maintaining nearly identical ⟨*R*_g_⟩, we generate three FUS-LCD models with the same amino-acid sequence but different levels of conformational fluctuation. This design separates the effect of ensemble breadth from changes in sequence composition and average chain size. We then compare their single-chain structure and dynamics, dense-phase thermodynamics, orientational organization, contact-network topology, diffusion, viscoelastic relaxation, and condensation kinetics. We find that increasing conformational fluctuation produces condensates that are denser at low temperature yet less thermodynamically stable, with lower critical temperature and surface tension. At the same time, these condensates become more dynamic, showing faster diffusion, shorter contact lifetimes, accelerated stress relaxation, delayed early nucleation, and faster late-stage coalescence. This more liquid-like dynamical state is also accompanied by reduced orientational organization, indicating that enhanced conformational fluctuation weakens both persistent intermolecular connectivity and collective chain alignment. These results identify conformational fluctuation as an ensemble-level variable that couples microscopic IDP flexibility to macroscopic condensate stability, transport, viscoelasticity, kinetics, and mesoscopic organization.

## Results

### An AA-Informed ME-CG Model Isolates Single-Chain Conformational Fluctuation

To determine whether the breadth of an IDP conformational ensemble can regulate LLPS independently of its mean chain size, we first developed a residue-level CG model parametrized by AA simulations. Each FUS-LCD residue was represented by one bead, adjacent beads were connected by harmonic bonds, and screened electrostatic interactions were assigned according to residue charge. The remaining short-range nonbonded interaction was decomposed into an excluded-volume term and a data-driven attractive term. The residue-type attractive coefficients were optimized using an ME framework so that the CG model reproduced AA-derived intrachain contact propensities while minimally perturbing the prior ensemble.^46–49^ This strategy preserves residue-level interaction information from the AA reference while retaining the computational efficiency required for many-chain phase-separation simulations.

We then used this framework to construct three FUS-LCD models that differ primarily in the width of the isolated-chain *R*_g_ distribution while maintaining nearly identical ⟨*R*_g_⟩. The three models are denoted as dynamically quenched (Qch), middle (Mid), and flexible (Flx), corresponding to low, intermediate, and high conformational fluctuation, respectively (Figure 1A–C). Their *R*_g_ distributions bracket the AA reference in width, whereas their mean *R*_g_ values remain matched by construction (Figure 1D,E). Because the amino-acid sequence is identical in all three models, this design separates ensemble breadth from sequence composition, chain length, and average chain compaction. The optimized residue-type interaction coefficients differ only modestly among Qch, Mid, and Flx (Figure S1A–C), indicating that tuning conformational fluctuation does not require a wholesale rewiring of the underlying residue-level interaction pattern. The interaction between Asp and Met is an exception. They repel each other in Mid and Flx but interact weakly in Qch (Figure S1D–F). Consistently, the CG contact maps reproduce the AA-derived contact propensities, particularly at the residue-type level (Figures S2–S4).

**Figure 1:**
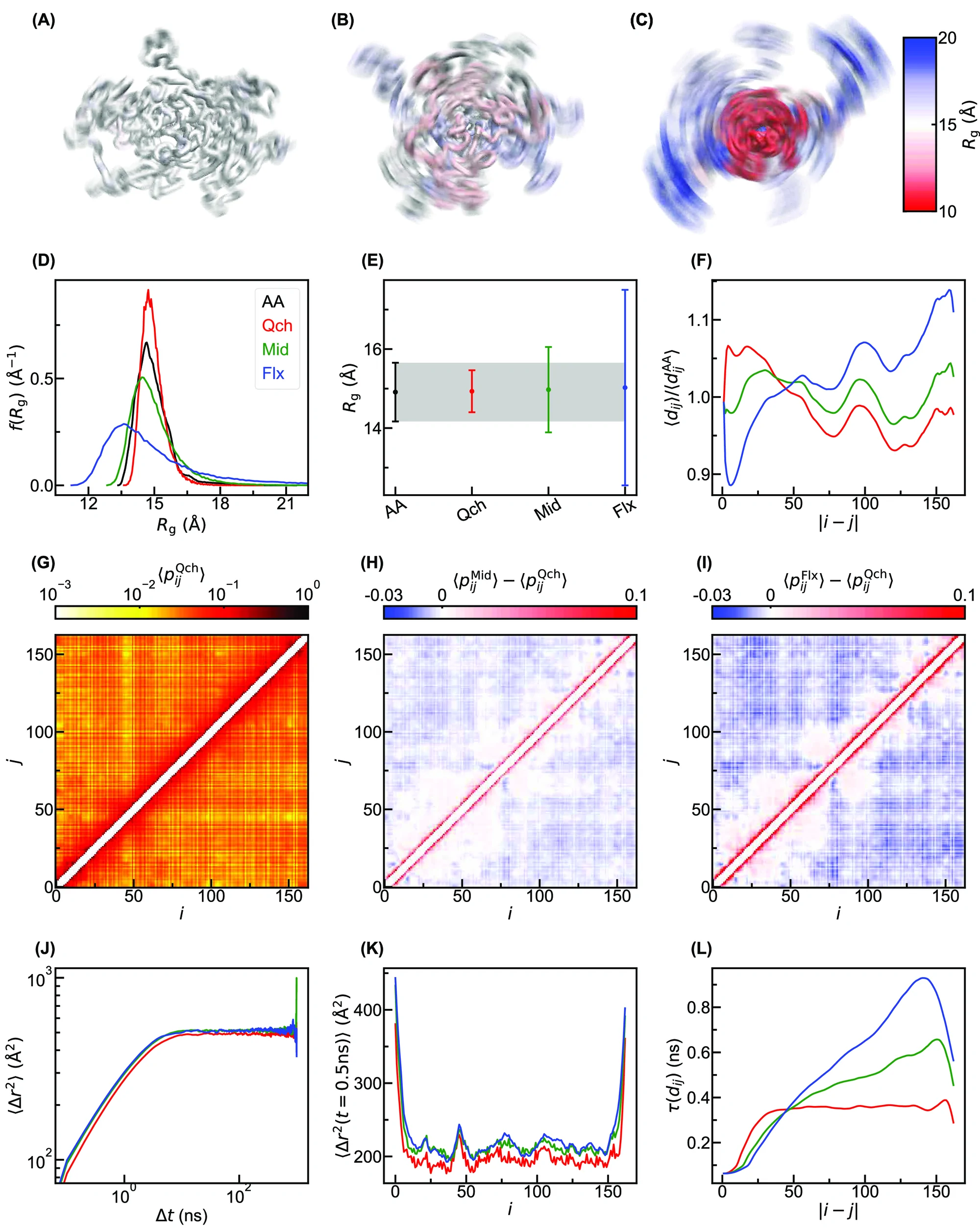
Single-chain structural and dynamical properties of FUS-LCD at 300 K in the AA reference and three CG models. (A–C) Representative conformational ensembles of the low-fluctuation dynamically quenched (Qch) model, intermediate-fluctuation middle (Mid) model, and high-fluctuation flexible (Flx) model. Conformations are colored by radius of gyration *R*_g_, with red indicating smaller *R*_g_ and blue indicating larger *R*_g_. (D) Probability distributions of *R*_g_ for the AA, Qch, Mid, and Flx ensembles. (E) Mean and standard deviation of *R*_g_; the gray band denotes the AA mean ± standard deviation. (F) Ratio of the mean intrachain residue-pair distance ⟨*d*_*ij*_⟩ in each CG model to the AA value as a function of sequence separation |*i*–*j*|. (G) Pairwise inter-residue contact map of Qch. (H–I) Contact-map differences for Mid versus Qch and Flx versus Qch. (J) Residue-averaged mean-squared displacement (MSD), ⟨Δ*r* ^2^⟩, calculated relative to the chain center of mass. (K) Residue-resolved MSD at Δ*t* = 0.5 ns. (L) Relaxation time τ­(*d*_*ij*_) of residue-pair distance autocorrelation as a function of sequence separation.

We next asked whether the three models differ only in the width of *R*_g_, or whether broader conformational changes accompany this tuning. Principal component analysis (PCA) of the isolated-chain ensembles provides a compact representation of these differences (Figure S5A–G). In the two-dimensional space spanned by the first two PCA coordinates, ζ_1_ and ζ_2_, the distributions broaden systematically from Qch to Mid to Flx, whereas their mean positions remain nearly unchanged and close to the AA reference. Thus, increasing fluctuation expands the accessible conformational space without substantially shifting the average conformation. By contrast, the hydropathy-scale Urry (HPS-Urry) model^28^ shows broader distributions together with a clear shift in the mean value of ζ_1_ (Figure S5H,I), indicating that it changes both ensemble breadth and average geometry.

To interpret the PCA coordinates physically, we compared them with global shape descriptors, including the three principal radii *R*_1_, *R*_2_, and *R*_3_, the radius of gyration *R*_g_, the asphericity Δ, and the shape parameter *S* (Figures S6–S10). The first PCA coordinate, ζ_1_, is strongly correlated with *R*_1_ and *R*_g_, and also with the anisotropy descriptors Δ and *S* (Figure S5J–N). This correlation reflects the fact that FUS-LCD conformations are generally elongated rather than spherical, with *R*_1_ larger than *R*_2_ and *R*_3_. The anticorrelation between *R*_1_ and the two smaller principal radii further indicates that conformational extension along the dominant axis is partially compensated by contraction along the transverse axes. In contrast, ζ_2_ is only weakly correlated with these global geometric quantities, suggesting that it captures additional conformational variation beyond overall chain extension and anisotropy.

A residue-pair distance analysis reveals how these ensemble-level differences are encoded along the sequence. We calculated the distribution of intrachain distances *d*_*ij*_, together with their mean and standard deviation as functions of sequence separation |*i*–*j*| (Figure S11). Although Qch, Mid, and Flx have nearly identical ⟨*R*_g_⟩, their distance profiles differ in a length-scale-dependent manner. Relative to the AA reference, Flx exhibits shorter distances at small sequence separations but longer distances at large sequence separations, whereas Qch shows the opposite trend (Figure 1F). Thus, the high-fluctuation model combines local compaction with long-range extensibility. This redistribution of intrachain distances explains how ensembles with matched mean *R*_g_ can nevertheless possess distinct conformational heterogeneity and deformation capacity. Consistently, local contacts increase from Qch to Flx, whereas long-range contacts decrease (Figure 1G–I). This further demonstrates that local compaction and long-range extensibility can balance the overall chain size while modulating size fluctuations.

Finally, we characterized the internal dynamics of isolated chains. The residue mean-squared displacement (MSD), calculated relative to the chain center of mass, reports the amplitude of internal residue motion (Figure S12). The residue-averaged MSD increases modestly from Qch to Mid to Flx (Figure 1J). The residue-resolved MSD at Δ*t* = 0.5 ns shows that this trend is most apparent for internal residues, whereas terminal residues are highly mobile in all three models and are therefore less diagnostic of model-dependent flexibility (Figure 1K). We also quantified the relaxation time τ­(*d*_*ij*_) of residue-pair distances from the distance autocorrelation function *f*_*d*_*ij*__^ac^, using the lag time at which the autocorrelation decays to 1/e (Figure S13). The relaxation behavior is strongly dependent on sequence separation: Flx shows shorter relaxation times for short-range residue pairs but longer relaxation times for long-range residue pairs (Figure 1L). Thus, increasing conformational fluctuation does not simply make the chain uniformly faster or slower. Instead, it redistributes both structural heterogeneity and dynamical relaxation across sequence length scales. These matched-⟨*R*_g_⟩ but fluctuation-distinct models therefore provide a controlled platform for testing how single-chain ensemble breadth influences the thermodynamics, organization, dynamics, and kinetics of FUS-LCD condensates.

### Conformational Fluctuation Decouples Condensate Density from Thermodynamic Stability and Reorganizes Mesoscale Structure

To determine how single-chain conformational fluctuation affects dense-phase thermodynamics, we performed 100-chain slab simulations for Qch, Mid, and Flx.^23^ The elongated slab geometry produced two dense–dilute interfaces normal to the *z* direction, allowing density, interfacial structure, and chain conformations to be analyzed along a common spatial coordinate. Representative configurations show that, at the same temperature, the condensate interface becomes progressively more diffuse and irregular from Qch to Mid to Flx (Figure 2A–C). Density profiles further show that Flx has the highest dense-phase concentration at 300 K (Figure S14A–D). If dense-phase concentration were considered alone, this result might suggest stronger phase separation. However, the temperature dependence reveals the opposite trend in thermodynamic stability. Upon heating, the Flx slab is disrupted more readily than Qch and Mid, and a dilute-phase tail appears earlier under the same sampling conditions (Figures S15A–C, S16A–D and S17A–C). Consistently, coexistence-curve fitting gives the lowest critical temperature, *T*_c_, for Flx (Figure 2D). The surface tension, Γ, is also lowest for Flx and decreases most strongly with temperature (Figures 2E, S18 and S19). Thus, increasing conformational fluctuation produces a condensate that is locally concentrated at low temperature but less robust against thermal disruption and interfacial deformation.

**Figure 2:**
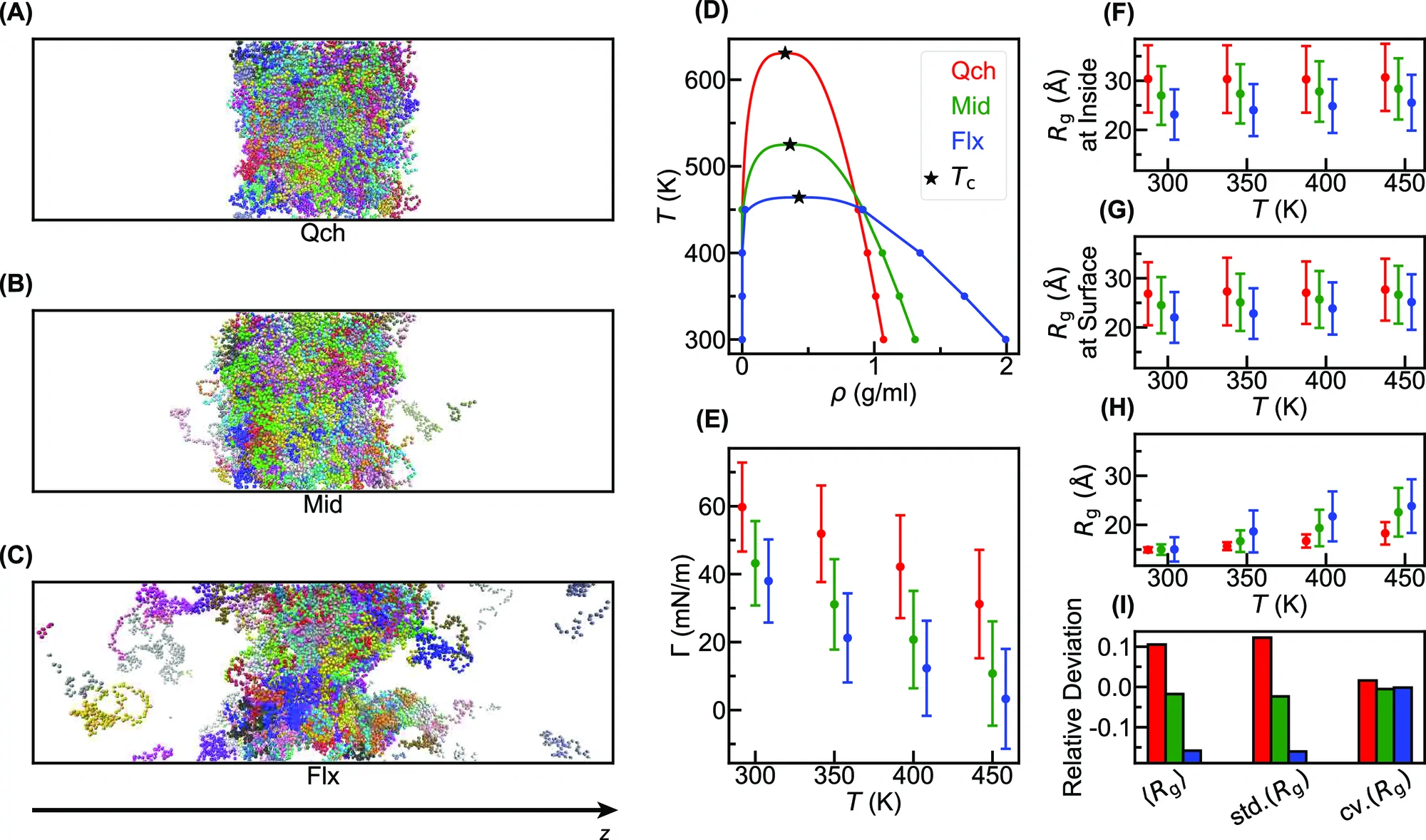
Thermodynamic stability and chain dimensions in 100-chain slab simulations of Qch, Mid, and Flx. (A–C) Representative slab configurations of Qch, Mid, and Flx. (D) Temperature–density coexistence curves obtained from slab density profiles; stars mark the fitted critical temperatures *T*_c_. (E) Surface tension Γ as a function of temperature; error bars denote standard deviation. (F) Radius of gyration *R*_g_ of chains located inside the condensed phase. (G) *R*_g_ of chains located at the slab surface. (H) *R*_g_ of isolated chains. (I) Relative deviations of ⟨*R*_g_⟩, std.(*R*_g_), and cv.(*R*_g_) inside the condensate. Each value is normalized by the average over all 12 conditions, corresponding to three models and four temperatures, allowing quantities with different units and numerical scales to be compared.

To further compare the thermodynamic behavior of the three models, we calculated the second virial coefficient *B*_22_ from double-chain simulations. Umbrella sampling was used to obtain the potential of mean force (PMF) as a function of the center-of-mass separation between the two chains. The *B*_22_ value derived from the PMF increases from Qch to Flx (Figure S20). This trend is consistent with previous studies in which a higher LLPS critical temperature was associated with a lower *B*_22_ value.^24^

We next asked how the matched isolated-chain *R*_g_ ensembles are remodeled inside the condensate. Because the finite slab is not thick enough to provide a completely bulk-like interior, chain conformations depend on the position of the chain center of mass along *z* (Figure S21). Moreover, the chain center-of-mass density is enriched near two surface-adjacent regions rather than being uniformly distributed across the slab interior (Figures S14A–L, S15A–I, S16A–L, and S17A–I). We therefore compared two representative environments: the condensate interior and the interfacial surface. In all three models, chains expand substantially upon entering the condensate relative to the isolated state (Figures 2F–H and S22A–T). However, the magnitude of this expansion depends strongly on conformational fluctuation. Qch adopts the largest *R*_g_ inside the condensate, whereas Flx remains the most compact. This ordering is opposite to what might be expected from dense-phase concentration alone and indicates that the more thermodynamically stable Qch condensate stretches chains more strongly through persistent interchain interactions, whereas the Flx condensate packs chains at higher density while maintaining smaller single-chain dimensions.

Temperature further distinguishes the three models. Condensation buffers the *R*_g_ distribution against temperature changes, especially in Qch (Figure S23A–P). In Qch, increasing temperature even decreases *R*_g_ inside the condensate (Figure S23D,H), consistent with weakened interchain attractions allowing chains to recover more isolated-like dimensions. Because the absolute standard deviation of *R*_g_ depends on the mean chain size, we also analyzed the coefficient of variation, cv.(*R*_g_) = std.(*R*_g_) /⟨*R*_g_⟩. This dimensionless quantity reports the relative breadth of the chain-size distribution. Although cv.(*R*_g_) varies with position in the slab and shows pronounced surface- and center-associated features in Qch (Figures S22U–X, and S23Q–S), it is nearly invariant among Qch, Mid, and Flx inside the condensate and remains weakly temperature dependent (Figures 2I, S22Y–AB, and S23T–V). Therefore, the dense phase partially erases the imposed differences in relative *R*_g_ fluctuation, even though it preserves strong differences in mean chain expansion. This convergence may reflect the melt-like polymeric environment within the condensate, where strong chain overlap and abundant interchain contacts partially screen the model-specific intrachain interactions that dominate isolated-chain fluctuations, driving individual chains toward near-ideal, Gaussian-like conformational statistics.^50–52^ Such collective conformational buffering may also account for the weak temperature dependence of cv.(*R*_g_) observed within the dense-phase regime.

Intrachain distance distributions provide a residue-level view of this remodeling. Compared with isolated chains, intrachain residue-pair distances *d*_*ij*_ increase broadly inside the condensate, particularly for sequence separations |*i*–*j*| > 10 residues (Figure S24). This expansion reflects the need for chains to engage multiple intermolecular contacts in the dense phase. At 450 K, however, the condensed and isolated distance profiles of Flx become similar, suggesting that isolated Flx chains approach an approximate Θ-like condition near this temperature.^53^ Importantly, the ordering of intrachain distances inside the condensate differs from that in the isolated state. Whereas isolated Flx chains combine shorter short-range distances with longer long-range distances, condensed-phase *d*_*ij*_ decreases from Qch to Mid to Flx across nearly all sequence separations (Figure S25). Temperature also acts differently across the three models: heating decreases *d*_*ij*_ in Qch, has little effect in Mid, and increases *d*_*ij*_ in Flx (Figure S26). These trends are consistent with the *R*_g_ analysis and show that the condensate environment couples conformational fluctuation to length-scale-dependent chain deformation.

The thermodynamic weakening of Flx raises a further question: does a less stable condensate also become structurally less ordered? To address this, we represented each FUS-LCD chain by a unit vector **χ** along its first principal axis, with the vector direction assigned from the N-terminus to the C-terminus (Figure 3A). We then calculated the orientation angle θ between this chain vector and the slab-normal *z* axis as a function of the chain center-of-mass position (Figure 3B). Near the interface, chains tend to align parallel to the interface. This may reflect an interfacial free-energy effect, although the present analysis does not directly resolve its microscopic origin. However, in the condensate interior the θ distribution is far less specific. Moreover, the mean value of θ indicates that the C-terminus of the chain preferentially points toward the condensate interior (Figure S27). To better capture the specificity of the chain angular distribution, we computed, at each *z*-position, the difference between the observed θ distribution and that expected for a completely random orientation. With θ expressed in degrees, the random-orientation distribution is $f_{\mathrm{rand}}\left(\theta \right)=\frac{\pi }{360}\sin\left(\frac{\pi \theta }{180}\right)$ . We used the Fisher–Rao distance *d*_FR_ to quantify the difference between the two probability density functions (Figures 3C and S28). The results confirm that the θ distribution near the interface deviates strongly from random, whereas in the condensate interior, particularly at the center, the distribution is very close to random. From Qch to Flx, the θ distribution becomes progressively more random, mirroring the trend observed with increasing temperature (Figure S29). This indicates that more flexible IDPs indeed adopt a more disordered arrangement inside the condensate.

**Figure 3:**
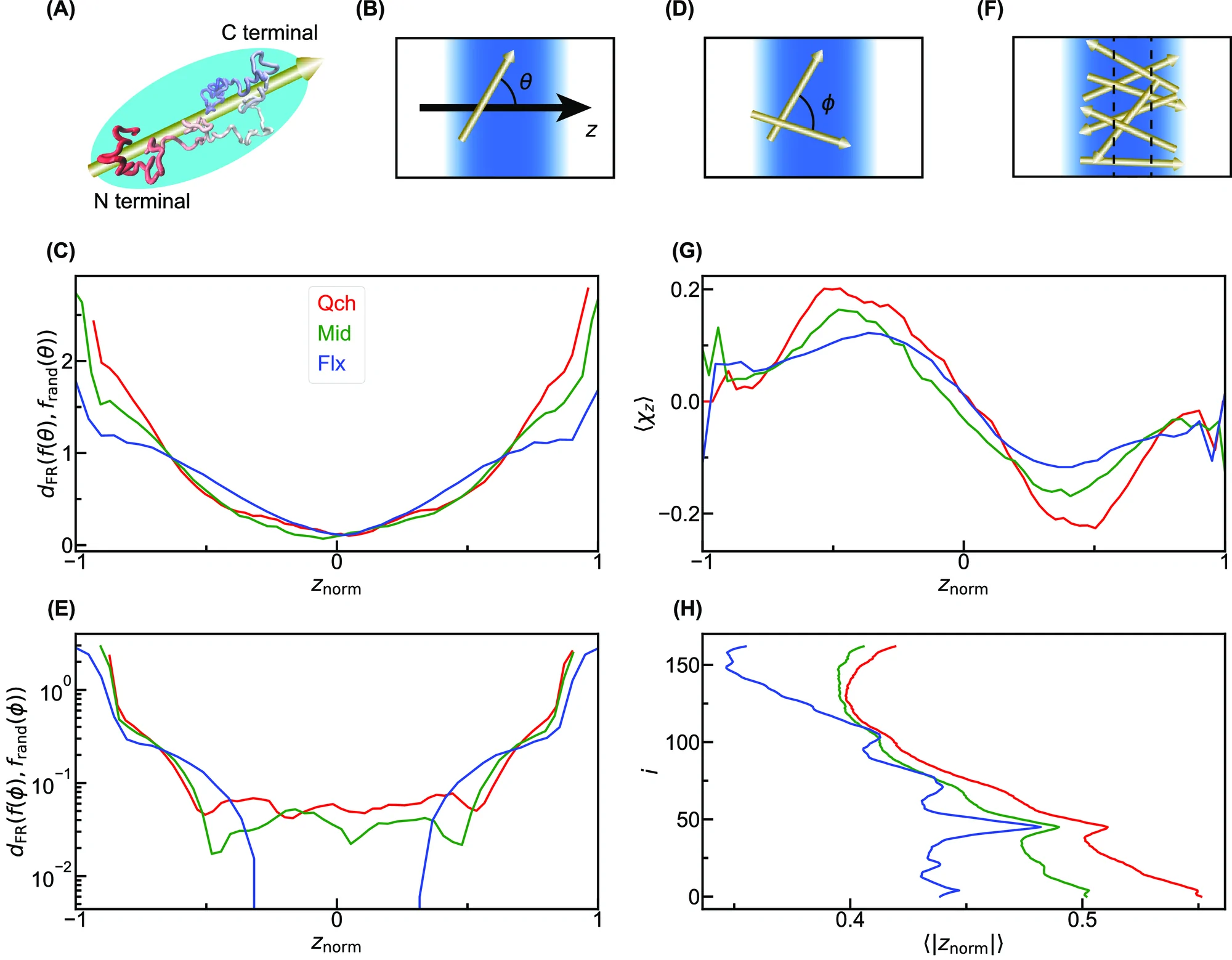
Orientational order and residue positioning in Qch, Mid, and Flx condensates at 300 K. (A) Schematic representation of a FUS-LCD chain as an orientation vector along its first principal axis, directed from the N-terminus to the C-terminus. (B) Definition of the orientation angle θ between the chain vector and the slab-normal *z* direction. The blue region denotes the condensed phase and the black border denotes the simulation box. (C) Fisher–Rao distance *d*_FR_ between the measured θ distribution and the random-orientation distribution, along the normalized slab coordinate *z*_norm_. (D) Definition of the relative orientation angle ϕ between two contacted chains. (E) Fisher–Rao distance *d*_FR_ between the measured ϕ distribution and the random-orientation distribution. (F) Definition of local orientational polarization. For a position *z* and bin width d*z*, the distribution of components of chain-orientation vectors **χ**(*z*) whose centers of mass lie within the bin represents the polarization. (G) Position-dependent mean local *z*-polarization, ⟨χ_*z*_⟩. (H) Residue-index dependence of the mean absolute normalized position, ⟨|*z*_norm_|⟩.

We further examined the relative orientation between connected chains by computing the distribution of the angle ϕ between their principal axes as a function of the midpoint of their centers of mass (Figure 3D). Two chains *I* and *J* were connected when their interchain contact sum, ∑_*i*∈*I*,*j*∈*J*_ *p*_*ij*_, exceeded 1. The ϕ distribution is closer to random in the condensate interior; near the interface, it is somewhat less random because of the constraint favoring alignment parallel to the interface (Figure S30). Likewise, from Qch to Flx the ϕ distribution becomes increasingly random, similar to the effect of raising temperature (Figures 3E, S31, and S32). This further confirms that more flexible chains exhibit a more disordered interchain arrangement within the condensate.

To test whether these angular biases produce collective vector alignment, we defined a local orientational-polarization metric. Treating each chain vector as a unit dipole, we computed the distribution of its components χ_*x*_, χ_*y*_ and χ_*z*_ at each *z*-position (Figure 3F). In the *x*- and *y*-directions, the dipoles show no polarization owing to isotropy (Figures S33 and S34). In the *z*-direction, however, a clear polarization emerges: the C-terminal end of the dipole tends to point toward the condensate interior, and this polarization is most pronounced for Qch, consistent with the preceding conclusions (Figures 3G, S35, and S36). Moreover, the fluctuations of the *z*-polarization are markedly suppressed near the interface, especially for Qch (Figures S36 and S37), which supports the conclusion of a relatively stable alignment parallel to the interface.

Notably, this analysis of orientation and orderliness is not dependent on the geometric shape of the slab condensate. We analyzed the relevant quantities in spherical condensates (Figure S38), where we used the radial axis *r* as the reference direction to calculate θ and χ_*r*_, and the results obtained were consistent with those in the slab model (Figures S39 and S40).

The polar orientation of chains is accompanied by sequence-dependent residue positioning. The N-terminal side of FUS-LCD tends to localize closer to the condensate exterior, whereas the C-terminal side is biased toward the interior (Figures 3H and S41). This asymmetry is mainly associated with the negatively charged Asp residues at positions 5 and 46. In particular, Asp46 is enriched toward the exterior and shifts neighboring residues in the same direction. Apart from these charge-associated effects, residue localization shows only weak dependence on residue type; outward enrichment of residue types such as Met, Ala, and Thr mainly reflects their proximity to the displaced Asp residues rather than an independent residue-type preference (Figures S42, S43E–H, and S44D–F). Because the Flx condensate has a more compact density profile, its residues are also positioned closer to the slab center on average (Figure S43A–D). Increasing temperature reduces the terminal asymmetry (Figure S44A–C).

Finally, we analyzed the condensate as a chain-contact network to determine how conformational fluctuation changes intermolecular connectivity. Each chain was treated as a node, and two chains *I* and *J* were connected by an edge when their interchain contact sum, ∑_*i*∈*I*,*j*∈*J*_ *p*_*ij*_, exceeded 1 (Figure 4A). From Qch to Mid to Flx, the mean node degree decreases, indicating that each chain contacts fewer neighbors on average (Figures 4B and S45A–H). This decrease is consistent with the smaller condensed-phase *R*_g_ of Flx, which reduces the spatial range over which a chain can contact surrounding molecules. However, the Flx network is not simply less connected. Its betweenness centrality is more heterogeneous, with a larger skewness indicating that a subset of chains acts as bridge-like connectors between otherwise less directly connected regions (Figures 4C, and S45I–X). Flx also has the largest small-world coefficient *c*_swn_, indicating a topology that combines local clustering with efficient network-spanning connectivity (Figures 4D and S45Y–AB). Consistent with other features, these network characteristics of Flx resemble the high-temperature limit (Figure S46). Conformational fluctuation therefore cannot be understood as a simple rescaling of temperature or interaction strength. Instead, it produces a distinct condensate state: dense but thermodynamically fragile, less orientationally ordered, less uniformly connected, and organized by a heterogeneous contact network.

**Figure 4:**
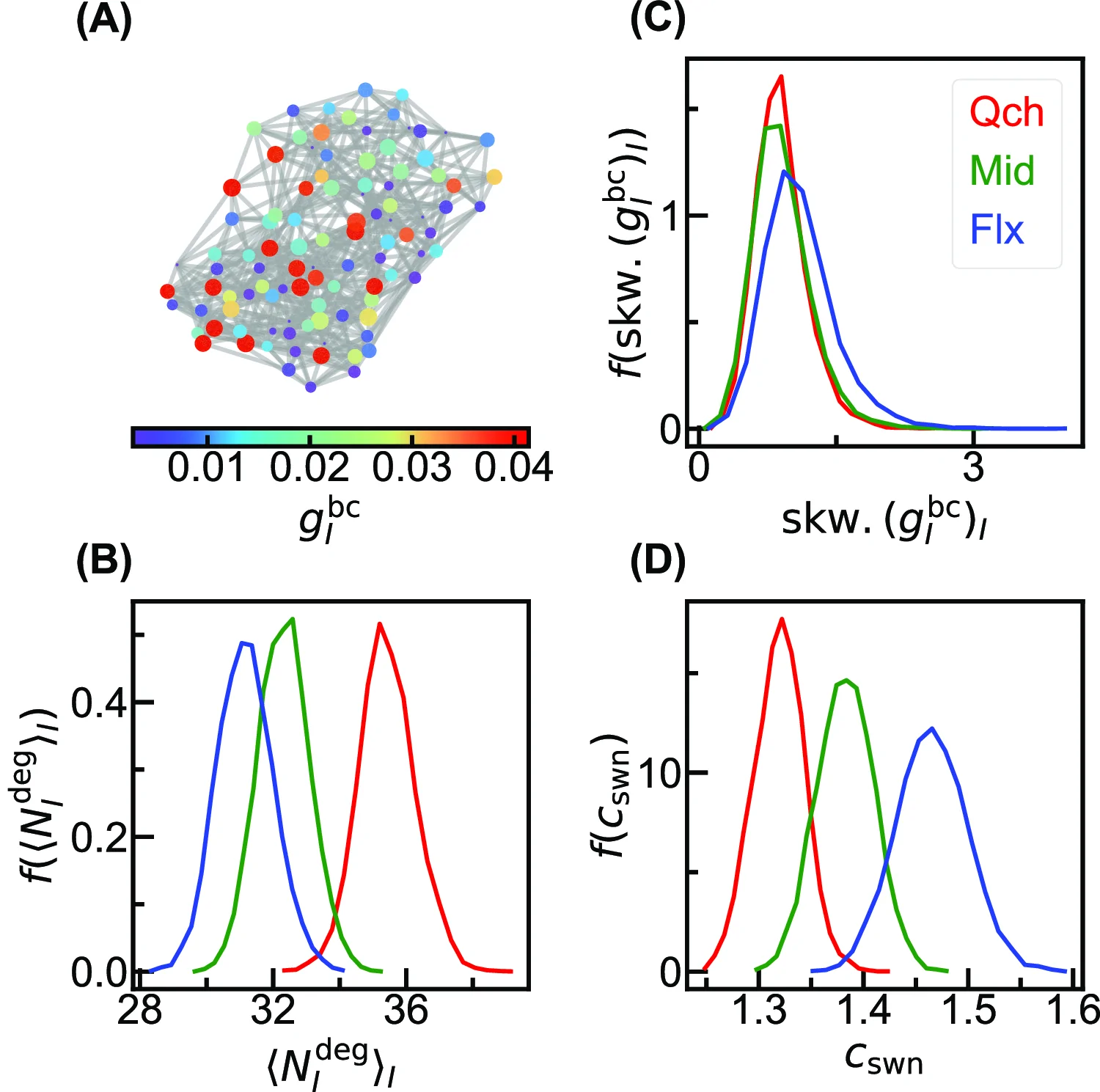
Chain-contact network topology in Qch, Mid, and Flx condensates at 300 K. (A) Representative chain-contact network obtained by thresholding chain-pair contact sums. Node size reports node degree *N*_*I*_^deg^, and node color reports betweenness centrality *g*_*I*_^bc^. (B) Probability distributions of the system-level mean node degree ⟨*N*_*I*_^deg^⟩_*I*_. (C) Probability distributions of the system-level skewness of betweenness centrality, skw.(g_*I*_^bc^)_*I*_. (D) Probability distributions of the system-level small-world coefficient *c*_swn_.

### Conformational Fluctuation Accelerates Condensate Transport and Viscoelastic Relaxation

The thermodynamic and network analyses above indicate that increasing conformational fluctuation produces condensates that are less stable but more heterogeneously connected. We therefore asked how this altered network organization affects molecular mobility and material relaxation. Because the slab geometry is anisotropic, we analyzed chain center-of-mass motion separately in the interfacial plane (*xy*) and along the slab-normal direction (*z*) (Figures S47 and S48A–D). The center-of-mass mean-squared displacement (MSD) reports whole-chain translational motion within the condensate, whereas the lag-time-dependent apparent diffusion coefficient *D*(Δ*t*) provides a direct comparison of mobility over different observation windows.^54^ We use an apparent diffusion coefficient rather than a single constant diffusion coefficient because the condensate environment and slab geometry give rise to non-Fickian behavior. Motion along *z* is subdiffusive, as shown by the decrease of *D*_*z*_(Δ*t*) with increasing lag time, whereas in-plane motion is closer to normal diffusion and can appear weakly superdiffusive over part of the accessible time range, consistent with previous observations in condensate simulations.^54^

Across models, chain mobility increases from Qch to Mid to Flx over the short- and intermediate-time windows that are least affected by finite-slab confinement (Figures 5A and S48E–H). At longer lag times, especially for Δ*t* > 10 ns, the apparent *z*-direction diffusivity of Flx decreases below that of Qch and Mid. We attribute this long-time reversal to the thinner Flx slab, which imposes stronger confinement along the slab-normal direction. Thus, the long-time *z*-direction diffusivity should be interpreted together with the density profile and slab thickness. Despite this confinement effect, the overall trend is clear: the high-fluctuation condensate permits faster molecular transport. Diffusion increases with temperature in all three models, and the temperature sensitivity is strongest for Flx (Figure S49). Residue-level MSD analyses show the same qualitative ordering, indicating that the enhanced mobility of Flx is not restricted to center-of-mass translation but also appears at the local segmental level (Figures S50–S52).

**Figure 5:**
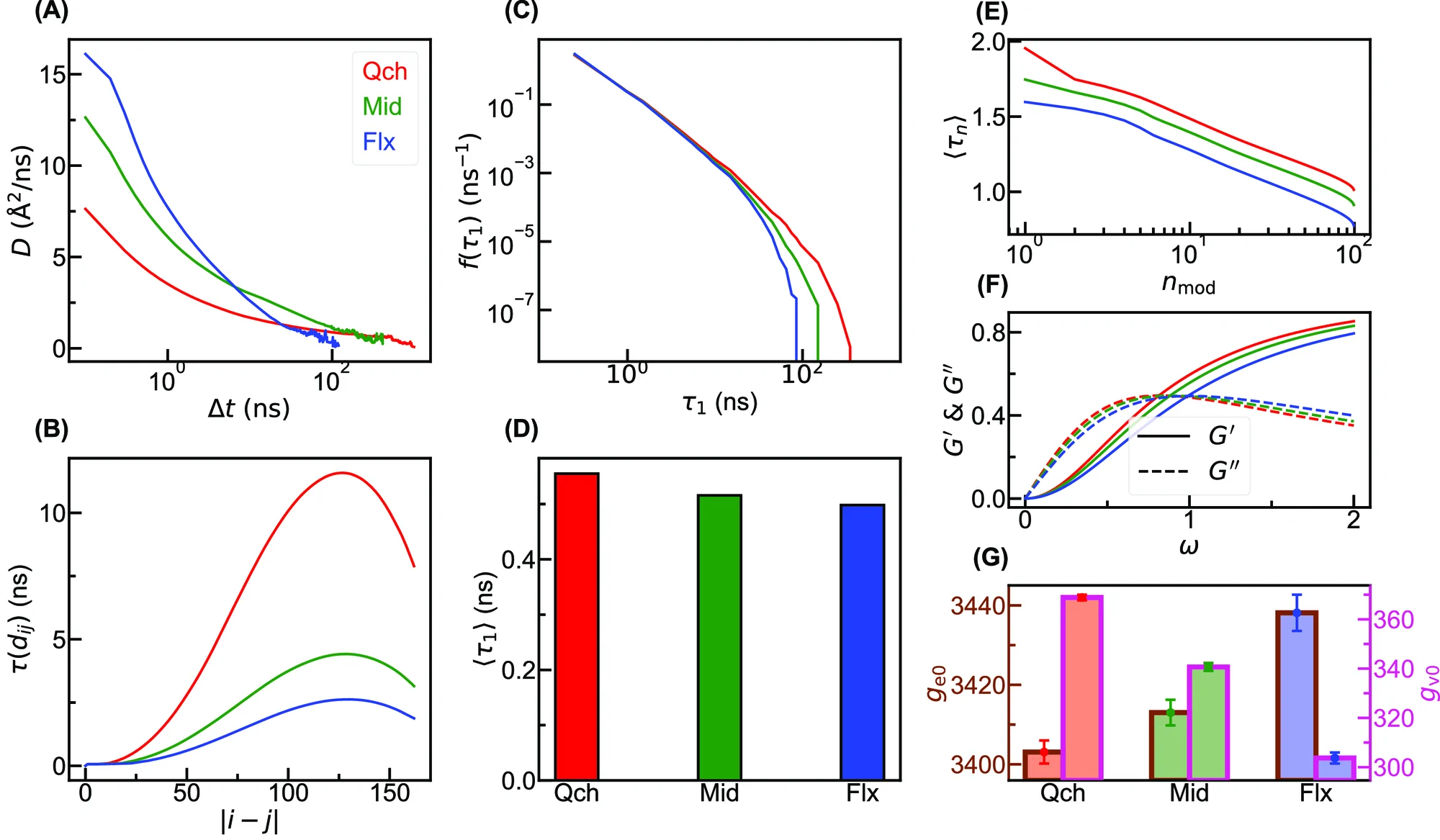
Molecular transport, contact exchange, and viscoelastic relaxation in Qch, Mid, and Flx condensates at 300 K. (A) Lag-time-dependent apparent diffusion coefficient *D*(Δ*t*) along the slab-normal direction. (B) Relaxation time τ­(*d*_*ij*_) of intrachain residue-pair distance autocorrelation as a function of sequence separation |*i*–*j*|. (C) Probability density of chain-pair contact lifetimes τ_1_. (D) Average contact lifetime ⟨τ_1_⟩; for the strongly skewed lifetime distribution, the average level is evaluated in log space as described in the Supporting Methods. (E) System-level mode relaxation time ⟨τ_*n*_⟩ as a function of mode index *n*_mod_. (F) Storage and loss moduli, *G*′(ω) and *G* ^″^(ω), estimated from the network-mode relaxation spectrum. (G) Elastic and viscous prefactors, *g*_e0_ and *g*_v0_, derived from the relaxation spectrum.

We next examined internal chain reorganization inside the condensate. For each residue pair, we calculated the distance autocorrelation function *f*_d_*ij*__^ac^ and extracted the relaxation time τ­(*d*_*ij*_) as the lag time at which the autocorrelation decays to 1/*e* (Figure S53). Shorter τ­(*d*_*ij*_) values indicate faster loss of memory of intrachain distances and therefore faster internal structural rearrangement. Consistent with the enhanced mobility and smaller condensed-phase intrachain distances of Flx, τ­(*d*_*ij*_) decreases from Qch to Mid to Flx across most sequence separations (Figures 5B and S54). A sharp decrease in τ­(*d*_*ij*_) is also observed at the largest sequence separations, similar to the isolated-chain case, reflecting the high mobility of terminal regions. Although the condensate environment buffers chain dimensions, it does not eliminate temperature-dependent internal relaxation. The temperature dependence of τ­(*d*_*ij*_) remains evident in all models, but is weakest for Flx (Figure S55). Thus, Flx shows strong temperature sensitivity in translational diffusion and thermodynamic stability, but comparatively weak temperature sensitivity in intrachain distance relaxation.

To connect these single-chain dynamics to intermolecular exchange, we quantified the lifetimes of the binary interchain contact states used to define the chain-contact network. A contact lifetime measures how long a pair of chains remains connected before the edge breaks; shorter lifetimes therefore indicate a more labile and rapidly exchanging network. The contact-lifetime distributions are broad and close to power-law-like (Figure 5C), indicating that interchain dissociation is not governed by a single characteristic bond lifetime but by a hierarchy of local environments and contact strengths.^55^ Because these distributions are strongly skewed, we summarize their average level in log space, as described in the Supporting Methods. Flx has the shortest average contact lifetime, consistent with its faster chain diffusion and shorter intrachain relaxation times (Figures 5D, and S56F–J). The shorter contact lifetime is accompanied by a longer average noncontact lifetime (Figure S56E), indicating that once a pair of Flx chains separates, it is less likely to re-establish contact rapidly. At the same time, the far tails of both contact and noncontact lifetime distributions are less extended in Flx (Figure S56A–D,F–I), suggesting fewer extremely persistent pair states. As expected for thermally activated exchange, heating shortens contact lifetimes and lengthens noncontact lifetimes in all three models (Figure S57).

Finally, we asked whether these molecular and network-level dynamics translate into altered viscoelastic relaxation. We estimated condensate-level relaxation using a Zimm-type network description based on the chain-contact graph.^56–60^ The mode relaxation time τ_*n*_ reports how rapidly a collective network mode dissipates stress. Across the relaxation spectrum, τ_*n*_ decreases from Qch to Mid to Flx (Figures 5E, S58 and S59A–D). This ordering is consistent with the shorter intrachain relaxation times and shorter interchain contact lifetimes of Flx, and indicates that stress stored in the Flx contact network relaxes more rapidly.

The corresponding storage and loss moduli, *G*′(ω) and *G* ^″^(ω), further support this conclusion. *G*′(ω) quantifies elastic energy storage, whereas *G* ^″^(ω) quantifies viscous dissipation. The frequency at which the two curves cross marks the transition between predominantly elastic and predominantly viscous response. Flx exhibits a higher crossover frequency than Qch and Mid (Figures 5F and S59E–H), corresponding to a shorter characteristic relaxation time. At the same time, Flx has a larger steady-state compliance *g*_e0_ but a smaller viscous prefactor *g*_v0_ (Figures 5G and S59I,J). This combination suggests that Flx can transiently store elastic stress through an organized contact network, but that the stress is released rapidly because contacts are short-lived and network modes relax quickly.^61^ The temperature response of the relaxation spectrum is not captured by a single trend: the overall mode relaxation times and moduli of Flx are the least temperature-sensitive, whereas its viscous prefactor is the most temperature-sensitive (Figure S60). Therefore, conformational fluctuation fluidizes the condensate primarily by accelerating exchange and stress relaxation, rather than by simply weakening every component of the material response.

Together, these analyses show that increasing single-chain conformational fluctuation shifts FUS-LCD condensates toward a more dynamic material state. Flx chains diffuse faster over the accessible short- and intermediate-time windows, reorganize intrachain distances more rapidly, form shorter-lived interchain contacts, and dissipate network stresses at higher frequencies. This dynamic fluidization provides a mechanistic bridge between the weaker thermodynamic stability described above and the altered condensation kinetics analyzed below.

### Conformational Fluctuation Delays Nucleation but Accelerates Cluster Coalescence

We next asked how conformational fluctuation affects the pathway by which dispersed chains assemble into a condensate. To this end, we initiated condensation simulations from 50 spatially separated FUS-LCD chains with minimal initial interchain contacts. As interchain attractions drive assembly, chains form progressively larger connected components in the contact network. We quantified this process by the number of clusters, *N*_clst_, where chains belonging to the same connected component were assigned to the same cluster (Figures 6A and S61A–D). At first sight, the decay of *N*_clst_ appears to slow from Qch to Mid to Flx. However, the required time *t*_req_ to reach fixed cluster numbers reveals two distinct kinetic regimes. Flx requires a longer time to reach 25 clusters, corresponding approximately to the first half of the clustering process (Figure 6B), but reaches the final single-cluster state on a comparable time scale to Qch and Mid (Figures 6C and S61F–I). Thus, the delayed early clustering of Flx must be compensated by faster late-stage coalescence.

**Figure 6:**
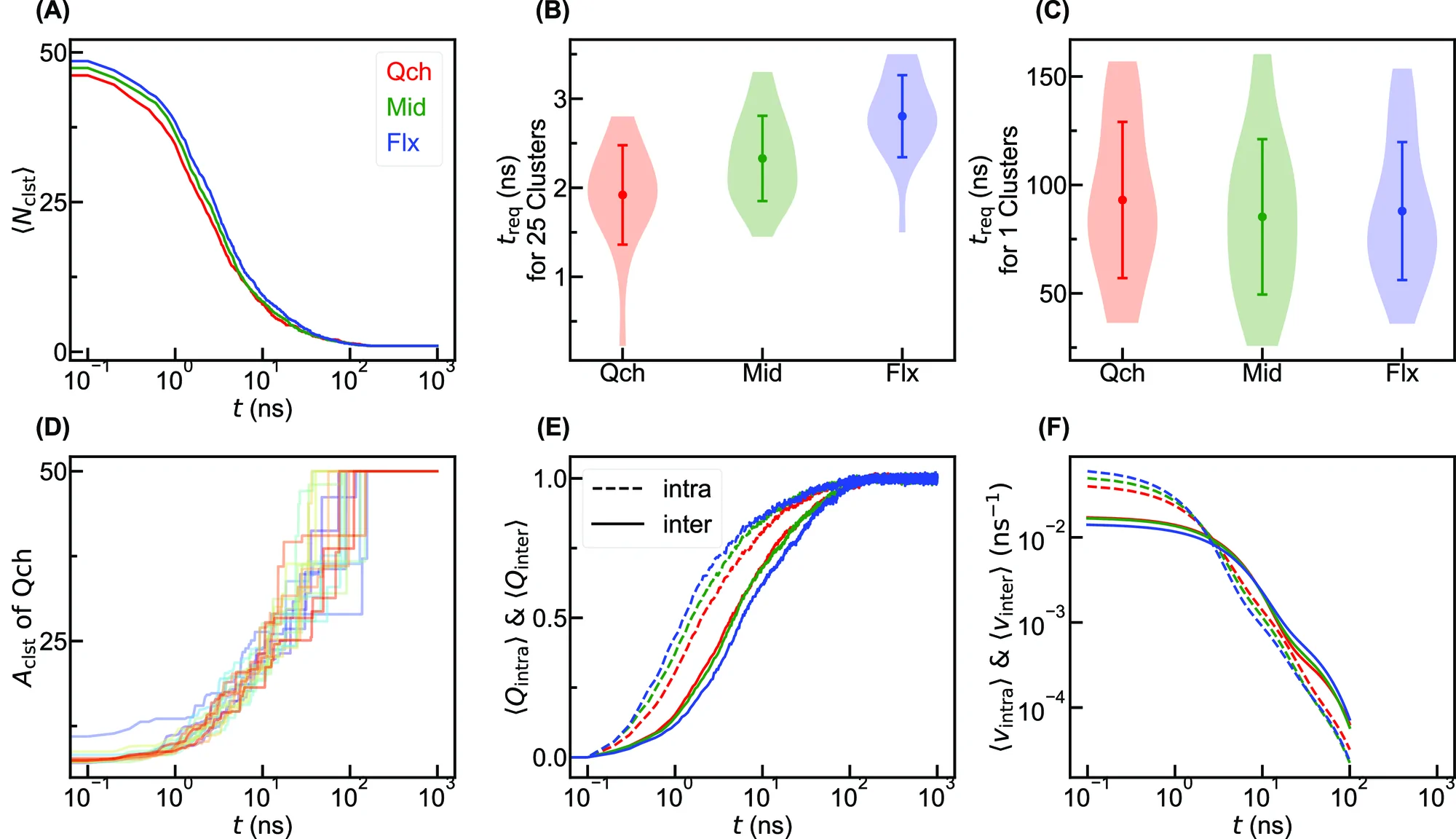
Condensation kinetics from 50-chain simulations of Qch, Mid, and Flx at 300 K. (A) Mean number of chain clusters, ⟨*N*_clst_⟩, as a function of time. Chains in the same connected component of the interchain contact network are assigned to the same cluster. (B) Distributions of the required time *t*_req_ to reach 25 clusters across independent repeats, reporting early stage clustering. (C) Distributions of *t*_req_ to reach one cluster across independent repeats, reporting completion of condensation into a single connected cluster. (D) Cluster-size amplitude *A*_clst_ for Qch, shown as a representative example; large jumps indicate mergers involving large clusters. (E) Normalized intrachain and interchain contact-formation variables, ⟨*Q*_intra_⟩ and ⟨*Q*_inter_⟩, during condensation. (F) Fitted formation rates ⟨*v*_intra_⟩ and ⟨*v*_inter_⟩ for intrachain and interchain contacts during early and late stages of condensation.

The same crossover is evident from the cluster-growth rate *v*_clst_, calculated from the fitted time dependence of *N*_clst_. Flx changes more slowly during the early stage but more rapidly after the crossover (Figure S61E). Because the *N*_clst_(*t*) curves are exponential-like but not well described by a single time scale, we fitted them with a double-exponential function. This fit captures the trajectories substantially better than a single-exponential form (Figure S61A–C), supporting a phenomenological separation into a fast early component and a slower late component with characteristic rate constants of order 0.4 ns^–1^ and 0.04 ns^–1^, respectively. We interpret these two components as collective descriptors of early nucleation/capture and late coalescence rather than as elementary reaction rates. Under this interpretation, Flx has a smaller early stage rate constant but a larger late-stage rate constant, consistent with delayed initial clustering followed by accelerated merger of larger clusters.

An analysis of isolated chains, *N*_iso_, supports this stage assignment. In contrast to *N*_clst_, the decay of *N*_iso_ is adequately described by a single-exponential function; adding a second exponential component provides only marginal improvement. The fitted rate constant for isolated-chain absorption is smallest for Flx, consistent with its slower early decrease in *N*_clst_. This indicates that the early kinetic regime is dominated by the capture of isolated chains and the formation of small clusters, whereas the accelerated late-stage decrease in *N*_clst_ mainly reflects mergers among already formed clusters.

To distinguish small-cluster nucleation from large-cluster coalescence more directly, we introduced the cluster-size amplitude *A*_clst_. Unlike *N*_clst_, which decreases by one for any binary merger regardless of cluster size, *A*_clst_ changes weakly when two small clusters merge but strongly when larger clusters coalesce. Consistent with this design, *A*_clst_ changes only modestly during the early stage but exhibits large jumps during the late stage (Figures 6D, and S61N–P). These observations support a two-step physical picture. In Flx, weaker persistent interchain association makes early nucleation and isolated-chain capture less favorable. Once larger clusters have formed, however, the lower interfacial penalty and faster internal relaxation of Flx clusters facilitate deformation, rearrangement, and fusion, thereby accelerating late-stage coalescence.

Finally, we compared how intrachain and interchain contacts adapt during condensation. We calculated normalized contact-formation variables, *Q*_intra_ and *Q*_inter_, which report the progress of intrachain and interchain contact formation from the initial dispersed state to the final condensed state. Intrachain contact reorganization is much faster than interchain contact formation in all three models (Figure 6E), indicating that individual chains rapidly adjust their internal conformations before the interchain contact network fully develops. During the early stage, Flx shows the largest intrachain contact-formation rate, ⟨*v*_intra_⟩, but the smallest interchain contact-formation rate, ⟨*v*_inter_⟩ (Figure 6F). Thus, enhanced conformational flexibility promotes rapid internal reorganization but does not immediately produce stable intermolecular capture. During the late stage, the ordering reverses: Qch shows faster intrachain reorganization, whereas Flx favors intercluster contact formation associated with large-cluster coalescence. Together, these results show that conformational fluctuation regulates condensation kinetics in opposite ways at different stages of assembly. High fluctuation slows early nucleation by reducing persistent interchain capture, but accelerates late coalescence by enabling rapid deformation and reorganization of larger clusters.

## Discussion

This study identifies the breadth of the single-chain conformational ensemble as an independent physical variable that regulates LLPS. Previous work has established that dilute-phase single-chain properties contain substantial information about phase-separation propensity, and that CG models optimized against single-chain observables can improve predictions of IDP phase behavior.^26–28^ ME approaches further reinforce this ensemble view by treating IDP conformations as probability distributions that can be minimally refined without collapsing the ensemble to one representative structure.^44,45^ Here, we used this principle to construct three models of the same FUS-LCD sequence with nearly identical mean radius of gyration, ⟨*R*_g_⟩, but different widths of the *R*_g_ distribution. Because Qch, Mid, and Flx have the same amino-acid sequence and comparable average chain dimensions, the observed differences in thermodynamics, network topology, dynamics, viscoelasticity, and condensation kinetics cannot be attributed simply to chain length, sequence composition, or mean compaction. Instead, they reveal conformational fluctuation as an ensemble-level descriptor that complements sticker valence, sticker patterning, charge patterning, and intrinsic chain stiffness in controlling condensate properties.^20,21,53^

A useful way to interpret the Qch–Flx difference is through the balance between local and nonlocal conformational correlations. In the isolated state, Flx displays shorter residue-pair distances at small sequence separations but longer distances at large sequence separations, whereas Qch shows the opposite trend. Thus, Flx is not simply a more expanded or more disordered version of Qch. Rather, it is locally compact yet globally extensible. Conversely, Qch is less locally compact but more constrained by longer-range intrachain correlations. We therefore propose that the three models span a continuum from a nonlocal-correlation-dominated ensemble to a local-contact-dominated ensemble. Transient local structural preferences, if weakly coupled to global chain collapse, may generate Flx-like behavior, whereas persistent nonlocal contacts or long-range intrachain coupling may generate Qch-like behavior. In this sense, local order and global order are separable. A chain can be locally organized while remaining globally flexible, and this separation can strongly affect LLPS.^30,32,62,63^ Qch and Flx should therefore be viewed as phenomenological ensemble classes rather than as labels for particular sequences. Real IDPs exhibit strongly sequence-dependent conformational heterogeneity, and similar average chain dimensions can coexist with distinct size and shape fluctuations; proteome-scale studies further show that conformational entropy is modulated by sequence features including charge and hydropathy patterning and residue stickiness.^17,64,65^ We therefore expect sequences with more persistent nonlocal intrachain correlations to exhibit more Qch-like behavior, whereas sequences with competing local and long-range interactions and broader conformational heterogeneity may be more Flx-like, although assigning individual IDPs to these regimes will require sequence-specific characterization of their full conformational ensembles. This local-to-nonlocal correlation picture, and its consequences for nucleation, coalescence, orientational ordering, and viscoelastic relaxation, are summarized in Figure 7.

**Figure 7:**
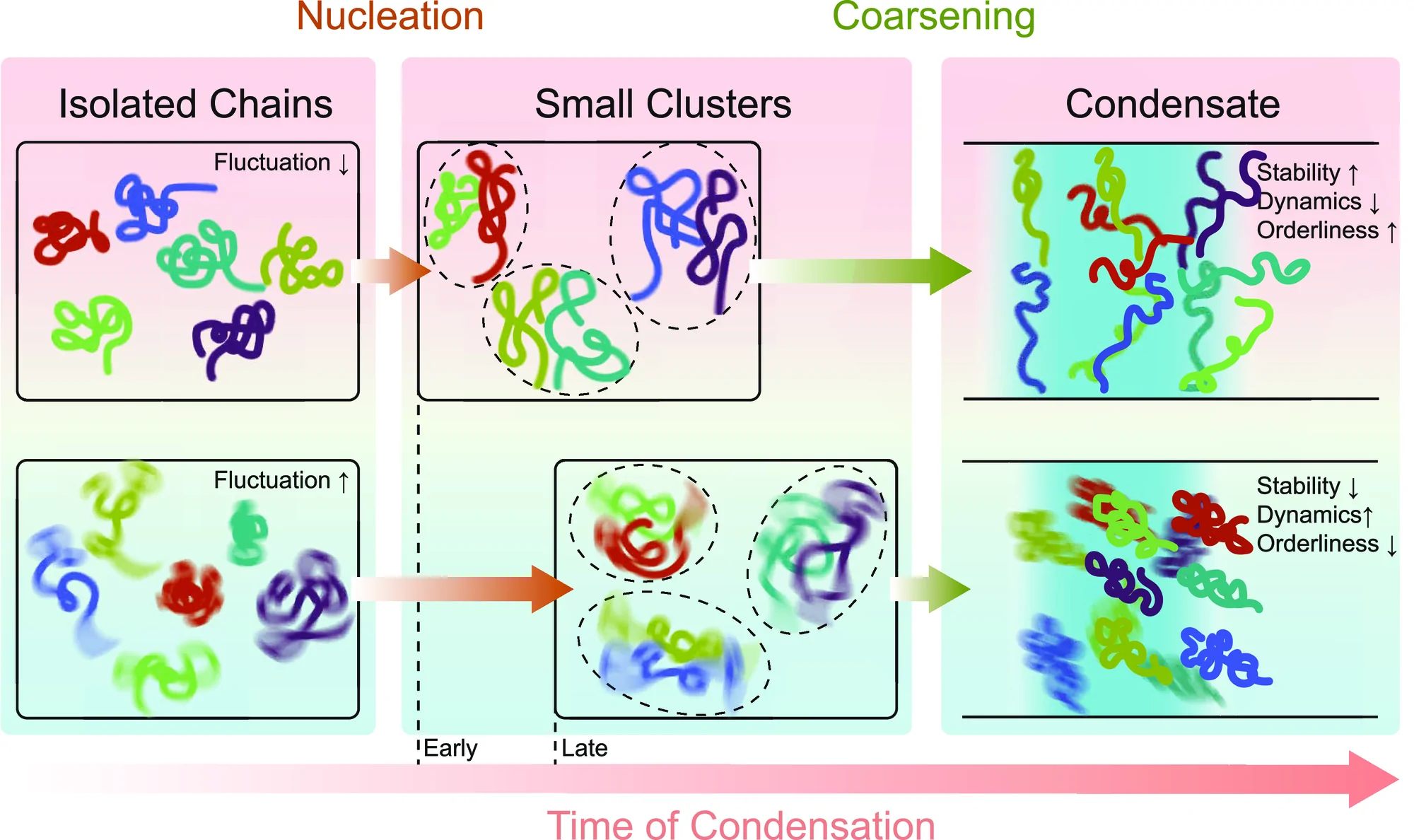
Schematic overview of how single-chain conformational fluctuation regulates LLPS. The top row represents an ensemble with lower isolated-state fluctuation, corresponding to Qch. The bottom row represents an ensemble with higher isolated-state fluctuation, corresponding to Flx. The horizontal direction schematically represents progression from isolated chains to early clusters and finally to a mature condensate. During condensation, the ensemble with lower isolated-state fluctuation favors earlier nucleation and yields a thermodynamically more stable condensate with slower interior dynamics and higher orientational order, particularly at the interface. By contrast, the ensemble with higher isolated-state fluctuation exhibits delayed nucleation and yields a thermodynamically less stable condensate with faster interior dynamics and lower orientational order.

This interpretation is especially relevant for FUS-LCD. FUS-family proteins are often discussed through a molecular grammar in which tyrosine, arginine, glutamine, serine, glycine, and charged residues tune LLPS, condensate aging, and hardening.^20,21,42^ NMR, Raman spectroscopy, mutagenesis, and simulations have shown that FUS-LCD condensates remain conformationally heterogeneous and are stabilized by many weak, transient, and distributed interactions rather than by a single folded condensed-phase structure.^18,39–41,43^ Our ME-CG model is consistent with this view because it preserves AA-derived contact propensities while allowing the breadth of the conformational ensemble to be varied. The finding that Flx combines local compaction with global extensibility provides a mechanistic explanation for why ensembles with the same ⟨*R*_g_⟩ can nevertheless produce distinct condensates. Mean chain size reports the average spatial extent of an ensemble, but it does not specify whether compactness arises from many local contacts, a few long-range contacts, or a broad mixture of conformational states.

The thermodynamic results show that dense-phase concentration alone is an incomplete measure of condensate stability. Flx forms the most concentrated dense phase at low temperature, but has the lowest critical temperature and surface tension. Thus, Flx is dense but thermodynamically fragile. This behavior is consistent with the general picture of associative biomacromolecules, in which LLPS reflects a balance among multivalent energetic contacts, conformational entropy, solvation, and network connectivity rather than concentration alone.^13^ Previous CG simulations of FUS-LCD also showed that modest changes in effective protein–protein attraction can strongly alter phase behavior and surface tension,^36^ and studies of regulated condensates have linked changes in phase equilibrium to changes in interfacial tension.^66^ A limitation of the present model is that the absolute dense-phase concentrations are higher than experimental estimates for FUS-LCD, which are typically in the range of approximately 0.32–0.48 g/mL.^40,67^ Because the CG interactions are optimized against AA-derived intrachain contact propensities, residual force-field or sampling biases toward overly compact IDP conformations can be inherited by the CG model and lead to overly strong effective association and hence elevated condensed-phase densities.^68–71^ Accordingly, the present results should be interpreted primarily in terms of the relative trends among Qch, Mid, and Flx rather than as quantitative predictions of absolute condensate concentrations. At the chain level, the expansion of *R*_g_ upon condensation is consistent with simulations of prion-like LCDs showing that chains can adopt expanded conformations within condensates and near interfaces.^37^ Our results add an important qualification: the magnitude and consequence of this expansion depend on the pre-existing breadth of the isolated-chain ensemble. In Qch, stronger nonlocal correlations and more persistent intermolecular contacts appear to stretch chains more strongly in the condensate, producing larger condensed-phase *R*_g_ and greater thermodynamic stability. In Flx, local compaction allows chains to pack densely, but weaker global coupling and shorter-lived contacts make the dense phase easier to disrupt.

The dynamical, contact-lifetime, and network analyses provide a molecular basis for this density–stability decoupling. From Qch to Flx, chains diffuse faster over the accessible short- and intermediate-time windows, intrachain distances relax more rapidly in the condensate, interchain contacts become shorter-lived, and collective network modes relax at higher frequencies. These trends support the emerging view that condensates are dynamic multivalent networks whose transport and rheology are controlled by the lifetime, heterogeneity, and connectivity of transient contacts.^40,54,58–60,72^ The broad, nearly power-law-like contact-lifetime distributions indicate that contact dissociation is not governed by a single effective bond lifetime, but by a hierarchy of local environments.^55^ At the graph level, Flx has fewer average neighbors but larger betweenness heterogeneity and a larger small-world coefficient, echoing network-like organization reported for prion-like LCD condensates.^37^ Thus, conformational fluctuation does not simply weaken all interactions uniformly. Instead, it replaces a more uniformly connected and persistent network with a sparser, more heterogeneous, and more rapidly reorganizable one. This explains how Flx can remain dense and transiently load-bearing while relaxing stress faster and displaying more liquid-like transport.

Orientational organization provides another manifestation of the effect of conformational fluctuation on condensate structure. Although all three models exhibit a preference for chain alignment parallel to the interface, consistent with the structured interfaces reported for LCD condensates,^37^ this interfacial order is weaker in Flx and strongest in Qch. This observation is particularly relevant to the liquid-to-solid conversion of FUS condensates, which has been shown to initiate preferentially at condensate interfaces.^38^ The enhanced interfacial organization in Qch, together with its longer-lived interchain contacts and slower network relaxation, suggests that restricted conformational fluctuations may favor the persistent intermolecular organization associated with dynamical arrest, whereas the more rapidly reorganizing Flx ensemble may oppose such interfacial ordering. Although our simulations were not intentionally designed to probe long-time solidification, these results are consistent with the interface acting as a structurally organized region from which liquid-to-solid conversion may emerge.

The condensation kinetics further demonstrate that conformational fluctuation affects different assembly stages in opposite ways. Flx delays early clustering and isolated-chain capture, consistent with weaker persistent interchain association: individual encounters are less likely to survive long enough to form stable nuclei. This is compatible with the view that condensate nucleation is governed by a balance of supersaturation, surface tension, molecular specificity, and the lifetime of productive contacts.^73^ Once larger clusters have formed, however, Flx undergoes faster late-stage coalescence. Its lower surface tension should reduce the cost of interfacial deformation, and its faster internal relaxation should facilitate cluster deformation, fusion, and structural remodeling. This interpretation agrees with optical-tweezer studies showing that droplet fusion is governed by shear relaxation rather than by the simple viscocapillary ratio alone.^74^ It also connects to the broader idea that condensate architecture can be shaped by kinetic accessibility and dynamical arrest, not only by the equilibrium binodal.^75^ Thus, conformational fluctuation can slow nucleation while accelerating coarsening, providing a natural explanation for the two kinetic regimes observed here.

These findings have biological and materials-design implications. In cells, conformational fluctuation may be tuned by post-translational modifications, binding partners, chaperones, RNA, crowding, salt, and local sequence context.^76,77^ For FUS, arginine methylation and transportin regulate LLPS and gelation,^43^ and failure to control condensate material properties is linked to stress responses, aggregation, aging, and neurodegenerative disease.^9^ Our results suggest that such regulation need not act only by changing the number of stickers, the net attraction strength, or the mean compactness. It may also act by redistributing conformational correlations across sequence length scales. A perturbation that enhances local contacts while weakening long-range coupling could shift an IDP toward Flx-like behavior: locally organized, globally deformable, dynamically fluid, and prone to rapid coalescence after nucleation. Conversely, perturbations that stabilize persistent nonlocal contacts could produce more Qch-like behavior and, if sufficiently strong or long-lived, may promote gelation or kinetic arrest.

Overall, this work expands the sequence–ensemble–phase relationship by showing that the width and internal structure of the conformational ensemble can be as important as its mean size. Conformational fluctuation differentially regulates condensate properties, including dense-phase concentration, thermal stability, surface tension, molecular mobility, viscoelastic relaxation, orientational order, and condensation kinetics. Recent IDP design studies show that compaction and phase propensity can be tuned even at fixed composition, making matched-⟨*R*_g_⟩ tests experimentally realistic.^29^ Future experiments could design FUS-LCD variants or synthetic sticker–spacer polymers with similar ⟨*R*_g_⟩ but different *R*_g_ variance and different local-to-nonlocal contact ratios. Measurements combining SAXS, smFRET, NMR or PRE, FRAP, droplet-fusion assays, and microrheology could test whether the Flx-like and Qch-like regimes predicted here represent general design principles for programmable biomolecular condensates.^78^

## Methods

### Computational Strategy

The aim of this study was to isolate conformational fluctuation as a single-chain ensemble variable and determine how it regulates liquid–liquid phase separation (LLPS). We used the FUS low-complexity domain (FUS-LCD) as the model IDP. Rather than comparing different amino-acid sequences, we constructed three residue-level CG models of the same FUS-LCD sequence. These models were designed to have nearly identical mean radius of gyration, ⟨*R*_g_⟩, but different widths of the *R*_g_ distribution. This design separates the effect of ensemble breadth from changes in sequence composition, chain length, or average chain size.

Each residue was represented by one bead located at the C_α_ position. The CG potential contains bonded interactions, screened electrostatic interactions, excluded-volume repulsion, and a data-driven short-range nonbonded term. The residue-type-dependent short-range interaction coefficients were optimized using a maximum-entropy (ME) framework.^46–49^ In brief, all-atom (AA) simulations were used to estimate reference intrachain contact probabilities, and the CG interaction coefficients were iteratively updated until the CG contact map reproduced the AA-derived target at the residue-type level. This procedure preserves residue-level interaction information from the AA reference while keeping the model sufficiently simple for many-chain simulations.

To tune conformational fluctuation, we varied the characteristic length scales of the excluded-volume and short-range attractive interactions. For each choice of length-scale parameters, the ME optimization was repeated because the optimized residue-type interaction coefficients depend on the internal length scales of the CG potential. From these parameter scans, we selected three models, denoted Qch, Mid, and Flx, corresponding to low, intermediate, and high *R*_g_ fluctuation, respectively. The selected models were required to maintain similar ⟨*R*_g_⟩, so that the width of the *R*_g_ distribution, quantified by either std.(*R*_g_) or cv.(*R*_g_), was the main controlled variable. Single-chain simulations were then used to characterize conformational distributions, intrachain distance maps, residue-level dynamics, and distance relaxation times. Double-chain simulations were used to calculate the second virial coefficient. Multichain simulations were used to analyze phase diagrams, surface tension, chain organization, contact-network topology, diffusion, viscoelastic relaxation, and condensation kinetics.

### CG Force Field

The FUS-LCD sequence, corresponding to residues 1–163 of human FUS (UniProt ID: P35637), was represented as a bead–spring chain. Each bead corresponds to one residue and is placed at the C_α_ position. Adjacent beads are connected by a harmonic bond potential,

1 $$U_{b}\left(r_{i,i+1}\right)=K_{b}{\left(r_{i,i+1}-r_{b}\right)}^{2}$$

where *r*_*i*,*i*+1_ is the distance between adjacent beads *i* and *i* + 1, *K*_b_ is the bond force constant, and *r*_b_ is the equilibrium bond length. The bond parameters were set to *K*_b_ = 10.0 kcal/(mol·Å^2^) and *r*_b_ = 3.82 Å.

Electrostatic interactions were described by a Debye–Hückel potential

2 $$U_{\mathrm{ele}}\left(r_{\mathrm{ij}}\right)=\frac{q_{s_{i}}q_{s_{j}}}{4\pi \eta r_{\mathrm{ij}}}\exp\left(-\kappa r_{\mathrm{ij}}\right)$$

Here, *s*_*i*_ denotes the residue type of bead *i*, *q*_*s*_*i*__ is the residue charge, and *e* is the elementary charge. Arg and Lys were assigned charge + *e*, Asp and Glu were assigned charge −*e*, and all other residues were assigned zero charge. The dielectric constant was set to η = 80, and the inverse Debye length was set to κ = 0.1 Å^–1^, corresponding approximately to a physiological monovalent salt concentration of ∼100 mM.

The nonbonded van der Waals interaction contains an excluded-volume term and a data-driven ME term

3 $$U_{\mathrm{vdW}}=U_{\mathrm{core}}+U_{\mathrm{ME}}$$

The excluded-volume term prevents bead overlap and was defined as

$$U_{\mathrm{core}}\left(r_{\mathrm{ij}}\right)=\{\begin{array}{cc} 4\epsilon \left[{\left(\frac{c_{e}\sigma_{s_{i}s_{j}}}{r_{\mathrm{ij}}}\right)}^{12}-{\left(\frac{c_{e}\sigma_{s_{i}s_{j}}}{r_{\mathrm{ij}}}\right)}^{6}\right]+\epsilon , & r_{\mathrm{ij}}\leq 2^{1/6}c_{e}\sigma_{s_{i}s_{j}}\\ 0, & r_{\mathrm{ij}}>2^{1/6}c_{e}\sigma_{s_{i}s_{j}} \end{array}$$ 4

Here ϵ = 0.2 kcal/mol. The residue-pair diameter was calculated from residue-type-specific bead radii

5 $$\sigma_{s_{i}s_{j}}=\left(\sigma_{s_{i}}+\sigma_{s_{j}}\right)/2$$

where the residue-type parameters σ_*s*_*i*__ were taken from the literature.^28^

The ME term was defined as

6 $$U_{\mathrm{ME}}\left(r_{\mathrm{ij}}\right)=\frac{\alpha_{s_{i}s_{j}}}{2}\left[1-\tanh\left(\frac{r_{\mathrm{ij}}-c_{c}\sigma_{s_{i}s_{j}}}{\delta_{c}}\right)\right]$$

Here, α_*s*_*i*_ *s*_*j*__ is the optimized interaction coefficient for the residue-type pair *s*_*i*_,*s*_*j*_, *c*_c_σ_*s*_*i*_ *s*_*j*__ sets the characteristic contact distance, and δ_c_ determines the smoothness of the contact transition. We used δ_c_ = 1 Å for all residue-type pairs. The parameters *c*_e_ and *c*_c_ tune the excluded-volume radius and attractive capture radius, respectively. For Qch, Mid, and Flx, the parameter pairs (*c*_e_,*c*_c_) were set to (1.0,1.55), (0.82,1.50), and (0.60,1.37), respectively, based on the parameter scan shown in Figure S62.

### Molecular Dynamics Simulations

#### AA Simulations

The initial FUS-LCD structure was generated using AlphaFold 3.^79^ Because FUS-LCD is intrinsically disordered, this structure was used only as a starting coordinate set rather than as a folded reference state. To generate a diverse set of initial conformations, the AlphaFold-derived model was first subjected to a 1 μs AA molecular dynamics simulation. From the last 800 ns of this trajectory, 16 representative conformations were selected based on pairwise root-mean-square deviation (RMSD). Each selected conformation was then used as an independent starting structure for production simulations. For each starting conformation, four simulations with different initial velocities and random seeds were performed, giving 64 production trajectories in total. Each production trajectory was run for at least 2 μs.

AA simulations were performed using GROMACS 2024.5 ^80,81^ with the amber99sbws-stq protein force field^68,82–85^ and the TIP4*P*/2005 water model.^86^ Hydrogen atoms in the input structures were regenerated using gmx pdb2gmx with the -ignh option. Each system was placed in an octahedral simulation box with an initial box-vector length of 130 Å and solvated with TIP4*P*/2005 water. Sodium and chloride ions were added to neutralize the system and to obtain a NaCl concentration of 0.1 M.

Energy minimization was performed using the steepest-descent algorithm for a maximum of 50,000 steps, with convergence defined by a maximum force below 23.9 kcal/(mol·Å). The minimized systems were equilibrated in two stages. First, constant-number, constant-volume, constant-temperature (NVT) equilibration was performed for 100 ps at 300 K using the V-rescale thermostat.^87^ Second, constant-number, constant-pressure, constant-temperature (NPT) equilibration was performed for 100 ps at 300 K and 1 bar using the V-rescale thermostat and the Parrinello–Rahman barostat^88^ with isotropic pressure coupling.

Production simulations were performed in the NPT ensemble at 300 K and 1 bar with a 2 fs integration time step. Bonds involving hydrogen atoms were constrained using the LINCS algorithm.^89,90^ Temperature coupling was applied separately to the protein and nonprotein groups using the V-rescale thermostat with a coupling time constant of 100 fs. Pressure was maintained using the Parrinello–Rahman barostat with a coupling time constant of 2 ps and a reference pressure of 1 bar. Long-range electrostatic interactions were treated using particle-mesh Ewald summation^91^ with a real-space cutoff of 10 Å, a Fourier grid spacing of 1.6 Å, and fourth-order interpolation. The van der Waals cutoff was set to 10 Å, and analytical dispersion corrections were applied to the energy and pressure. Coordinates, energies, and log files were saved every 10 ps during production. After the simulations, the minimum distance between periodic images was calculated for each system. The smallest value was approximately 24 Å, substantially larger than the cutoff distances used for the nonbonded interactions.

#### ME Optimization of CG Interactions

AA trajectories were used to calculate the ensemble-averaged intrachain contact probability map, ⟨*p*_ij_^AA^⟩, between C_α_ atoms. The contact probability for a residue pair *i*,*j* was defined as

7 $$p_{\mathrm{ij}}=\frac{1}{2}\left[1-\tanh\left(\frac{r_{\mathrm{ij}}-c_{c}\sigma_{s_{i}s_{j}}}{\delta_{c}}\right)\right]$$

with *c*_c_ = 1.5 and the corresponding residue-pair bead diameter σ_*s*_*i*_ *s*_*j*__. The local contact probabilities with |*i*–*j*| < 4 were set to 0 for all intrachain contact maps. The resulting AA contact map served as the target for ME optimization.

Unless otherwise stated, all CG simulations described below were performed using LAMMPS (9Oct2020)^92,93^ with a time step of 10 fs. For each CG model, all parameters were fixed during ME optimization except the residue-type interaction coefficients α_*s*_*i*_ *s*_*j*__. These coefficients were initialized to zero. At each optimization iteration, NVT Langevin dynamics simulations were performed for isolated chains in a sufficiently large simulation box. The damping time constant was 1 ps. To improve sampling efficiency, 200 simulations were run in parallel from distinct random initial conformations. In each simulation, the first 10 ns were used for simulated annealing from 1000 to 300 K, the following 10 ns were used for relaxation at 300 K, and the final 50 ns were used for sampling at 300 K. Frames were saved every 100 ps, yielding 10^5^ conformations for contact-map estimation at each iteration.

To update the α_*s*_*i*_ *s*_*j*__ values according to the ME principle, contact probabilities *p*_*ij*_^CG^ of the CG model were calculated using the corresponding *c*_c_ value. The residue-level contact maps with elements ⟨*p*_*ij*_^AA^⟩ and *p*_*ij*_^CG^ were converted to residue-type-level maps with elements ⟨*p*_*s*_*i*_ *s*_*j*__^AA^⟩ and *p*_*s*_*i*_ *s*_*j*__^CG^

8 $$p_{s_{i}s_{j}}=\frac{1}{N_{s_{i}s_{j}}}\sum_{i\in s_{i}\;,j\in s_{j}\;}p_{\mathrm{ij}}$$

Here, *N*_*s*_*i*_ *s*_*j*__ is the number of residue pairs whose residue types are *s*_*i*_ and *s*_*j*_. After indexing the 20 residue types, the upper triangles of α_*s*_*i*_ *s*_*j*__, ⟨*p*_*s*_*isj*__^AA^⟩, and *p*_*s*_*i*_ *s*_*j*__^CG^ are then flattened into column vectors $A_{\mu }$ , $\left\langle P_{\mu }^{\mathrm{AA}}\right\rangle$ , and $P_{\mu }^{\mathrm{CG}}$ , with μ = 20­(*s*_*i*_ – 1) – *s*_*i*_(*s*_*i*_ – 1)/2 + *s*_*j*_. The α_*s*_*i*_ *s*_*j*__ values are updated by^46–49^

9 $$\Delta \left[A_{\mu }\right]={\left[\left\langle P_{\mu }^{\mathrm{CG}}P_{\nu }^{\mathrm{CG}}\right\rangle -\left\langle P_{\mu }^{\mathrm{CG}}\right\rangle \left\langle P_{\nu }^{\mathrm{CG}}\right\rangle \right]}^{-1}\left[\left\langle P_{\nu }^{\mathrm{CG}}\right\rangle -\left\langle P_{\nu }^{\mathrm{AA}}\right\rangle \right]$$

Here, [·] denotes the organization of elements into a matrix or column vector, and the repeated index ν is summed over all flattened residue-type pairs. After each update of α_*s*_*i*_ *s*_*j*__, the simulation and contact-map calculation are repeated. The procedure is iterated 20 times, after which the CG contact map reproduces the AA contact map well. The final set of α_*s*_*i*_ *s*_*j*__ values is used as the optimized interaction parameter set.

This optimization was performed independently for each (*c*_e_,*c*_c_) parameter pair. Therefore, Qch, Mid, and Flx have distinct *R*_g_ distributions but retain AA-informed residue-type contact propensities.

#### Single-Chain CG Simulations

After ME optimization, each CG model was simulated as an isolated chain at 300 K to characterize its single-chain ensemble. The simulation setup was identical to that used for the ME optimization described above.

#### Double-Chain Umbrella-Sampling Simulations

NVT Langevin dynamics simulations of two chains were performed in a sufficiently large simulation box with a damping time constant of 1 ps. A harmonic biasing potential was applied to the center-of-mass separation *d*_com_ between the two chains

10 $$U_{\mathrm{bs}}=\frac{1}{2}K_{\mathrm{bs}}{\left(d_{\mathrm{com}}-d_{\mathrm{com}}^{0}\right)}^{2}$$

*K*_bs_ was set to 0.1 kcal/(mol·Å^2^), and the window centers *d*_com_^0^ ranged from 0 to 166.6 Å in increments of 3.4 Å.^24^ For each model, three independent umbrella-sampling data sets were generated from different initial configurations, with each data set containing all *d*_com_^0^ windows. Each window was simulated for 10 μs, and configurations from the final 8 μs were saved every 100 ps for analysis. The independent data sets were used to assess convergence.

#### Multichain Slab Simulations

For thermodynamic sampling, we used a slab simulation setup. 100 FUS-LCD chains with random initial conformations were placed in a simulation box. An NPT simulation at 300 K and 100 bar was performed for 100 ps to generate a dense initial configuration. The final frame was then transferred to an elongated box of size 150 × 150 × 500 Å^3^. NVT Langevin dynamics simulations were performed with a damping time constant of 1 ns. The elongated box stabilizes a dense slab with two dense–dilute interfaces normal to the *z* direction. Each slab simulation was run for 1500 ns; the first 500 ns were discarded as relaxation, and the final 1 μs were used for analysis. Frames were saved every 100 ps. Slab simulations were performed for Qch, Mid, and Flx at 300, 350, 400, and 450 K.

#### Spherical Condensate and Condensation-Kinetics Simulations

50 chains with random conformations were placed in a cubic simulation box with side length 500 Å. Initial configurations were generated to minimize interchain contacts. NVT Langevin dynamics simulations were then performed using the same temperature and damping time constant as in the slab simulations. Each trajectory was run for 1 μs with a dump interval of 100 ps. For each of Qch, Mid, and Flx, 20 independent trajectories were generated using different initial configurations. The final 700 ns of each equilibrated trajectory were used to calculate the equilibrium properties of the spherical condensates. The full trajectories were used to analyze condensation kinetics.

### Analysis Methods

#### Single-Chain Structural Descriptors

For a chain with *n* residues, the center of mass was first translated to the origin. The coordinate matrix and mass vector were written as

11 $$R=\left(\begin{array}{llll} x_{1} & x_{2} & ... & x_{n}\\ y_{1} & y_{2} & ... & y_{n}\\ z_{1} & z_{2} & ... & z_{n} \end{array}\right)$$

and the mass vector is

12 $$m=\left(\begin{array}{l} m_{1}\\ m_{2}\\ \vdots \\ m_{n} \end{array}\right)$$

Here, (*x*_*i*_, *y*_*i*_, *z*_*i*_) and *m*_*i*_ are the Cartesian coordinates and mass of the *i*-th residue. The covariance tensor is

13 $$C=\frac{R\;\mathrm{diag}\left(m\right)\;R^{T}}{\mathrm{tr}\left[\mathrm{diag}\left(m\right)\right]}$$

The *i*-th eigenvalue of *C* is denoted as λ_*i*_, and λ̅ = (λ_1_ + λ_2_ + λ_3_)/3. The inertial principal-axis length is

14 $$R_{i}=\sqrt{\lambda_{i}}$$

the radius of gyration is

15 $$R_{g}=\sqrt{\mathrm{tr}C}=\sqrt{\sum_{i=1}^{3}\lambda_{i}}$$

the asphericity is

16 $$\Delta =\frac{3}{2}\frac{\sum_{i=1}^{3}{\left(\lambda_{i}-\mathrm{\lambda ̅}\right)}^{2}}{{\left(\mathrm{tr}C\right)}^{2}}$$

and the shape parameter is

17 $$S=27\frac{\prod_{i=1}^{3}\left(\lambda_{i}-\mathrm{\lambda ̅}\right)}{{\left(\mathrm{tr}C\right)}^{3}}$$

The asphericity Δ ranges from 0 to 1, with larger values indicating stronger deviation from a spherical shape. Positive and negative values of *S* correspond to predominantly prolate and oblate conformations, respectively.

#### Conformational PCA

PCA was performed using an intrinsic distance-based descriptor. For each conformation, four mutually distant residues were selected as reference points. The distances among these reference residues and the distances from each reference residue to all other residues form a compact 4 × *n* descriptor. This representation removes translational and rotational degrees of freedom while retaining the information needed to reconstruct the remaining pairwise distances through the Cayley–Menger relation. PCA was then performed on the pooled conformational descriptors, and each conformation was projected onto the first two principal coordinates, denoted ζ_1_ and ζ_2_. Additional details are provided in the Supporting Methods.

#### Autocorrelation Functions and Relaxation Times

For a time-dependent quantity *X*(*t*), the normalized autocorrelation function was calculated as

18 $$f_{X}^{\mathrm{ac}}\left(\Delta t\right)=\frac{{\left\langle \left[X\left(t+\Delta t\right)-\langle X\rangle_{t}\right]\left[X\left(t\right)-\langle X\rangle_{t}\right]\right\rangle }_{t}}{{\left\langle {\left[X\left(t\right)-\langle X\rangle_{t}\right]}^{2}\right\rangle }_{t}}$$

Here, ⟨·⟩_t_ denotes a time average. For residue-pair distance relaxation, *X*(*t*) = *d*_*ij*_(*t*). The relaxation time τ­(*d*_*ij*_) was defined as the lag time Δ*t* at which *f*_*X*_^ac^(Δ*t*) = 1/*e*, where *e* is Euler’s number, the base of the natural logarithm.

#### Mean-Squared Displacement

Diffusive motion was quantified by the mean-squared displacement (MSD),

19 $$\left\langle \Delta r^{2}\left(\Delta t\right)\right\rangle ={\left\langle {\left|r\left(t+\Delta t\right)-r\left(t\right)\right|}^{2}\right\rangle }_{t}$$

Here, *r*(*t*) is the relevant residue or chain center-of-mass position vector. For isolated chains, residue-level MSDs were calculated relative to the chain center of mass to measure internal chain mobility. For condensates, center-of-mass MSDs of chains were used to quantify translational mobility within the dense phase.

#### Density Profiles and Coexistence Curves

For slab simulations, coordinates were shifted along the *z* direction so that the condensate center of mass was located at *z* = 0. The density profile along *z* was fitted by

20 $$\rho \left(z\right)=\rho_{L}+\frac{\rho_{H}-\rho_{L}}{2}\left[\tanh\left(\frac{z+z_{s}}{\delta_{s}}\right)-\tanh\left(\frac{z-z_{s}}{\delta_{s}}\right)\right]$$

where ρ_H_ and ρ_L_ are the dense- and dilute-phase densities, *z*_s_ is the half-width of the dense slab, and δ_s_ characterizes the interfacial width.^94,95^ For chain-number density profiles, *z*_c_ denotes the *z* coordinate of a chain center of mass. The coexistence curve was obtained by fitting the temperature-dependent dense- and dilute-phase densities

21 $$\rho_{H}+\rho_{L}=2\rho_{c}+A\left(T_{c}-T\right)$$

and

22 $$\rho_{H}-\rho_{L}=B{\left(T_{c}-T\right)}^{\beta }$$

Here, *T*_c_ is the critical temperature, ρ_c_ is the critical density, *A* and *B* are fitting constants, and β is the critical exponent used in the fit.^94–96^

The condensate interior was defined as

23 $$-z_{s}+\delta_{s}<z<z_{s}-\delta_{s}$$

The condensate surface was defined as

24 $$-z_{s}-\delta_{s}<z<-z_{s}+\delta_{s}\cup z_{s}-\delta_{s}<z<z_{s}+\delta_{s}$$

For position-dependent analyses focused on the condensate and its interface, the normalized coordinate was defined as

25 $$z_{\mathrm{norm}}=\frac{z}{z_{s}+\delta_{s}}$$

For a spherical condensate, coordinates were shifted so that the condensate center of mass was located at *r* = 0. The density profile along *r* was fitted by

26 $$\rho \left(r\right)=\rho_{L}+\frac{\rho_{H}-\rho_{L}}{2}\left[1-\tanh\left(\frac{r-r_{s}}{\delta_{s}}\right)\right]$$

where ρ_H_, ρ_L_, *r*_s_, and δ_s_ have meanings analogous to those defined above. For position-dependent analyses, the normalized coordinate was defined as

27 $$r_{\mathrm{norm}}=\frac{r}{r_{s}+\delta_{s}}$$

#### Surface Tension

The surface tension was calculated from the anisotropy of the pressure tensor,

28 $$\Gamma =\frac{L_{z}}{2}\left[P_{\mathrm{zz}}-\frac{P_{\mathrm{xx}}+P_{\mathrm{yy}}}{2}\right]$$

where *P*_*xx*_, *P*_*yy*_, and *P*_*zz*_ are diagonal components of the pressure tensor, *L*_*z*_ is the box length along the slab-normal direction, and the factor of 2 accounts for the two dense–dilute interfaces.^31,36,97^ Because instantaneous pressure-tensor estimates fluctuate strongly, surface-tension statistics were evaluated using window-averaged values, as described in the Supporting Methods.

#### Second Virial Coefficient

The sampled *d*_com_ data from each *d*_com_^0^ window were analyzed using the weighted histogram analysis method (WHAM)^98^ to obtain the PMF *F*(*d*_com_). The PMF was then converted into the normalized radial distribution function *g*(*d*_com_)­

29 $$g\left(d_{\mathrm{com}}\right)=\lim_{d_{\mathrm{ref}}\;\to \infty }\frac{d_{\mathrm{ref}}^{2}}{d_{\mathrm{com}}^{2}}\exp\left(-\frac{F\left(d_{\mathrm{com}}\right)-F\left(d_{\mathrm{ref}}\right)}{k_{B}T}\right)$$

Here, *d*_ref_ denotes a reference center-of-mass separation in the asymptotic noninteracting regime, *k*_B_ is the Boltzmann constant and *T* is the temperature. The second virial coefficient *B*_22_ was then calculated as^24^

30 $$B_{22}=\int_{0}^{\infty }\left[1-g\left(d_{\mathrm{com}}\right)\right]2\pi d_{\mathrm{com}}^{2}dd_{\mathrm{com}}$$

#### Chain Orientation and Orientational Order

Each FUS-LCD chain in the condensate was assigned an orientation vector **χ**_*I*_ along its first principal axis. The sign of **χ**_*I*_ was chosen so that the vector points from the N-terminal side to the C-terminal side. The orientation angle relative to the slab-normal direction was

31 $$\theta_{I}=\arccos\left(\chi_{I}\cdot e_{z}\right)$$

where *e*_*z*_ is the unit vector along the *z* axis. For two contacted chains *I* and *J*, the relative orientation angle was

32 $$\phi_{\mathrm{IJ}}=\arccos\left(\chi_{I}\cdot \chi_{J}\right)$$

With the angles expressed in degrees, the random-orientation reference distributions $f\left(\theta \right)=\frac{\pi }{360}\sin\left(\frac{\pi \theta }{180}\right)$ and $f\left(\phi \right)=\frac{\pi }{360}\sin\left(\frac{\pi \phi }{180}\right)$ were used to quantify orientational order.

The Fisher–Rao distance between two probability distributions *f*(*x*) and *g*(*x*) was calculated as

33 $$d_{\mathrm{FR}}\left(f\left(x\right),g\left(x\right)\right)=2\arccos\left[\int \sqrt{f\left(x\right)g\left(x\right)}dx\right]$$

A larger *d*_FR_ indicates a stronger statistical deviation from the reference distribution.

For a spherical condensate, the analysis was performed analogously by replacing *e*_*z*_ with the local radial unit vector *e*_*r*_.

#### Chain-Contact Networks

Each condensate was represented as a chain-contact network. Each chain was treated as a node. For two chains *I* and *J*, the chain-pair contact sum was

34 $$p_{\mathrm{IJ}}=\sum_{i\in I,j\in J}p_{\mathrm{ij}}$$

Two chains were considered connected if *p*_*IJ*_ > 1. This threshold was chosen from the distribution of the chain-pair contact sum, where *p*_*IJ*_ = 1 lies near a major inflection point and close to the median of the distribution (Figures S63 and S64).

For a network with *N* nodes and *N* ^edge^ edges, the degree *N*_*I*_^deg^ of node *I* was defined as the number of nodes connected to it. The betweenness centrality of node *I* was calculated as

35 $$g_{I}^{\mathrm{bc}}=\sum_{\begin{array}{l} J<K\\ J\neq I,K\neq I \end{array}}\frac{N_{J\to I\to K}^{\mathrm{path}}}{N_{J\to K}^{\mathrm{path}}}$$

where *N*_*J*→*I*→*K*_^path^ is the number of shortest paths from node *J* to node *K* that pass through node *I*, and *N*_*J*→*K*_^path^ is the total number of shortest paths from node *J* to node *K*.^37^

The small-world coefficient was calculated as

36 $$c_{\mathrm{swn}}=\frac{\frac{1}{N}\sum_{I=1}^{N}\frac{2N_{I}^{\mathrm{edge},\mathrm{nei}}}{N_{I}^{\deg}\left(N_{I}^{\deg}-1\right)}}{\frac{1}{N\left(N-1\right)}\sum_{I\neq J}L_{\mathrm{IJ}}^{\mathrm{path}}}\cdot \frac{\frac{\ln N-\gamma_{\mathrm{eul}}}{\ln\left(2N^{\mathrm{edge}}/N\right)}+\frac{1}{2}}{\frac{2N^{\mathrm{edge}}}{N\left(N-1\right)}}$$

Here, *N*_*I*_^edge, nei^ is the number of edges among the neighbors of node *I*, *L*_*IJ*_^path^ is the shortest-path length between nodes *I* and *J*, and γ_eul_ is Euler’s constant.^99–102^

#### Apparent Diffusion Coefficients in Condensates

The center-of-mass diffusion of each chain in the condensate was quantified by the MSD. Because the slab geometry is anisotropic, diffusion was analyzed separately in the interfacial plane and along the slab-normal direction. The lag-time-dependent apparent diffusion coefficient was defined as^54^

37 $$D\left(\Delta t\right)=\frac{\left\langle \Delta r^{2}\left(\Delta t\right)\right\rangle }{2n_{r}\Delta t}$$

where *n*_*r*_ is the number of spatial dimensions included in the displacement vector *r*. For in-plane diffusion, *n*_*r*_ = 2; for slab-normal diffusion, *n*_*r*_ = 1.

#### Contact-Lifetime Statistics

The lifetimes of interchain contact states were calculated from the binary edge trajectories of the chain-contact network. A contact lifetime τ_1_ is the continuous duration for which a pair of chains remains connected, whereas a noncontact lifetime τ_0_ is the continuous duration for which the same pair remains disconnected before contact reforms. Because these lifetime distributions are strongly skewed, their average levels were summarized in log space. For a lifetime τ, the geometric mean was

38 $$\langle \tau \rangle_{\ln}=\exp\left(\langle \ln\tau \rangle \right)$$

The lower and upper log-space dispersions were calculated separately for values below and above the geometric mean, as described in the Supporting Methods.

#### Network-Mode Relaxation and Viscoelastic Moduli

Viscoelastic relaxation was estimated using a graph-based Rouse–Zimm description.^56–60^ For each chain-contact network, we constructed a weighted graph Laplacian

$$Z_{\mathrm{IJ}}=\{\begin{array}{l} \sum_{K\neq I}p_{\mathrm{IK}},,I=J\\ -p_{\mathrm{IJ}},,I\neq J,p_{\mathrm{IJ}}>1\\ 0,,I\neq J,p_{\mathrm{IJ}}\leq 1 \end{array}$$ 39

Here, *p*_*IJ*_ is the chain-pair contact sum defined above. The zero eigenvalue corresponding to global translation was excluded, leaving *N* – 1 relaxation modes. The relaxation time of mode *n* was

40 $$\tau_{n}=\frac{a}{6k_{B}T\lambda_{n}},,1\leq n\leq N-1$$

where λ_*n*_ is the *n*-th nonzero eigenvalue of *Z*, *k*_B_ is the Boltzmann constant, *T* is the temperature, and *a* is a proportionality constant. Because *a* was not calibrated against experiments, we set *a* = 1 and report relaxation times in reduced units.

The storage and loss moduli were estimated from the relaxation spectrum as

41 $$G^{\prime }\left(\omega \right)=\frac{bk_{B}T}{N}\sum_{n=1}^{N-1}\frac{\omega^{2}\tau_{n}^{2}}{1+\omega^{2}\tau_{n}^{2}}$$

and

42 $$G^{\prime \prime }\left(\omega \right)=\frac{bk_{B}T}{N}\sum_{n=1}^{N-1}\frac{\omega \tau_{n}}{1+\omega^{2}\tau_{n}^{2}}$$

Here, ω is the angular frequency, and *b* is a proportionality constant that sets the modulus scale. Because *b* was not calibrated experimentally, the moduli are reported in reduced units.

In the low-frequency limit

43 $$G^{\prime \prime }\left(\omega \right)∼g_{v0}\omega$$

and

44 $$G^{\prime }\left(\omega \right)∼g_{e0}g_{v0}^{2}\omega^{2}$$

where *g*_v0_ is the zero-shear viscosity and *g*_e0_ is the steady-state compliance. For the mode spectrum above

45 $$g_{v0}=\frac{bk_{B}T}{N}\sum_{n=1}^{N-1}\tau_{n}$$

and

46 $$g_{e0}=\frac{N}{bk_{B}T}\frac{\sum_{n=1}^{N-1}\tau_{n}^{2}}{{\left(\sum_{n=1}^{N-1}\tau_{n}\right)}^{2}}$$

#### Cluster Analysis of Condensation Kinetics

Condensation kinetics were analyzed using the time-dependent number of clusters, *N*_clst_(*t*), and the number of isolated chains, *N*_iso_(*t*). Chains belonging to the same connected component of the interchain contact network were assigned to the same cluster.

The time dependence of *N*_clst_ was fitted using either a single-exponential form

47 $$N_{\mathrm{clst}}\left(t\right)=\left(N-1\right)\exp\left(-k_{0}t\right)+1$$

or a double-exponential form

48 $$N_{\mathrm{clst}}\left(t\right)=\left(N-1\right)\left[w\exp\left(-k_{1}t\right)+\left(1-w\right)\exp\left(-k_{2}t\right)\right]+1$$

Here, *N* is the total number of chains, *w* is the fitted weight of one exponential component, and *k*_0_, *k*_1_, and *k*_2_ are fitted rate constants. The number of isolated chains was fitted analogously

49 $$N_{\mathrm{iso}}\left(t\right)=N\exp\left(-k_{0}^{\prime }t\right)$$

or

50 $$N_{\mathrm{iso}}\left(t\right)=N\left[w\exp\left(-k_{1}^{\prime }t\right)+\left(1-w\right)\exp\left(-k_{2}^{\prime }t\right)\right]$$

Here, *k*_0_^′^, *k*_1_^′^, and *k*_2_^′^ are the corresponding fitted rate constants for isolated-chain absorption. The cluster-growth rate was calculated from the fitted curve

51 $$v_{\mathrm{clst}}\left(t\right)=-\frac{dN_{\mathrm{clst}}\left(t\right)}{dt}$$

Because *N*_clst_ decreases by one for any binary merger regardless of cluster size, we introduced a cluster-size amplitude to distinguish small-cluster nucleation from large-cluster coalescence. For a system with *N*_clst_ clusters, where *N*_*i*_ is the number of chains in cluster *i*, the cluster-size amplitude was defined as

52 $$A_{\mathrm{clst}}=\sqrt{\sum_{i=1}^{N_{\mathrm{clst}}}N_{i}^{2}}$$

For a merger between two clusters of sizes *N*_1_ and *N*_2_, the change in *A*_clst_^2^ is

53 $$\Delta A_{\mathrm{clst}}^{2}={\left(N_{1}+N_{2}\right)}^{2}-\left(N_{1}^{2}+N_{2}^{2}\right)=2N_{1}N_{2}$$

Thus, mergers involving larger clusters produce larger changes in *A*_clst_ than mergers involving small clusters.

#### Intrachain and Interchain Contact Formation during Condensation

The progress of contact formation during condensation was quantified using the normalized variable

54 $$Q\left(t\right)=\frac{P\left(t\right)-P\left(0\right)}{P\left(\infty \right)-P\left(0\right)}$$

Here, *P*(0) and *P*(∞) are the values in the initial dispersed state and final condensed state, respectively, and

55 $$P=\sum_{i,j}p_{\mathrm{ij}}$$

was computed separately for intrachain and interchain contacts. For the intrachain case, *p*_*ij*_ with |*i*–*j*| < 4 was still discarded. The resulting curves were fitted by

56 $$Q_{\mathrm{intra}/\mathrm{inter}}\left(t\right)=\sum_{i=1}^{n_{e}}C_{i}\left[1-\exp\left(-k_{i}t\right)\right]$$

where *C*_*i*_ and *k*_*i*_ are fitted amplitudes and rate constants, *n*_e_ = 3 for intrachain contacts, and *n*_e_ = 2 for interchain contacts. The corresponding formation rates, *v*_intra_ and *v*_inter_, were calculated as the time derivatives of the fitted *Q*_intra_(*t*) and *Q*_inter_(*t*) curves.

### Statistical Analysis

Unless otherwise stated, averages over simulation trajectories are reported as means, and error bars denote standard deviations over the corresponding statistical samples. For slab simulations, statistics were collected from the production portion of each trajectory. For condensation kinetics, statistics were calculated over 20 independent trajectories for each model. For strongly skewed contact-lifetime distributions, geometric means and asymmetric log-space dispersions were used instead of arithmetic means and standard deviations.

## Supplementary Material



## Data Availability

All files and scripts for running AA simulations, ME optimization, HPS-model simulations, slab simulations, double-chain simulations, and condensation-kinetics simulations, as well as for data processing, analysis, and figure generation, are available at https://github.com/CiboFeng/ConformationalFluctuation. Large raw trajectory files are available from the corresponding author upon reasonable request because of storage-size limitations.

## Notes

Supporting methods for PCA calculations; comparisons of the model and temperature dependence of the mean, standard deviation, and coefficient of variation; treatment of error bars for strongly skewed distributions; numerical comparison of key physical quantities; and Supporting figures of model validation, phase behavior, condensate organization, molecular dynamics, and condensation kinetics (PDF)
