# Supplementary material for: Single-Chain Conformational Fluctuation Regulates Phase Separation of Intrinsically Disordered Proteins
Source: JACS Au. 2026 Sep 24;6(10):5765–84. doi: 10.1021/jacsau.6c01044 (PMC13714856; doi:10.1021/jacsau.6c01044)
Supplement: Supplementary file 1 [file au6c01044_si_001.pdf]

Supporting Information for "Single-chain conformational fluctuation regulates phase separation of intrinsically disordered proteins"

Cibo Feng[1], Shiqinrui Xu[1], Haibin Su[2], and Xiakun Chu[1,3,4*]

[1]*Advanced Materials Thrust, Function Hub*
The Hong Kong University of Science and Technology (Guangzhou)
Guangzhou, 511453, China

[2]*Department of Chemistry*
The Hong Kong University of Science and Technology
Clear Water Bay, Kowloon, Hong Kong SAR 999077, China

[3]*Guangzhou Municipal Key Laboratory of Materials Informatics*
The Hong Kong University of Science and Technology (Guangzhou)
Guangzhou, 511453, China

[4]*Division of Life Science*
The Hong Kong University of Science and Technology
Clear Water Bay, Kowloon, Hong Kong SAR 999077, China

[*]*Corresponding author: xiakunchu@hkust-gz.edu.cn*

1 Supplementary Methods

For conformational principal component analysis (PCA), a descriptor should be complete, intrinsic, and computationally efficient. Cartesian coordinates are complete but not intrinsic, because translations and rotations introduce extrinsic degrees of freedom that complicate alignment. A distance matrix is complete and intrinsic but inefficient because it contains redundant entries and is memory-intensive. This redundancy can be reduced using the Cayley–Menger determinant. Specifically, the distances among six points satisfy the zero Cayley–Menger determinant:

$$\begin{vmatrix} d_{11}^2 & d_{12}^2 & d_{13}^2 & d_{14}^2 & d_{1i}^2 & d_{1j}^2 & 1 \\ d_{21}^2 & d_{22}^2 & d_{23}^2 & d_{24}^2 & d_{2i}^2 & d_{2j}^2 & 1 \\ d_{31}^2 & d_{32}^2 & d_{33}^2 & d_{34}^2 & d_{3i}^2 & d_{3j}^2 & 1 \\ d_{41}^2 & d_{42}^2 & d_{43}^2 & d_{44}^2 & d_{4i}^2 & d_{4j}^2 & 1 \\ d_{i1}^2 & d_{i2}^2 & d_{i3}^2 & d_{i4}^2 & d_{ii}^2 & d_{ij}^2 & 1 \\ d_{j1}^2 & d_{j2}^2 & d_{j3}^2 & d_{j4}^2 & d_{ji}^2 & d_{jj}^2 & 1 \\ 1 & 1 & 1 & 1 & 1 & 1 & 0 \end{vmatrix} = 0. \tag{1}$$

Here, the six points are labeled as two target points, i and j, and four reference points, 1, 2, 3, and 4. The distance d_{ij} between the two target points can therefore be derived if the other distances are known. The equation above is a quadratic equation in d_{ij} and has a repeated root, yielding

$$d_{ij} = \sqrt{-\frac{\begin{vmatrix} d_{11}^2 & d_{12}^2 & d_{13}^2 & d_{14}^2 & d_{1j}^2 & 1 \\ d_{21}^2 & d_{22}^2 & d_{23}^2 & d_{24}^2 & d_{2j}^2 & 1 \\ d_{31}^2 & d_{32}^2 & d_{33}^2 & d_{34}^2 & d_{3j}^2 & 1 \\ d_{41}^2 & d_{42}^2 & d_{43}^2 & d_{44}^2 & d_{4j}^2 & 1 \\ d_{i1}^2 & d_{i2}^2 & d_{i3}^2 & d_{i4}^2 & 0 & 1 \\ 1 & 1 & 1 & 1 & 1 & 0 \end{vmatrix}}{\begin{vmatrix} d_{11}^2 & d_{12}^2 & d_{13}^2 & d_{14}^2 & 1 \\ d_{21}^2 & d_{22}^2 & d_{23}^2 & d_{24}^2 & 1 \\ d_{31}^2 & d_{32}^2 & d_{33}^2 & d_{34}^2 & 1 \\ d_{41}^2 & d_{42}^2 & d_{43}^2 & d_{44}^2 & 1 \\ 1 & 1 & 1 & 1 & 0 \end{vmatrix}}}. \tag{2}$$

Thus, if the distances among the four reference points and the distances between those reference points and all other points are known, i.e., if any four rows or columns of the distance matrix are retained, the distances among all other points can be derived. The original $n \times n$ distance matrix can therefore be reduced to a $4 \times n$ matrix, with all other elements treated as redundant. This construction requires that the four reference points are not coplanar. In practice, choosing four mutually distant reference points is sufficient to reduce numerical error. A $4 \times n$ matrix is still redundant because the system has only $3n - 6$ intrinsic degrees of freedom. To reduce it further, the Cayley–Menger relation can also be written for five points, corresponding to three reference points. However, in that case the quadratic equation for d_{ij} has two distinct roots, corresponding to two mirror-symmetric configurations, making reconstruction less convenient. Because further reduction requires more advanced algorithms and the $4 \times n$ representation is already sufficiently efficient, we did not reduce it further.

To compare how different quantities vary across models and temperatures (i.e., across conditions), we examine the relative deviation of each quantity from its mean over all conditions. As an example, we consider $\langle R_\mathrm{g} \rangle$, std.$(R_\mathrm{g})$, and cv.$(R_\mathrm{g})$. The involved values can be arranged into three matrices:

$$\mathbb{X}_1 = \begin{pmatrix} \langle R_\mathrm{g} \rangle_\mathrm{Qch}^{300} & \langle R_\mathrm{g} \rangle_\mathrm{Qch}^{350} & \langle R_\mathrm{g} \rangle_\mathrm{Qch}^{400} & \langle R_\mathrm{g} \rangle_\mathrm{Qch}^{450} \\ \langle R_\mathrm{g} \rangle_\mathrm{Mid}^{300} & \langle R_\mathrm{g} \rangle_\mathrm{Mid}^{350} & \langle R_\mathrm{g} \rangle_\mathrm{Mid}^{400} & \langle R_\mathrm{g} \rangle_\mathrm{Mid}^{450} \\ \langle R_\mathrm{g} \rangle_\mathrm{Flx}^{300} & \langle R_\mathrm{g} \rangle_\mathrm{Flx}^{350} & \langle R_\mathrm{g} \rangle_\mathrm{Flx}^{400} & \langle R_\mathrm{g} \rangle_\mathrm{Flx}^{450} \end{pmatrix}, \tag{3}$$

$$\mathbb{X}_2 = \begin{pmatrix} \text{std.}(R_\text{g})^{300}_\text{Qch} & \text{std.}(R_\text{g})^{350}_\text{Qch} & \text{std.}(R_\text{g})^{400}_\text{Qch} & \text{std.}(R_\text{g})^{450}_\text{Qch} \\ \text{std.}(R_\text{g})^{300}_\text{Mid} & \text{std.}(R_\text{g})^{350}_\text{Mid} & \text{std.}(R_\text{g})^{400}_\text{Mid} & \text{std.}(R_\text{g})^{450}_\text{Mid} \\ \text{std.}(R_\text{g})^{300}_\text{Flx} & \text{std.}(R_\text{g})^{350}_\text{Flx} & \text{std.}(R_\text{g})^{400}_\text{Flx} & \text{std.}(R_\text{g})^{450}_\text{Flx} \end{pmatrix}, \tag{4}$$

and

$$\mathbb{X}_3 = \begin{pmatrix} \text{cv.}(R_\text{g})^{300}_\text{Qch} & \text{cv.}(R_\text{g})^{350}_\text{Qch} & \text{cv.}(R_\text{g})^{400}_\text{Qch} & \text{cv.}(R_\text{g})^{450}_\text{Qch} \\ \text{cv.}(R_\text{g})^{300}_\text{Mid} & \text{cv.}(R_\text{g})^{350}_\text{Mid} & \text{cv.}(R_\text{g})^{400}_\text{Mid} & \text{cv.}(R_\text{g})^{450}_\text{Mid} \\ \text{cv.}(R_\text{g})^{300}_\text{Flx} & \text{cv.}(R_\text{g})^{350}_\text{Flx} & \text{cv.}(R_\text{g})^{400}_\text{Flx} & \text{cv.}(R_\text{g})^{450}_\text{Flx} \end{pmatrix}. \tag{5}$$

Each matrix corresponds to one quantity, and each entry represents the value under a specific model and temperature. Comparing the variation of a quantity thus essentially reduces to examining the spread of values within its matrix. However, these quantities differ in units and numerical scales. We therefore normalize each matrix by its overall mean and center it:

$$\boldsymbol{x}_i = \mathbb{X}_i / \langle \mathbb{X}_i \rangle_{3\times 4} - 1. \tag{6}$$

Here, $i = 1, 2, 3$, and $\langle\cdot\rangle_{3\times 4}$ denotes the mean value of the 3×4 matrix. We can then focus on comparing the normalized matrices $\boldsymbol{x}_i$.

For strongly skewed distributions, such as the approximately power-law distribution of contact lifetimes, the conventional mean and standard deviation are not very informative. Therefore, the average level and dispersion are defined in log space. The average level is quantified by the geometric mean:

$$\langle\tau\rangle_\text{ln} = \exp(\langle \ln\tau\rangle). \tag{7}$$

Because the distribution is strongly asymmetric around the geometric mean, the dispersion is defined separately below and above the geometric mean. For the lower part,

$$\text{std.}_\text{ln,lo}(\tau) = \exp\left[\sqrt{\langle(\ln\tau_\text{lo} - \langle\ln\tau\rangle)^2\rangle}\right], \tag{8}$$

where τ_lo denotes $\{\tau \mid \tau < \langle\tau\rangle_\text{ln}\}$. For the upper part,

$$\text{std.}_\text{ln,up}(\tau) = \exp\left[\sqrt{\langle(\ln\tau_\text{up} - \langle\ln\tau\rangle)^2\rangle}\right], \tag{9}$$

where τ_up denotes $\{\tau \mid \tau > \langle\tau\rangle_\text{ln}\}$. In the plots, the lower and upper endpoints of the error bar are defined as $\langle\tau\rangle_\text{ln}/\text{std.}_\text{ln,lo}(\tau)$ and $\langle\tau\rangle_\text{ln}\cdot\text{std.}_\text{ln,up}(\tau)$, respectively.

2 Numerical Comparison of Key Physical Quantities

Table S1: Key physical quantities for the three FUS-LCD models. Dashes indicate quantities for which simulations were performed only at 300 K. The listed R_g mean and standard deviation are taken from the plotted 300-K isolated-chain distributions. The contact lifetime is the geometric mean; B_{22} and kinetics entries are mean ± standard deviation over three and 20 replicates, respectively. Values of T_c are model-level coexistence-curve fits and are therefore repeated across temperature columns.

Quantity	Qch				Mid				Flx			
T (K)	300	350	400	450	300	350	400	450	300	350	400	450
$\langle R_g \rangle \pm \mathrm{std}(R_g)$ (Å)	14.932±0.532	—	—	—	14.973±1.081	—	—	—	15.025±2.474	—	—	—
ρ_H (g mL^{-1})	1.069	1.010	0.947	0.878	1.308	1.190	1.061	0.914	1.994	1.681	1.346	0.910
T_c (K)	630.497	630.497	630.497	630.497	524.986	524.986	524.986	524.986	464.140	464.140	464.140	464.140
Γ (mN m^{-1})	0.044	0.038	0.031	0.023	0.031	0.023	0.015	0.008	0.028	0.015	0.009	0.002
$\langle \tau_1 \rangle_{\ln}$ (ns)	0.555	0.529	0.506	0.484	0.516	0.490	0.464	0.434	0.498	0.464	0.424	0.371
B_{22} (Å^3)	$(-1.158\pm0.012)\times10^{15}$	—	—	—	$(-2.420\pm0.072)\times10^{10}$	—	—	—	$(-6.737\pm0.334)\times10^{7}$	—	—	—
$t_{req}(25)$ (ns)	1.920±0.558	—	—	—	2.329±0.479	—	—	—	2.803±0.461	—	—	—
$t_{req}(1)$ (ns)	93.060±36.015	—	—	—	85.285±35.834	—	—	—	87.940±31.810	—	—	—

3 Supplementary Figures

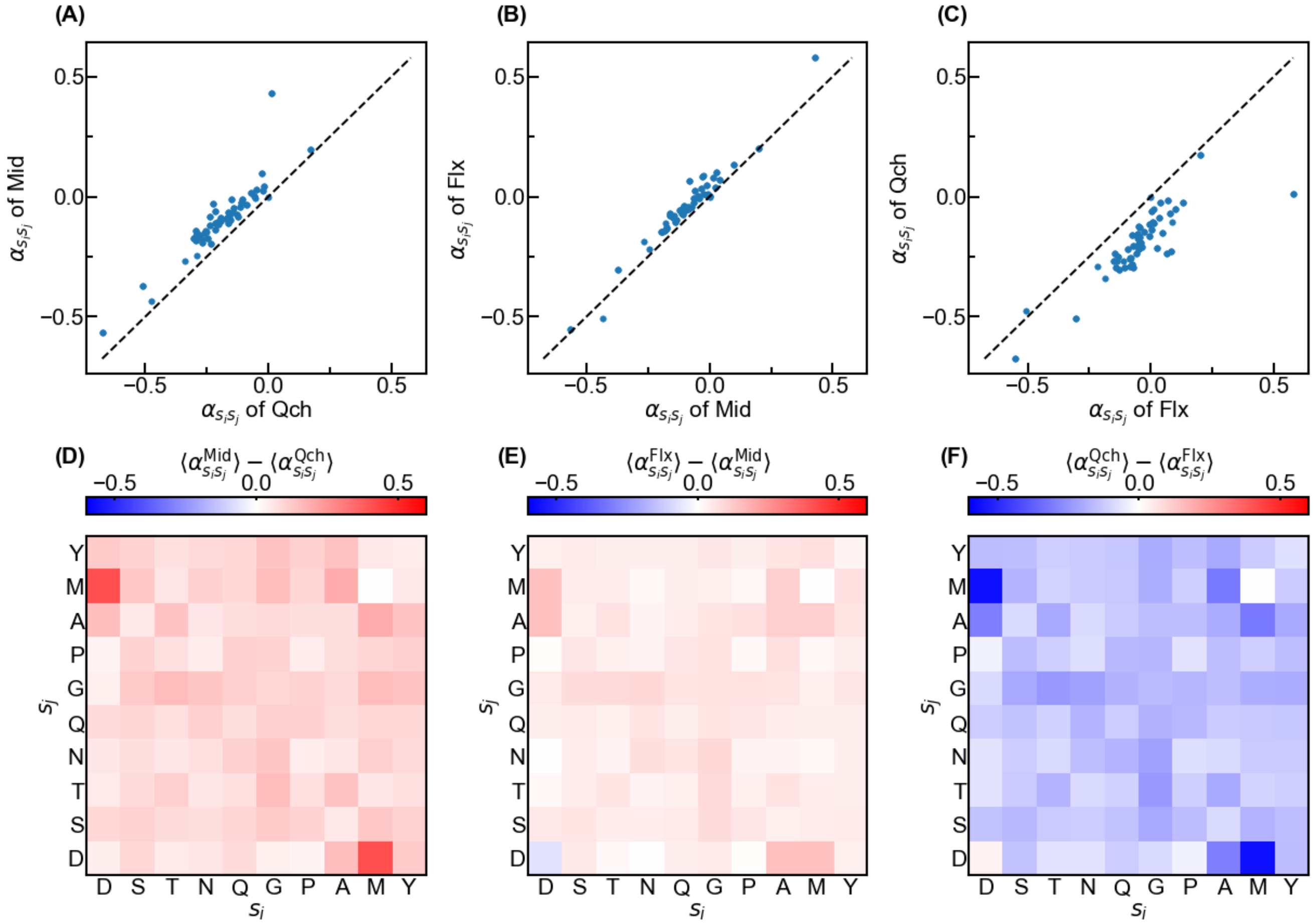

Figure S1: Cross-model comparison of residue-type interaction coefficients. Pairwise scatter plots compare the optimized type-pair coefficients $\alpha_{s_is_j}$ (A) between Qch and Mid, (B) between Mid and Flx, and (C) between Flx and Qch. Dashed lines indicate equality between two compared models. (D–F) Differences between the corresponding model pairs are shown in matrix form.

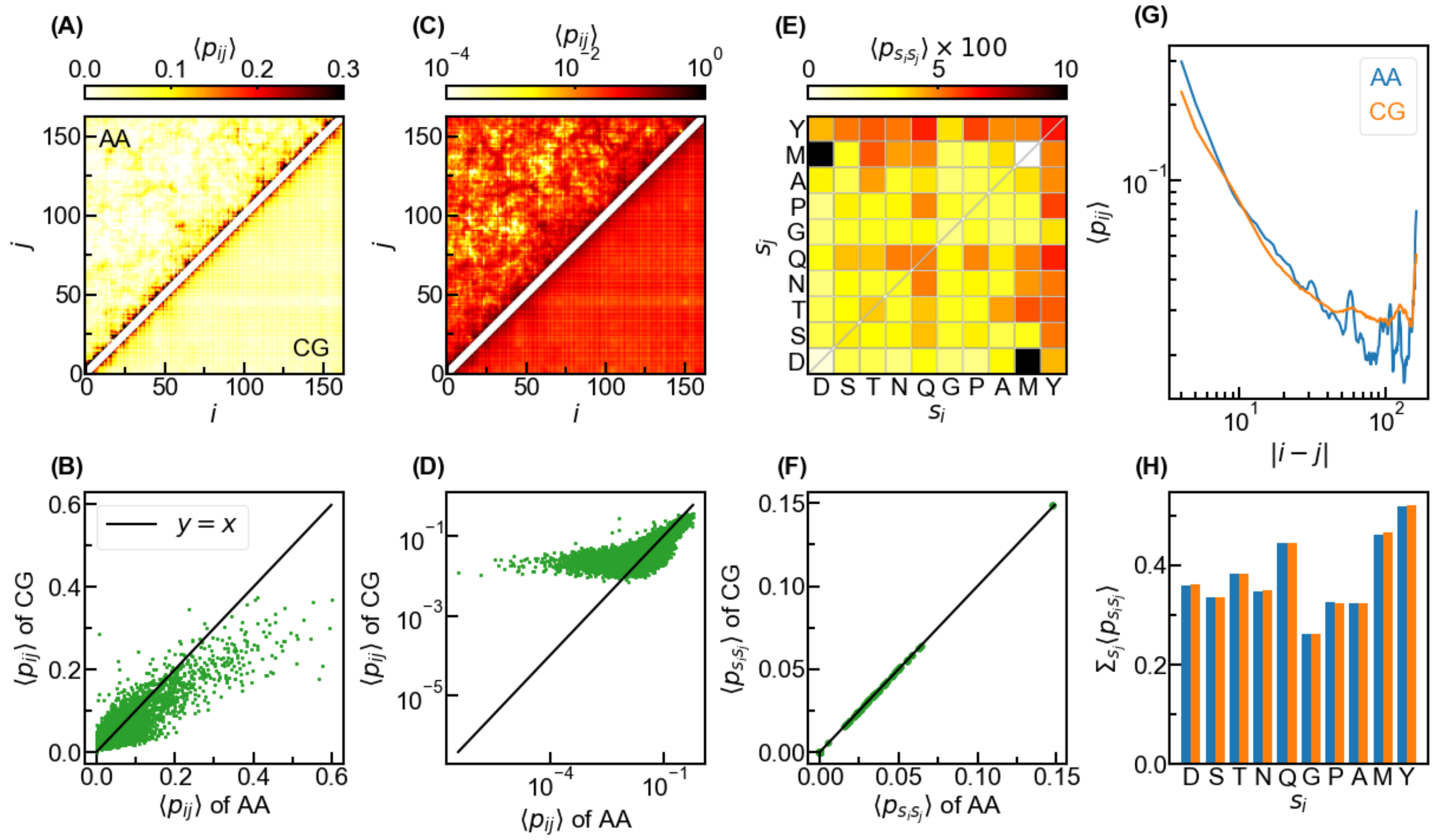

Figure S2: Reproduction of all-atom contact probabilities by the Qch coarse-grained model. (A) Comparison of residue-pair contact probability maps $\langle p_{ij} \rangle$ between the coarse-grained (CG) Qch model and the all-atom (AA) reference on linear color scales. The upper-left triangle shows the AA reference, and the lower-right triangle shows the CG model. (B) Scatter comparison of (A). (C) Same as (A), but on logarithmic color scales. (D) Same as (B), but on logarithmic scales. (E) Comparison of residue-type contact probability maps $\langle p_{s_i s_j} \rangle$. The upper-left triangle shows the AA reference, and the lower-right triangle shows the CG model. Diagonal cells are also split into upper-left and lower-right triangles. (F) Scatter comparison of (E). (G) Comparison of sequence-separation-averaged contact probability. (H) Comparison of residue-type summed contact propensities $\sum_{s_j} \langle p_{s_i s_j} \rangle$.

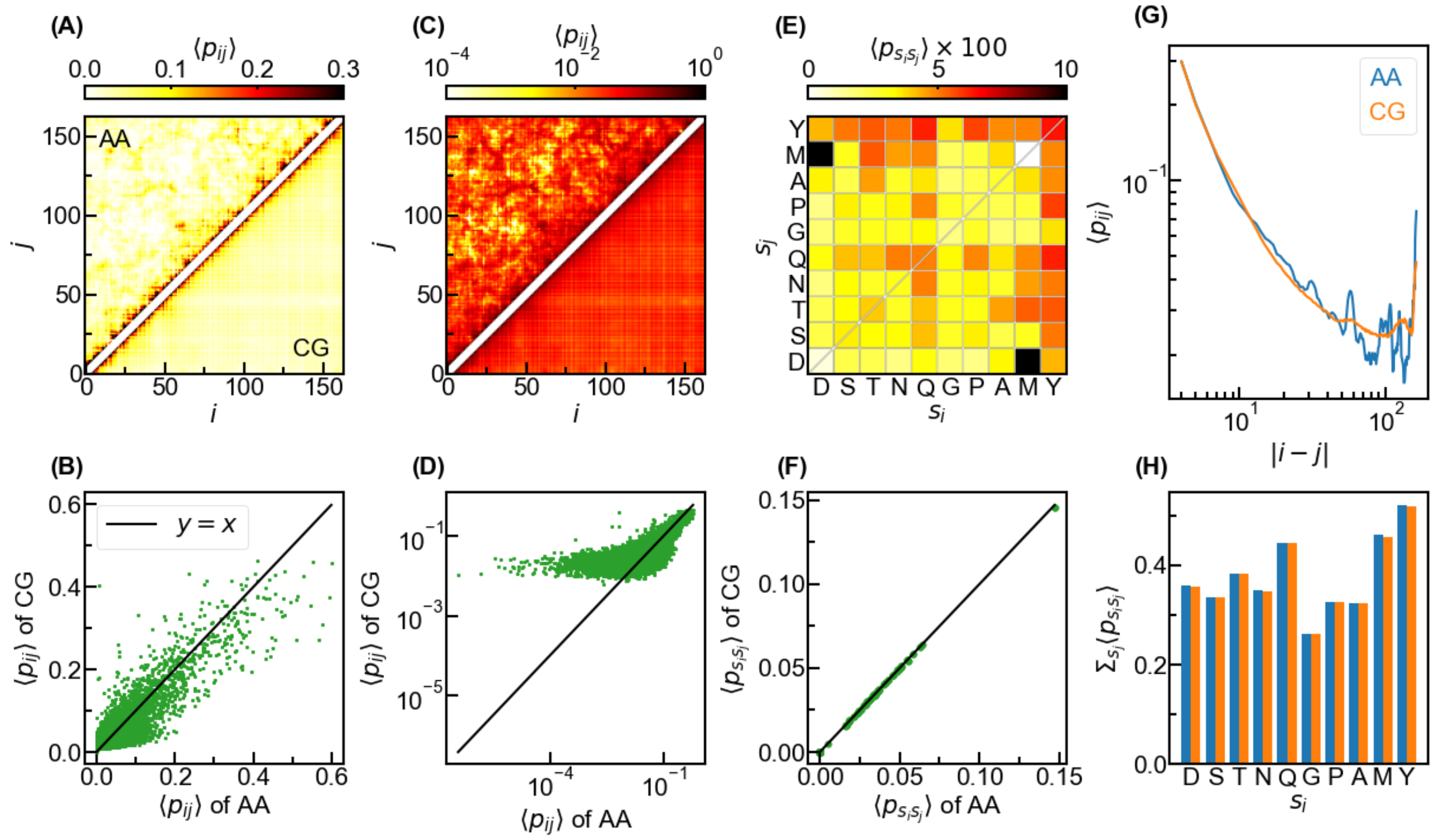

Figure S3: Reproduction of all-atom contact probabilities by the Mid coarse-grained model. (A) Comparison of residue-pair contact probability maps $\langle p_{ij} \rangle$ between the coarse-grained (CG) Mid model and the all-atom (AA) reference on linear color scales. The upper-left triangle shows the AA reference, and the lower-right triangle shows the CG model. (B) Scatter comparison of (A). (C) Same as (A), but on logarithmic color scales. (D) Same as (B), but on logarithmic scales. (E) Comparison of residue-type contact probability maps $\langle p_{s_i s_j} \rangle$. The upper-left triangle shows the AA reference, and the lower-right triangle shows the CG model. Diagonal cells are also split into upper-left and lower-right triangles. (F) Scatter comparison of (E). (G) Comparison of sequence-separation-averaged contact probability. (H) Comparison of residue-type summed contact propensities $\sum_{s_j} \langle p_{s_i s_j} \rangle$.

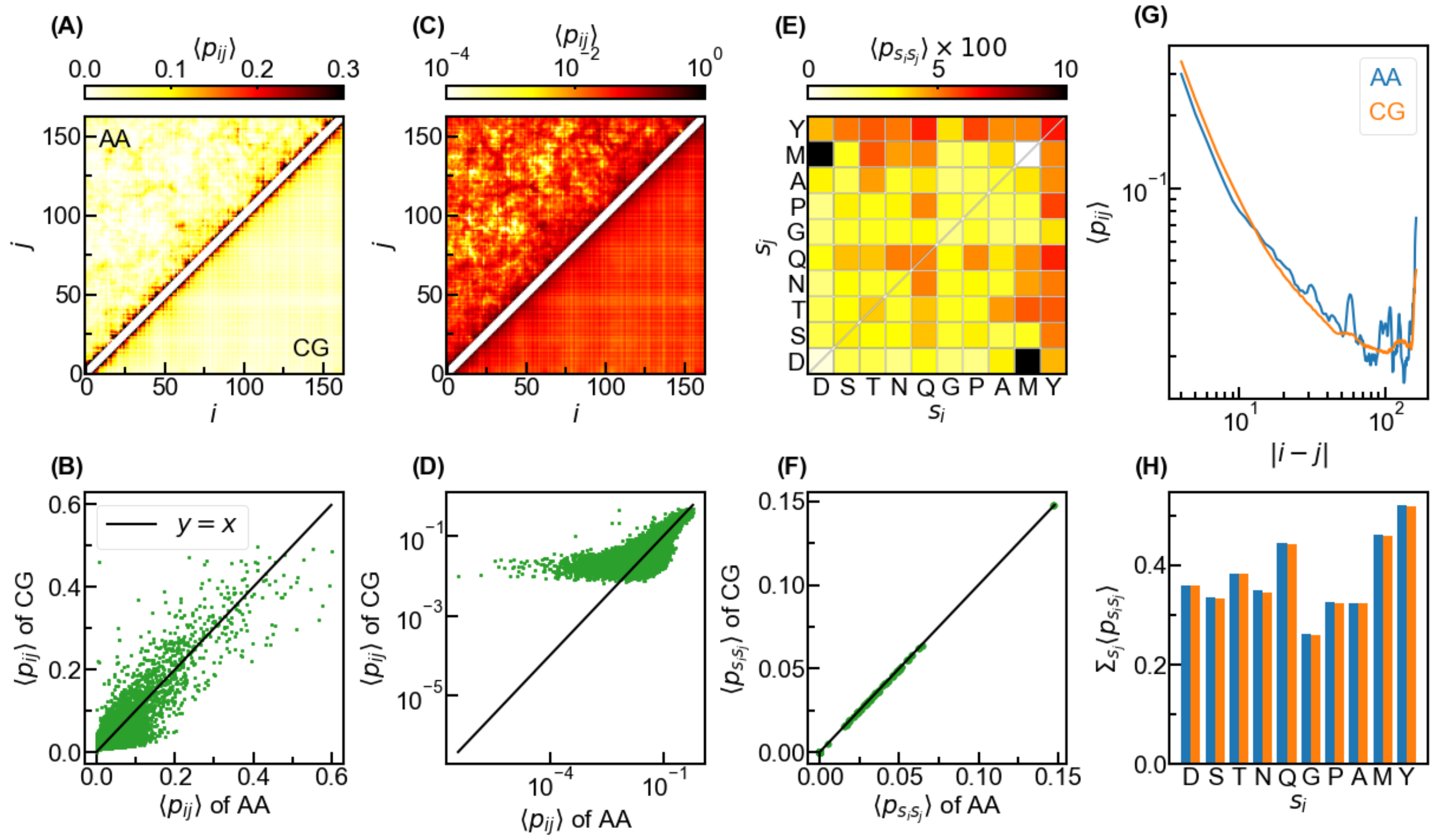

Figure S4: Reproduction of all-atom contact probabilities by the Flx coarse-grained model. (A) Comparison of residue-pair contact probability maps $\langle p_{ij} \rangle$ between the coarse-grained (CG) Flx model and the all-atom (AA) reference on linear color scales. The upper-left triangle shows the AA reference, and the lower-right triangle shows the CG model. (B) Scatter comparison of (A). (C) Same as (A), but on logarithmic color scales. (D) Same as (B), but on logarithmic scales. (E) Comparison of residue-type contact probability maps $\langle p_{s_i s_j} \rangle$. The upper-left triangle shows the AA reference, and the lower-right triangle shows the CG model. Diagonal cells are also split into upper-left and lower-right triangles. (F) Scatter comparison of (E). (G) Comparison of sequence-separation-averaged contact probability. (H) Comparison of residue-type summed contact propensities $\sum_{s_j} \langle p_{s_i s_j} \rangle$.

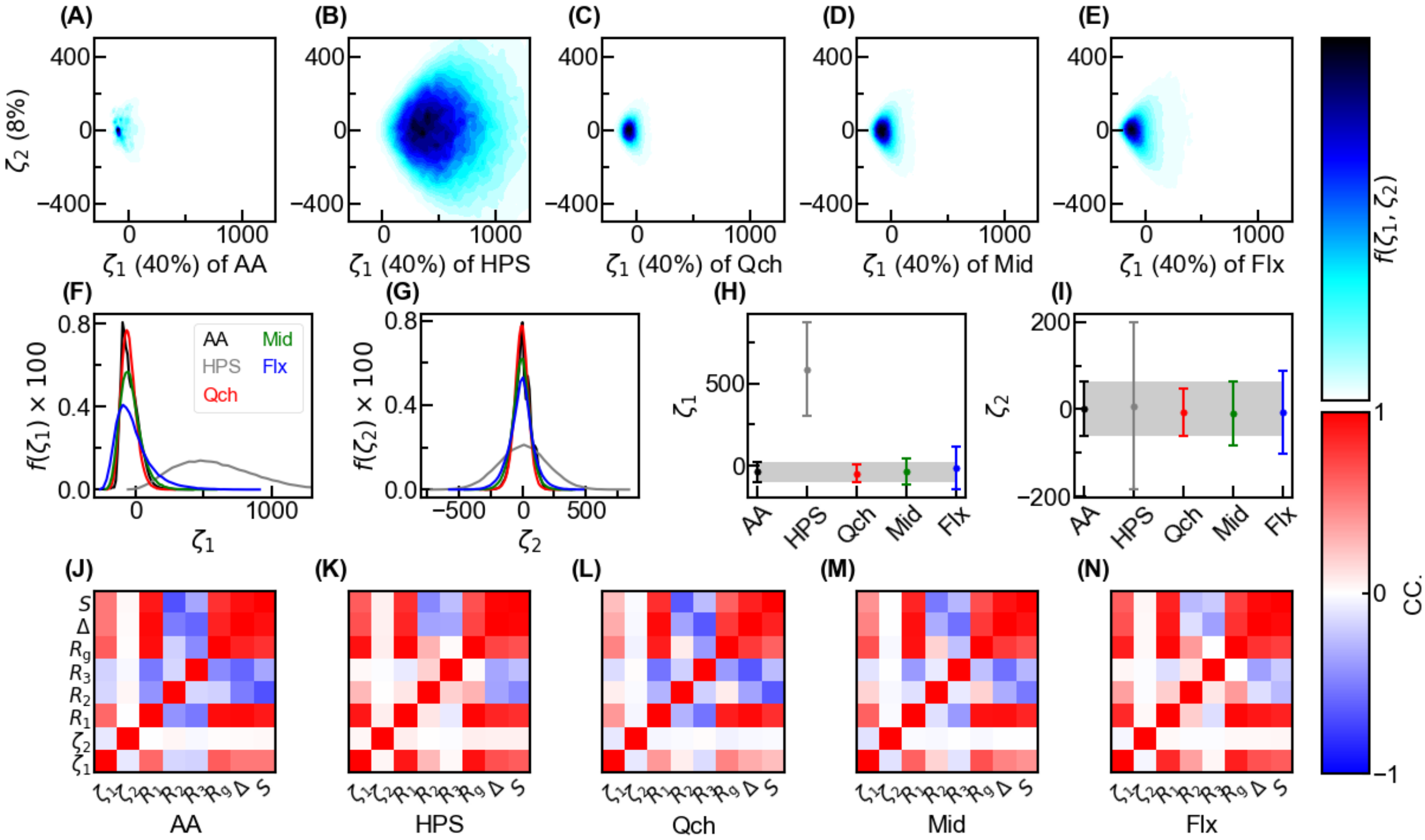

Figure S5: Principal component and shape-space comparison of isolated-chain conformational ensembles. (A–E) Joint probability density $f(\zeta_1, \zeta_2)$ for the first two principal component analysis (PCA) coordinates ζ_1 and ζ_2 of the ensemble from all-atom (AA), hydropathy scale (HPS), Qch, Mid, and Flx models. (F,G) Marginal distributions of ζ_1 and ζ_2. (H,I) Mean and standard deviation of ζ_1 and ζ_2 for each ensemble, with the gray band marking the AA mean ± standard deviation. (J–N) Correlation coefficient matrices among ζ_1, ζ_2, principal radii R_1–R_3, radius of gyration (R_g), asphericity (Δ), and shape parameter (S).

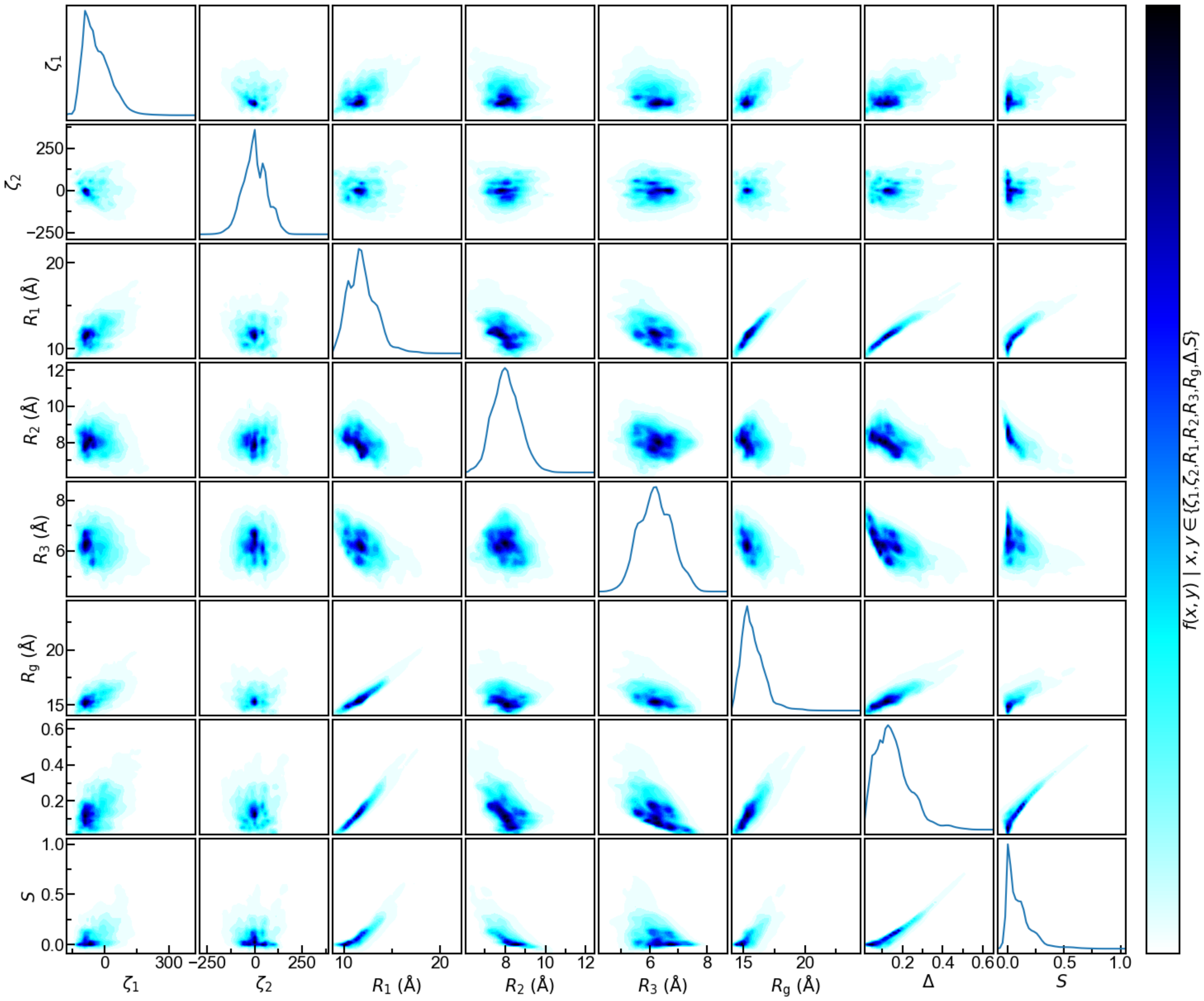

Figure S6: Full pair-distribution matrix for the all-atom isolated-chain ensemble. Diagonal panels show one-dimensional probability densities, and off-diagonal panels show two-dimensional joint probability densities among the principal component coordinates ζ_1 and ζ_2, principal radii R_1–R_3, radius of gyration (R_g), asphericity (Δ), and shape parameter (S).

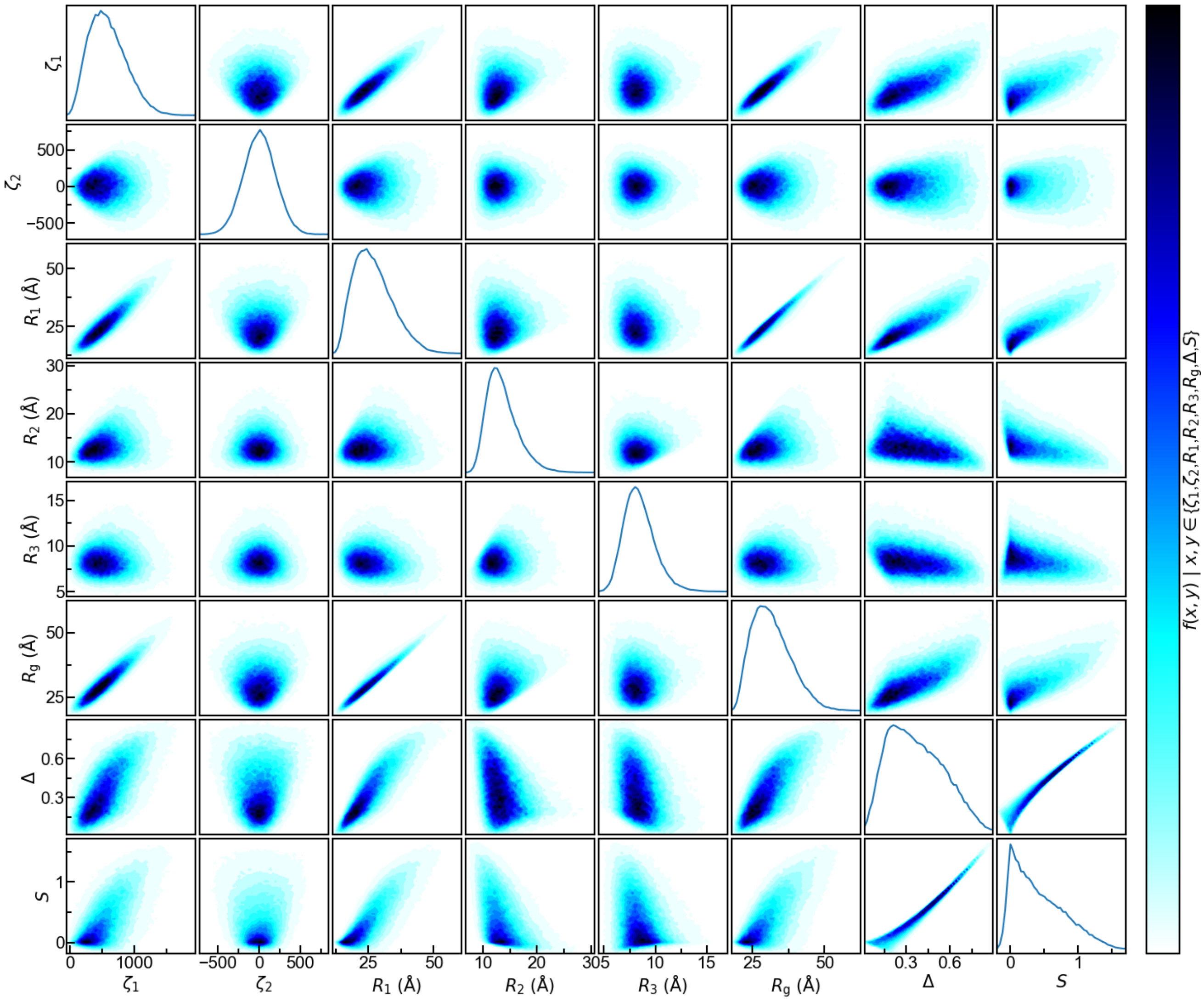

Figure S7: Full pair-distribution matrix for the hydropathy scale (HPS) isolated-chain ensemble. Diagonal panels show one-dimensional probability densities, and off-diagonal panels show two-dimensional joint probability densities among the principal component coordinates ζ_1 and ζ_2, principal radii R_1–R_3, radius of gyration (R_g), asphericity (Δ), and shape parameter (S).

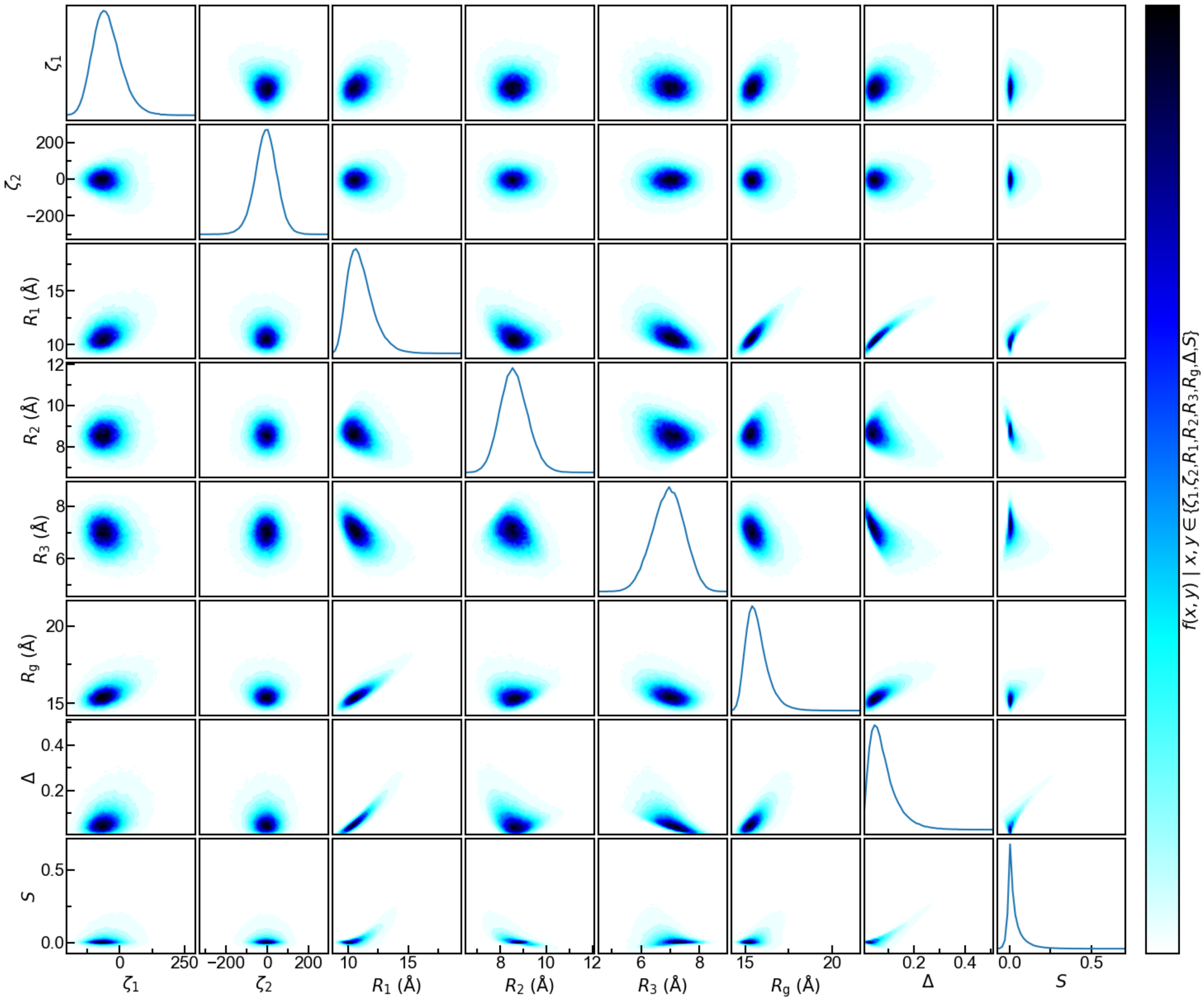

Figure S8: Full pair-distribution matrix for the Qch isolated-chain ensemble. Diagonal panels show one-dimensional probability densities, and off-diagonal panels show two-dimensional joint probability densities among the principal component coordinates ζ_1 and ζ_2, principal radii R_1–R_3, radius of gyration (R_g), asphericity (Δ), and shape parameter (S).

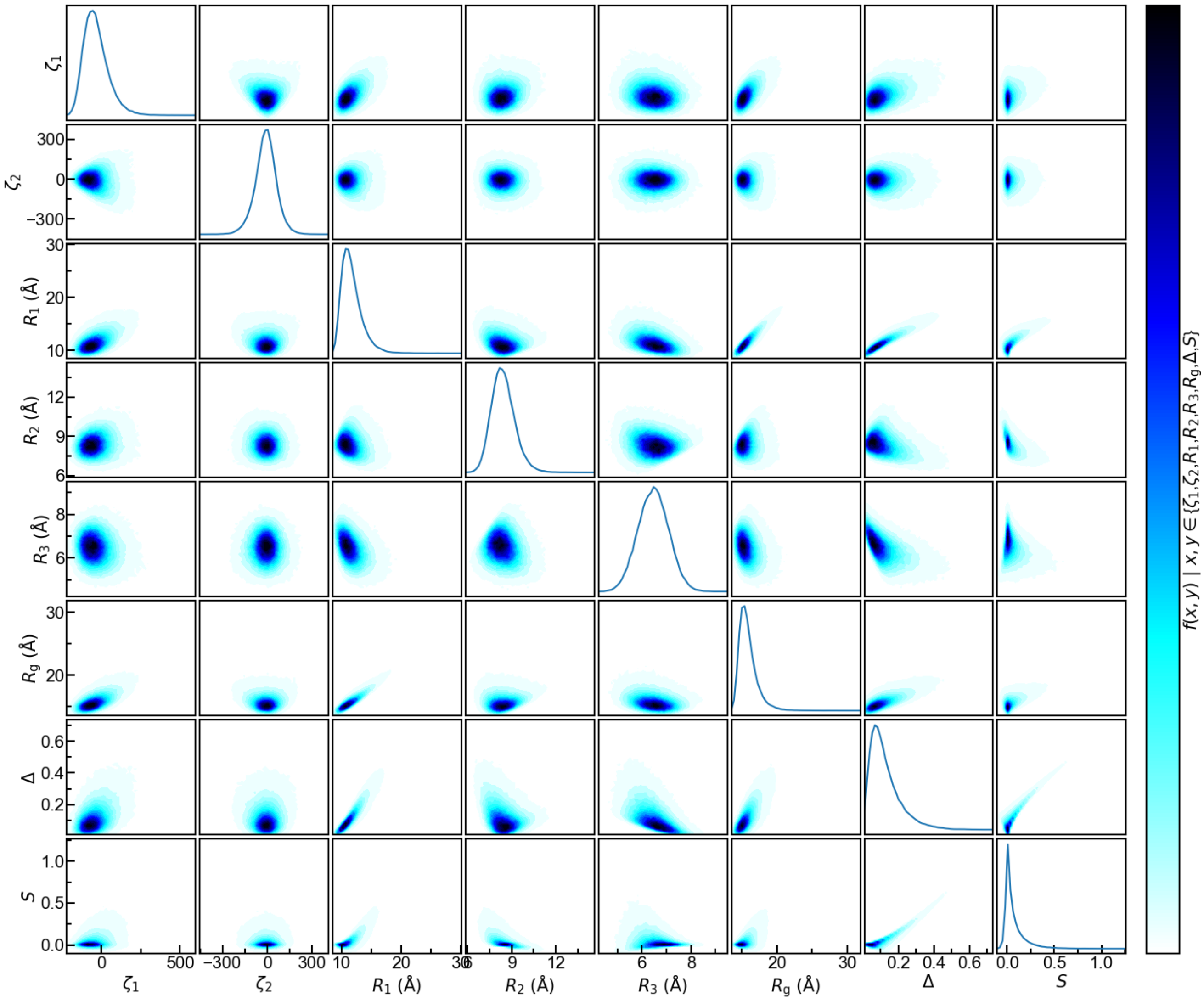

Figure S9: Full pair-distribution matrix for the Mid isolated-chain ensemble. Diagonal panels show one-dimensional probability densities, and off-diagonal panels show two-dimensional joint probability densities among the principal component coordinates ζ_1 and ζ_2, principal radii R_1–R_3, radius of gyration (R_g), asphericity (Δ), and shape parameter (S).

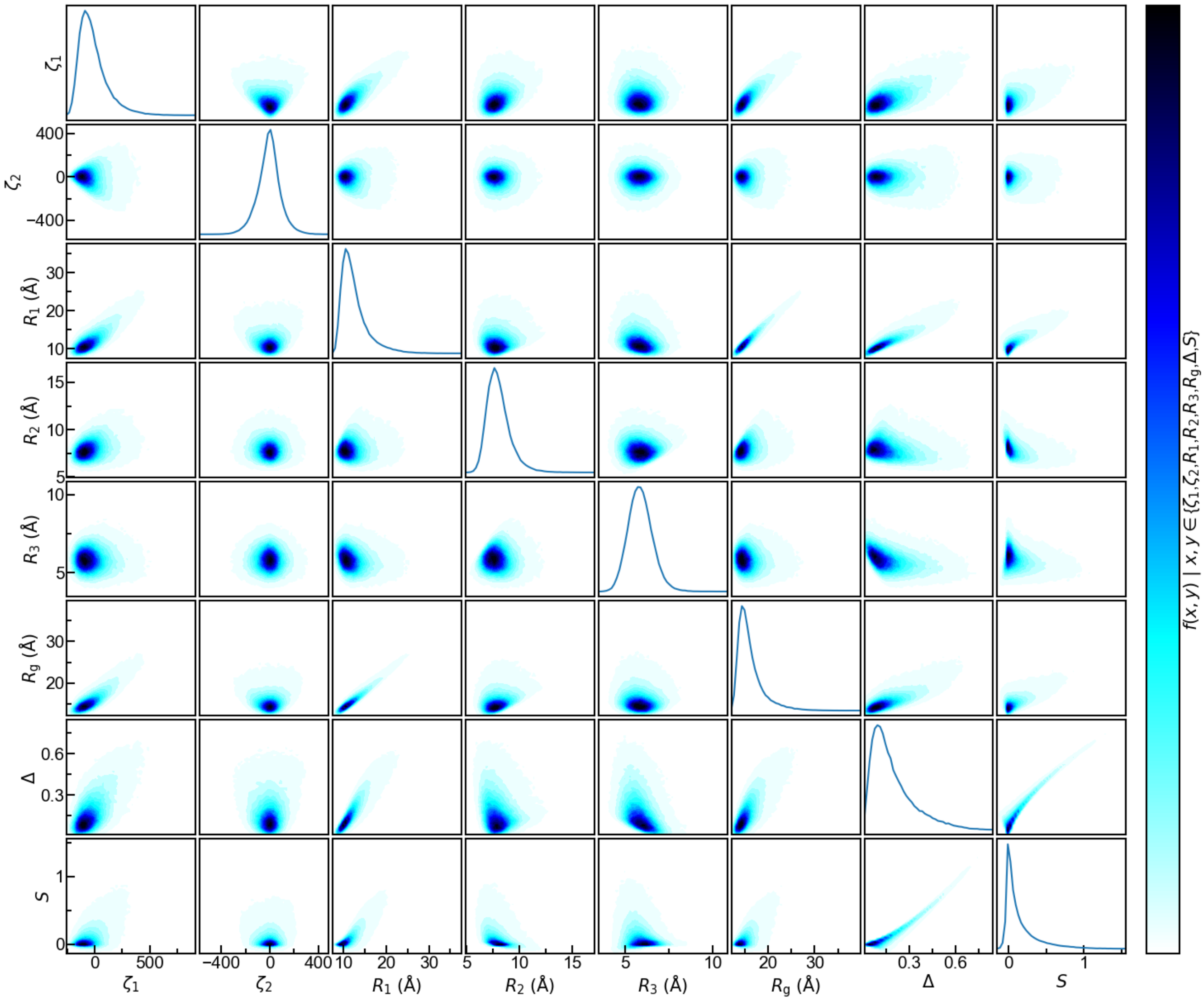

Figure S10: Full pair-distribution matrix for the Flx isolated-chain ensemble. Diagonal panels show one-dimensional probability densities, and off-diagonal panels show two-dimensional joint probability densities among the principal component coordinates ζ_1 and ζ_2, principal radii R_1–R_3, radius of gyration (R_g), asphericity (Δ), and shape parameter (S).

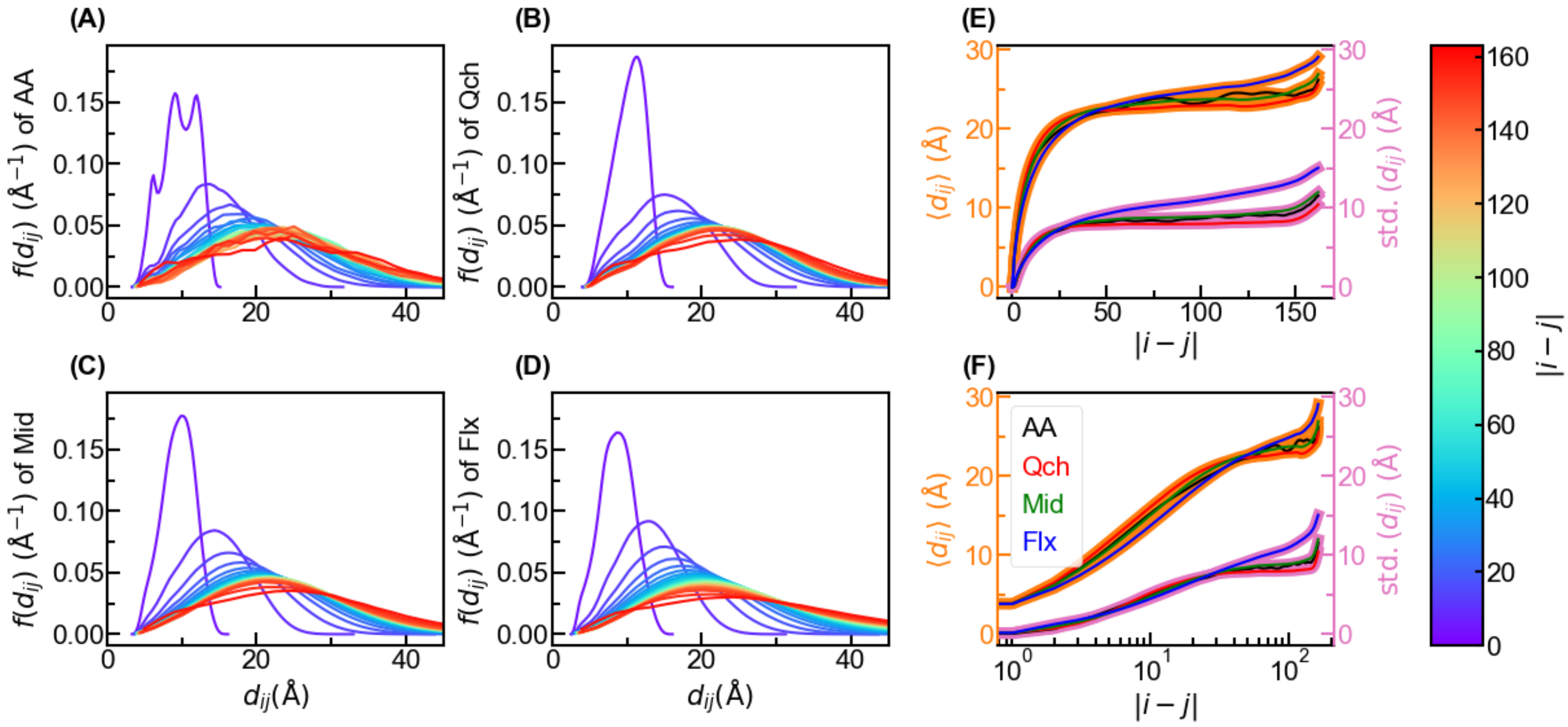

Figure S11: Intrachain residue-pair distance distributions in the isolated state. (A–D) Probability density $f(d_{ij})$ of the three-dimensional distance d_{ij} for all-atom (AA), Qch, Mid, and Flx ensembles; curves are colored by sequence separation $|i - j|$. (E,F) Mean distance $\langle d_{ij} \rangle$ and standard deviation std.(d_{ij}) as functions of $|i - j|$ on linear and logarithmic sequence-separation axes.

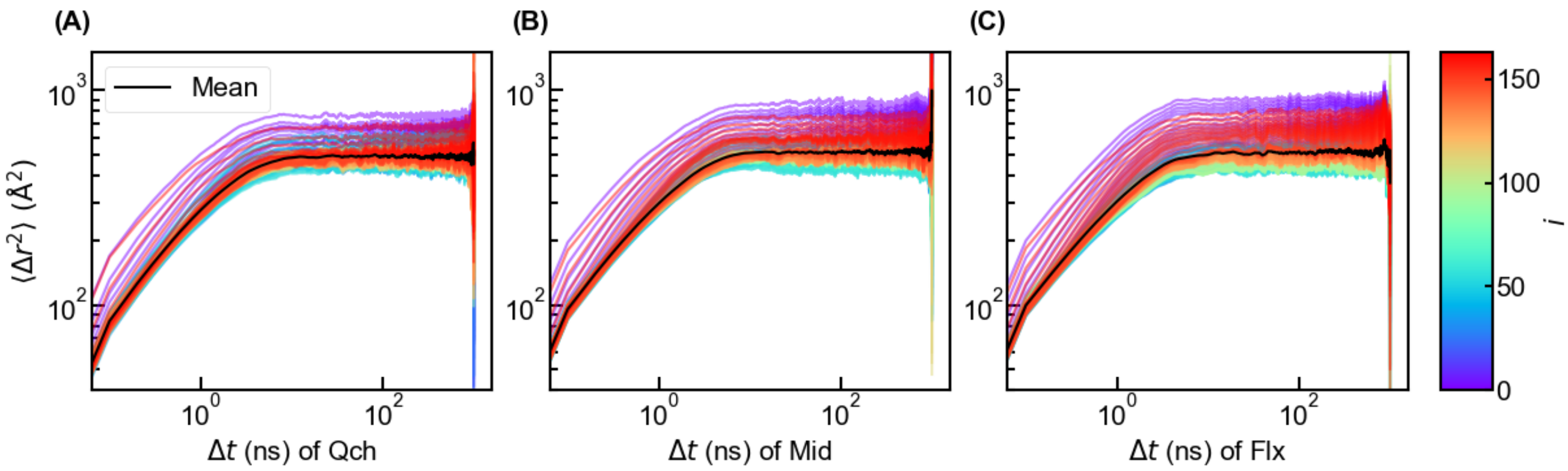

Figure S12: Residue-resolved isolated-chain diffusion dynamics. (A–C) Mean-squared displacement (MSD), $\langle\Delta r^2\rangle$, of each residue relative to the chain center of mass for Qch, Mid, and Flx, respectively. Curves are colored by residue index i, and the black curve shows the residue average.

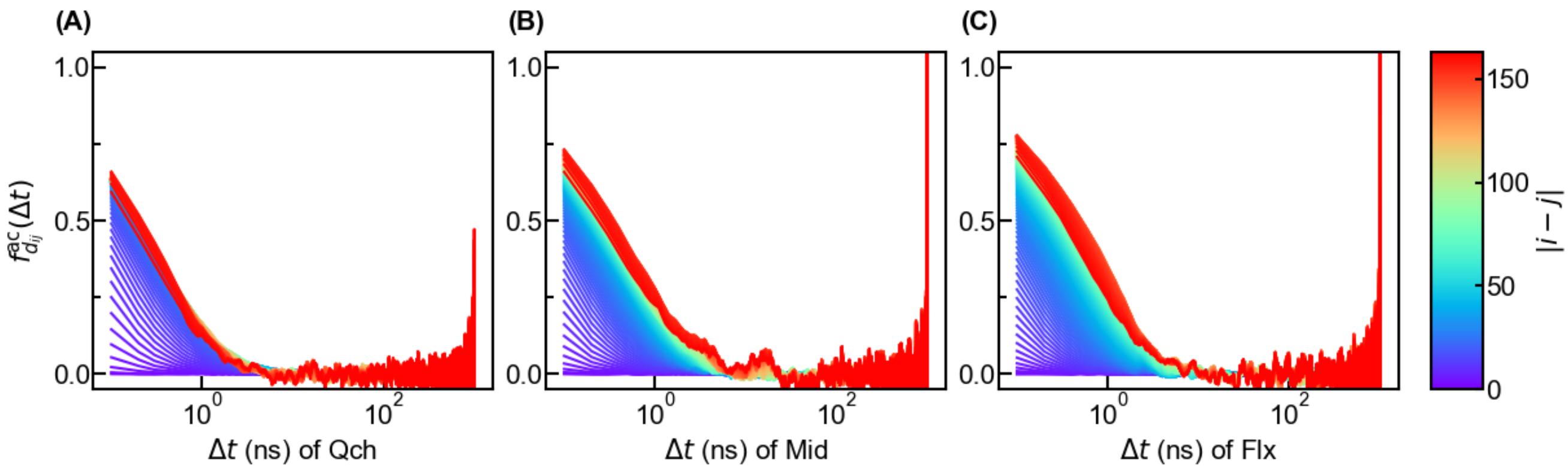

Figure S13: Autocorrelation of intrachain residue-pair distances in isolated-chain simulations. (A–C) Distance autocorrelation function $f^{\mathrm{ac}}_{d_{ij}}(\Delta t)$ for Qch, Mid, and Flx, respectively. Curves are averaged over residue pairs with the same sequence separation and colored by $|i - j|$.

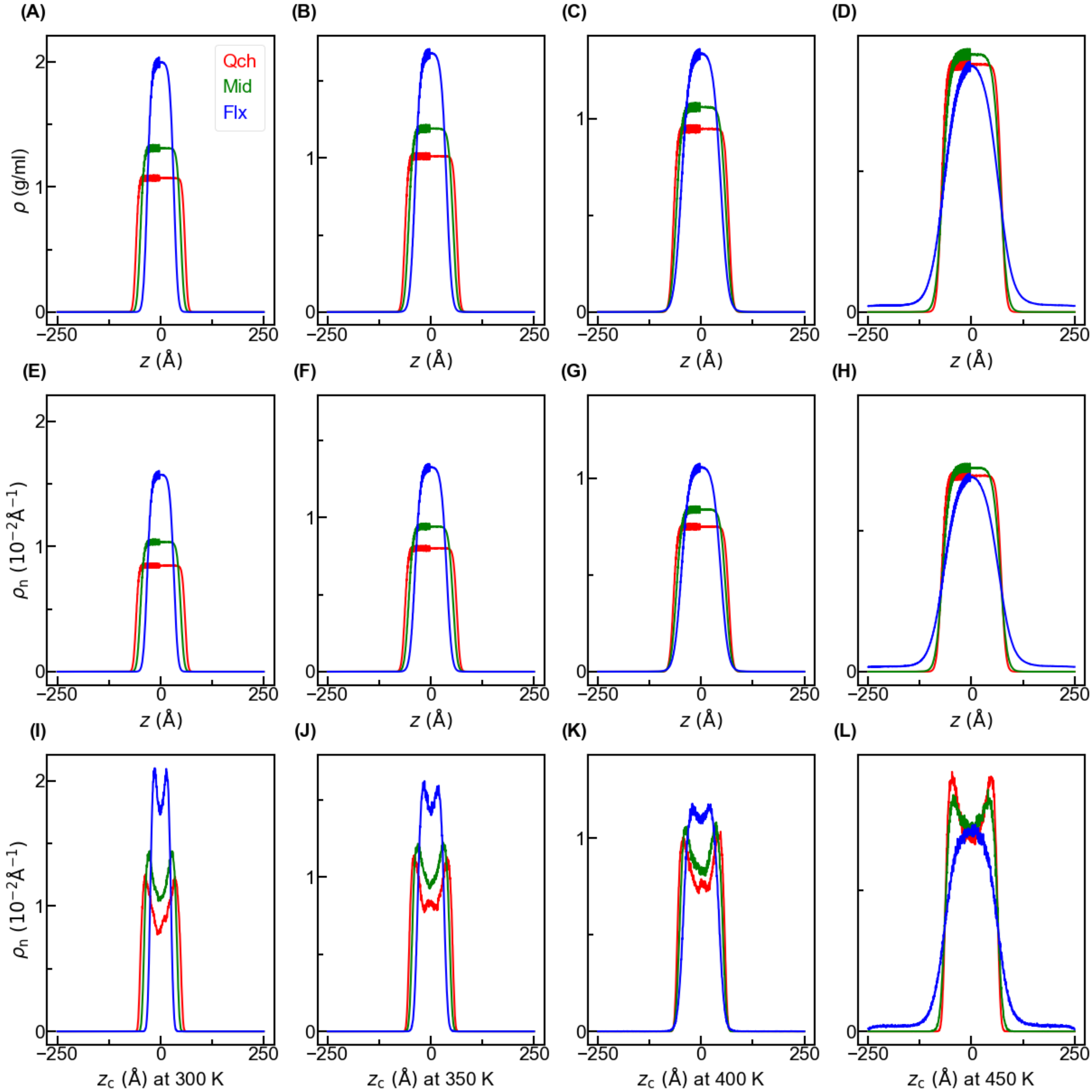

Figure S14: Slab density profiles compared across models at fixed temperature. Rows show the mass density $\rho(z)$, the residue number density $\rho_{\mathrm{nr}}(z)$, and the chain number density $\rho_{\mathrm{nc}}(z)$ along the slab coordinate. Columns correspond to 300, 350, 400, and 450 K. Curves compare Qch, Mid, and Flx.

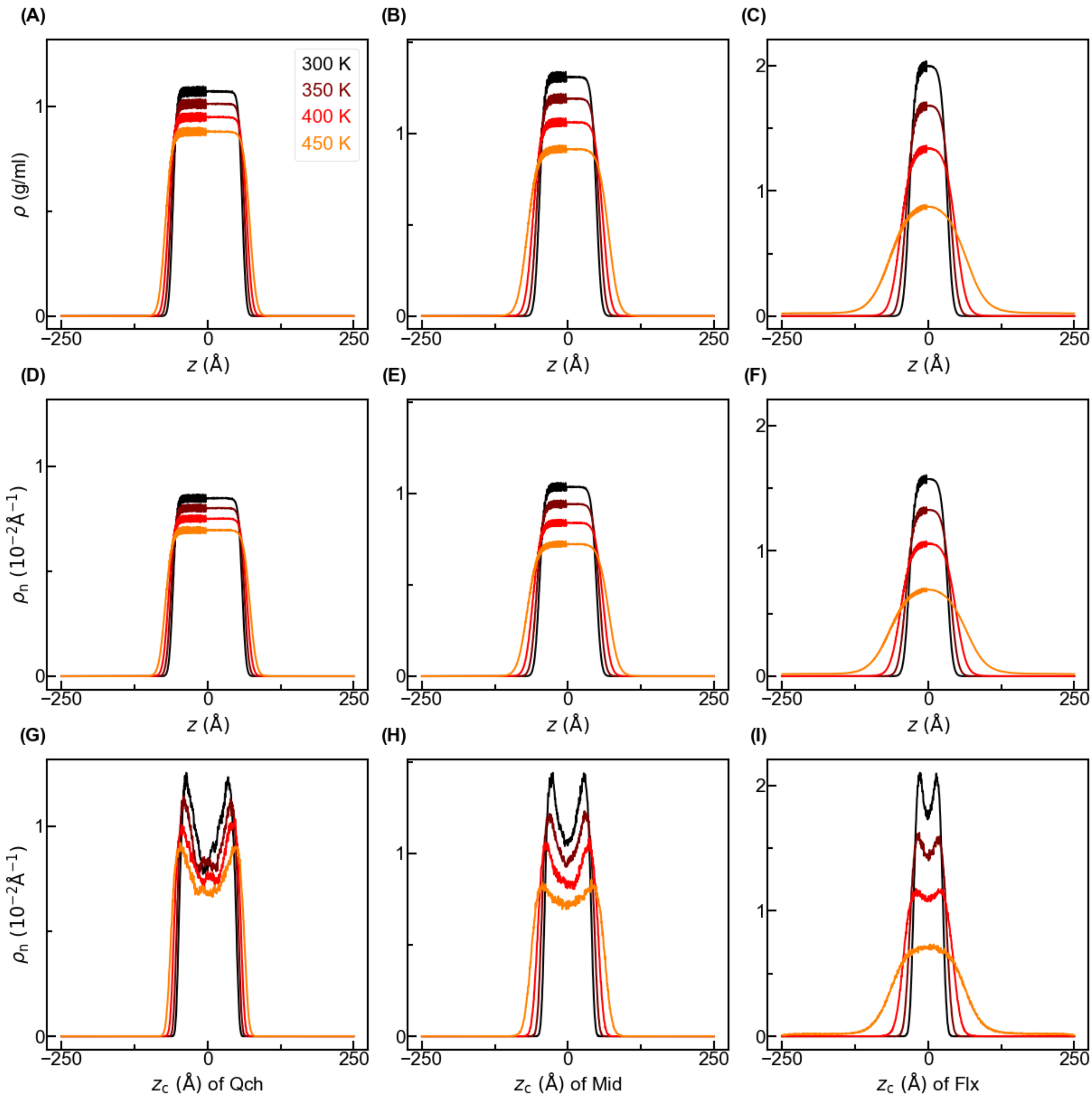

Figure S15: Slab density profiles compared across temperatures for each model. Rows show the mass density $\rho(z)$, the residue number density $\rho_{\rm nr}(z)$, and the chain number density $\rho_{\rm nc}(z)$ along the slab coordinate. Columns correspond to Qch, Mid, and Flx. Curves compare 300, 350, 400, and 450 K.

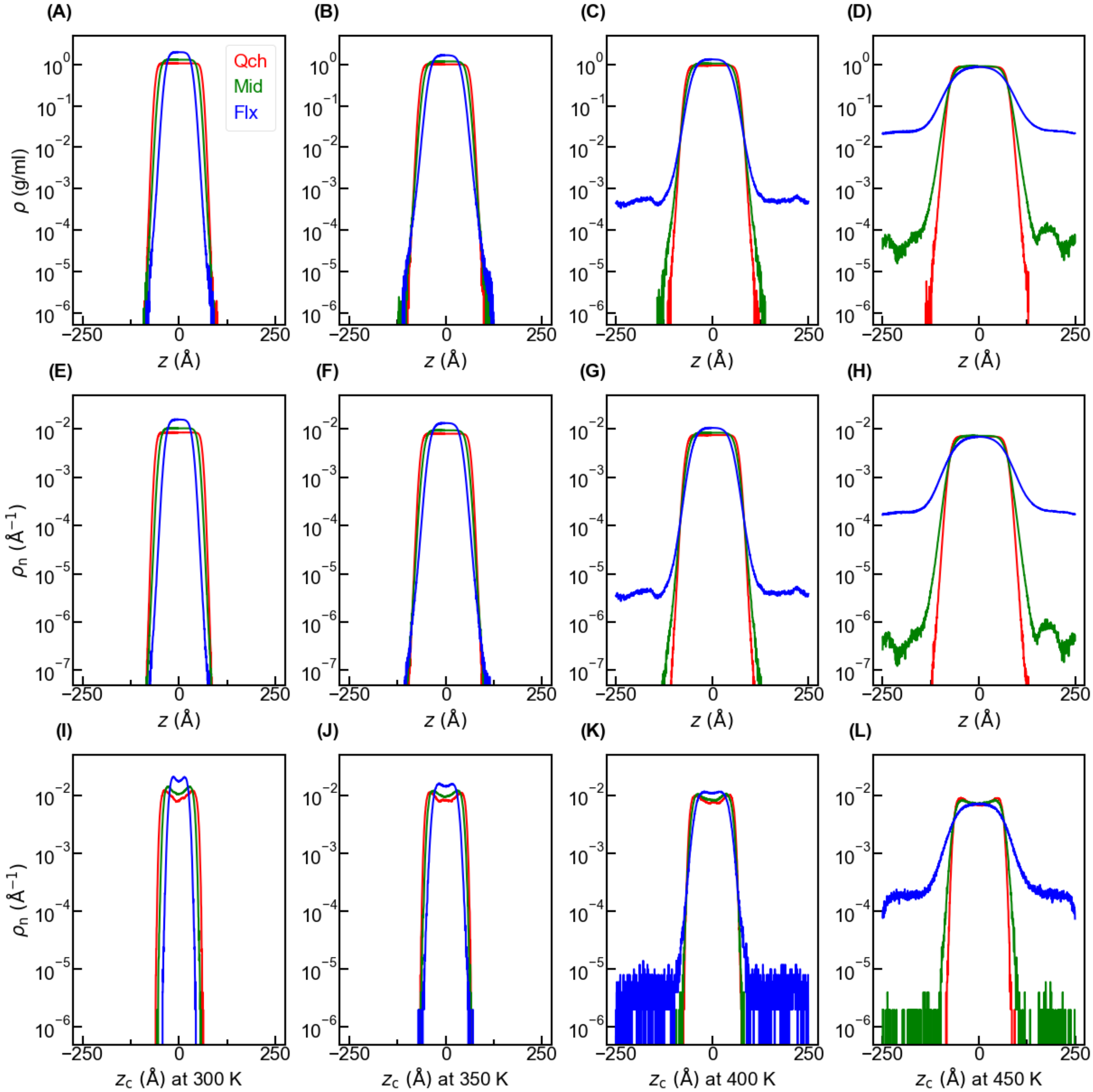

Figure S16: Log-scale slab density profiles compared across models at fixed temperature. Rows show $\rho(z)$, $\rho_{\rm nr}(z)$, and $\rho_{\rm nc}(z)$ on logarithmic density axes, emphasizing dilute-phase tails and interfacial regions. Columns correspond to 300, 350, 400, and 450 K. Curves compare Qch, Mid, and Flx.

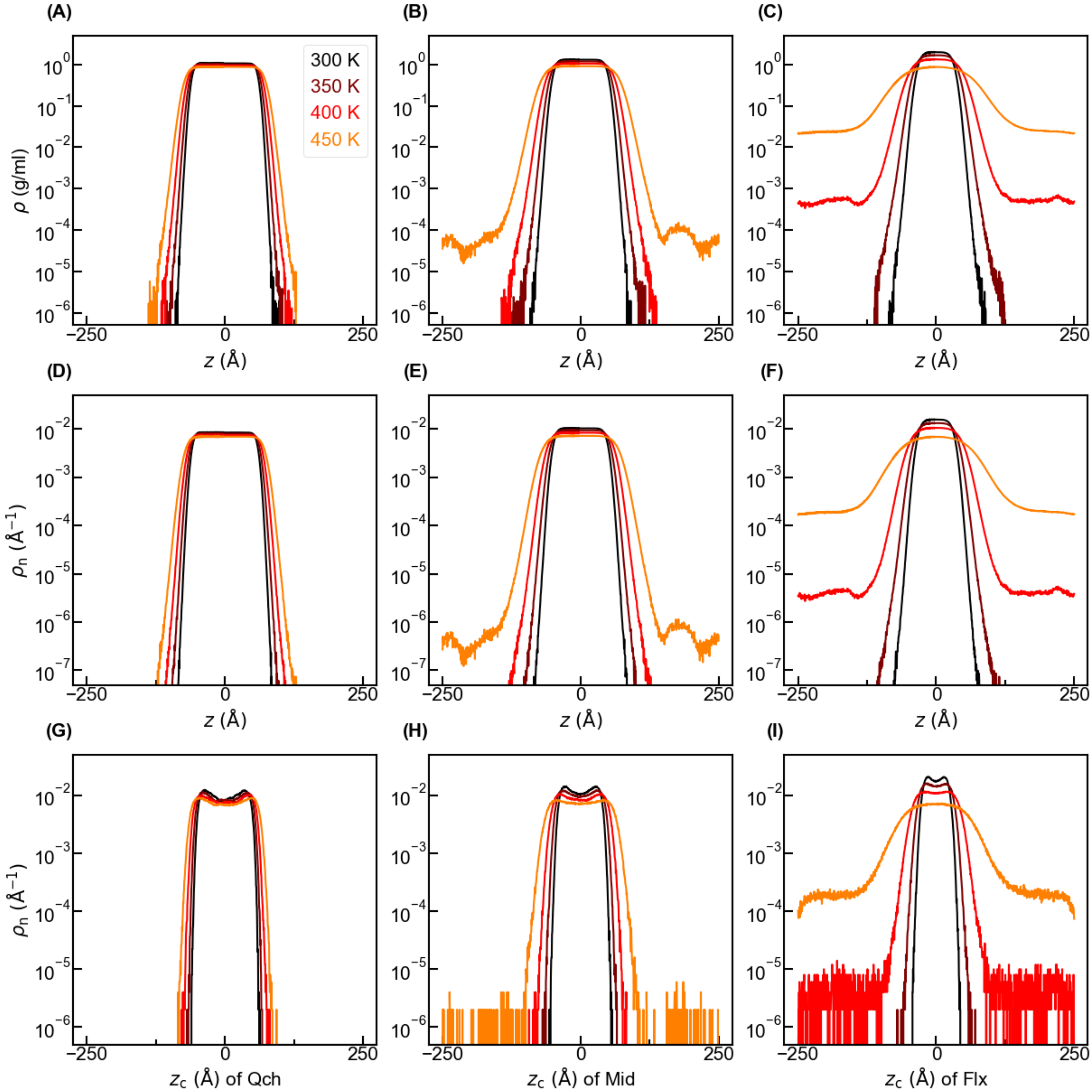

Figure S17: Log-scale slab density profiles compared across temperatures for each model. Rows show $\rho(z)$, $\rho_{\mathrm{nr}}(z)$, and $\rho_{\mathrm{nc}}(z)$ on logarithmic density axes, emphasizing dilute-phase tails and interfacial regions. Columns correspond to Qch, Mid, and Flx. Curves compare 300, 350, 400, and 450 K.

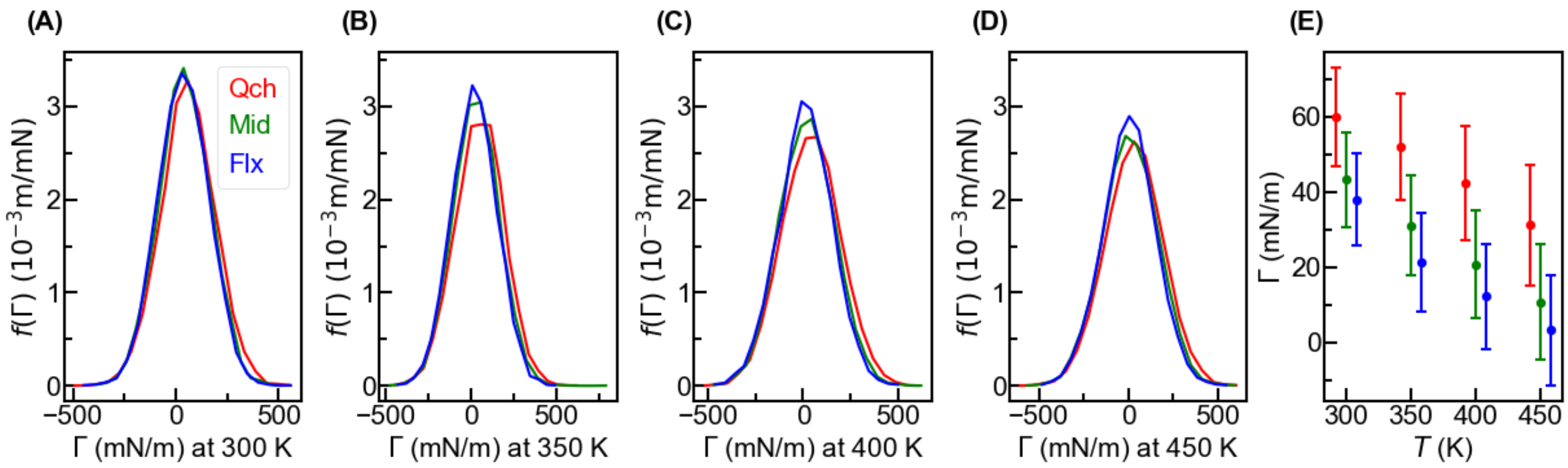

Figure S18: Surface tension distributions compared across models at fixed temperature. (A–D) Probability density $f(\Gamma)$ of the surface tension Γ at 300, 350, 400, and 450 K for Qch, Mid, and Flx. (E) Mean Γ with standard deviation as a function of temperature. Because the single-frame surface tension fluctuates strongly, the mean surface tension over a 100-frame window was treated as a statistical unit for presenting the standard deviation.

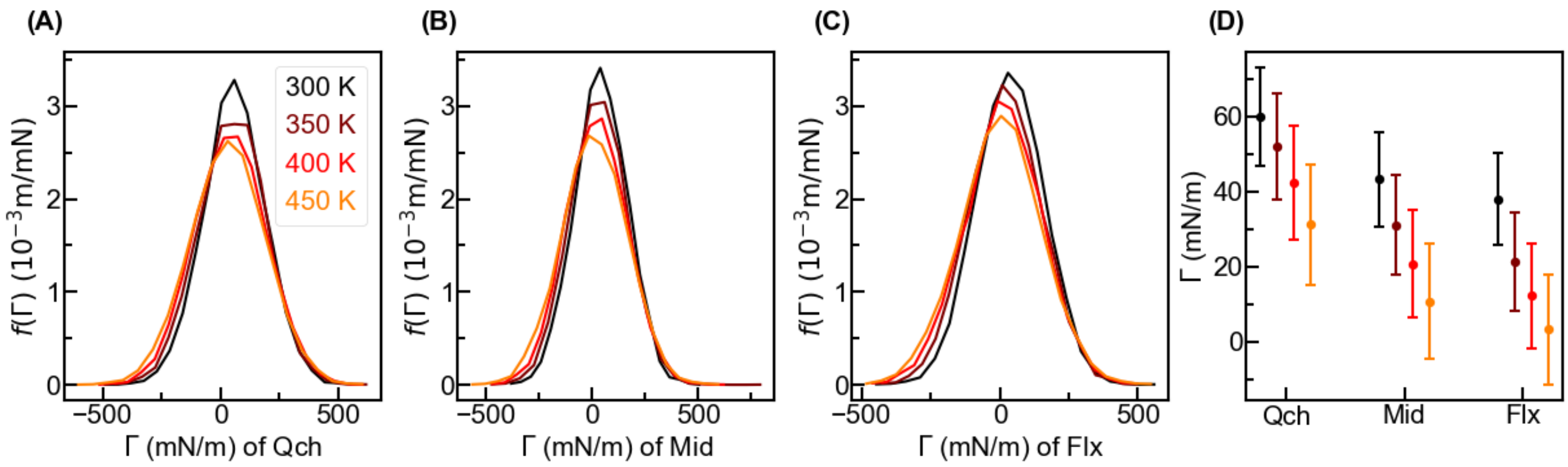

Figure S19: Surface tension distributions compared across temperatures for each model. (A–C) Probability density $f(\Gamma)$ of surface tension Γ for Qch, Mid, and Flx, respectively, with curves colored by temperature. (D) Mean Γ with standard deviation grouped by model. Because the single-frame surface tension fluctuates strongly, the mean surface tension over a 100-frame window was treated as a statistical unit for presenting the standard deviation.

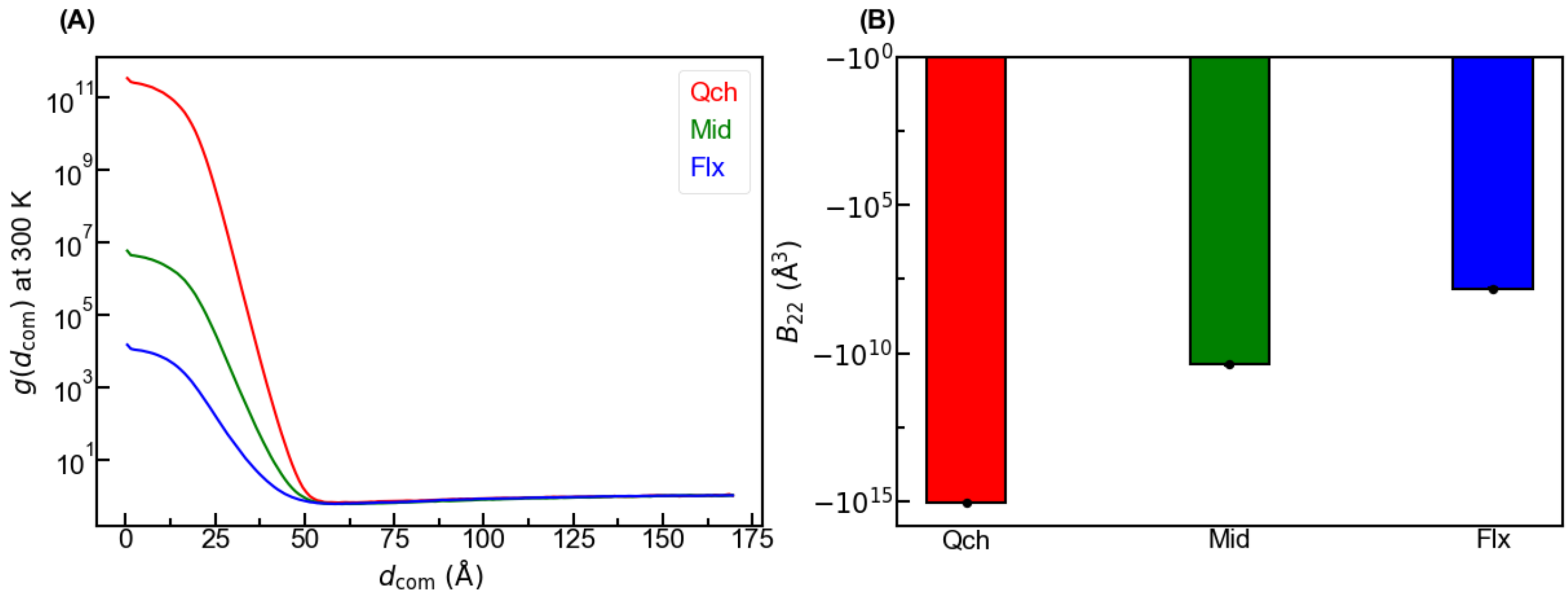

Figure S20: Interchain interaction strength at 300 K for each model. (A) The normalized radial distribution function $g(d_{\mathrm{com}})$ between the centers of mass of two chains. The light bands show standard deviations. (B) The second virial coefficient with standard deviation.

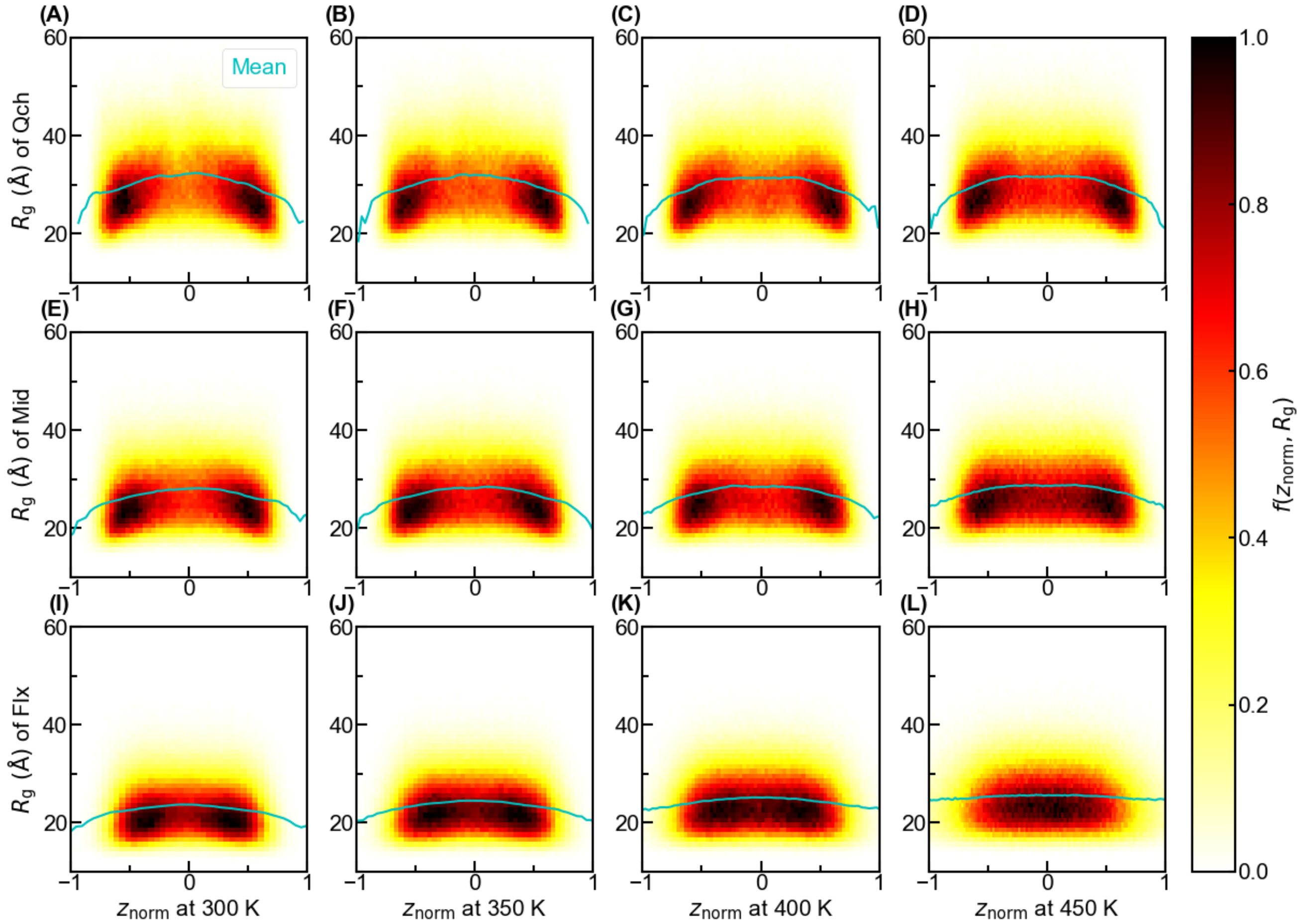

Figure S21: Position-resolved chain radius of gyration (R_g) in slab simulations. Rows correspond to Qch, Mid, and Flx; columns correspond to 300, 350, 400, and 450 K. Each panel shows the joint probability density $f(z_{norm}, R_g)$ of normalized slab position z_{norm} and R_g. Cyan curves denote the local mean R_g.

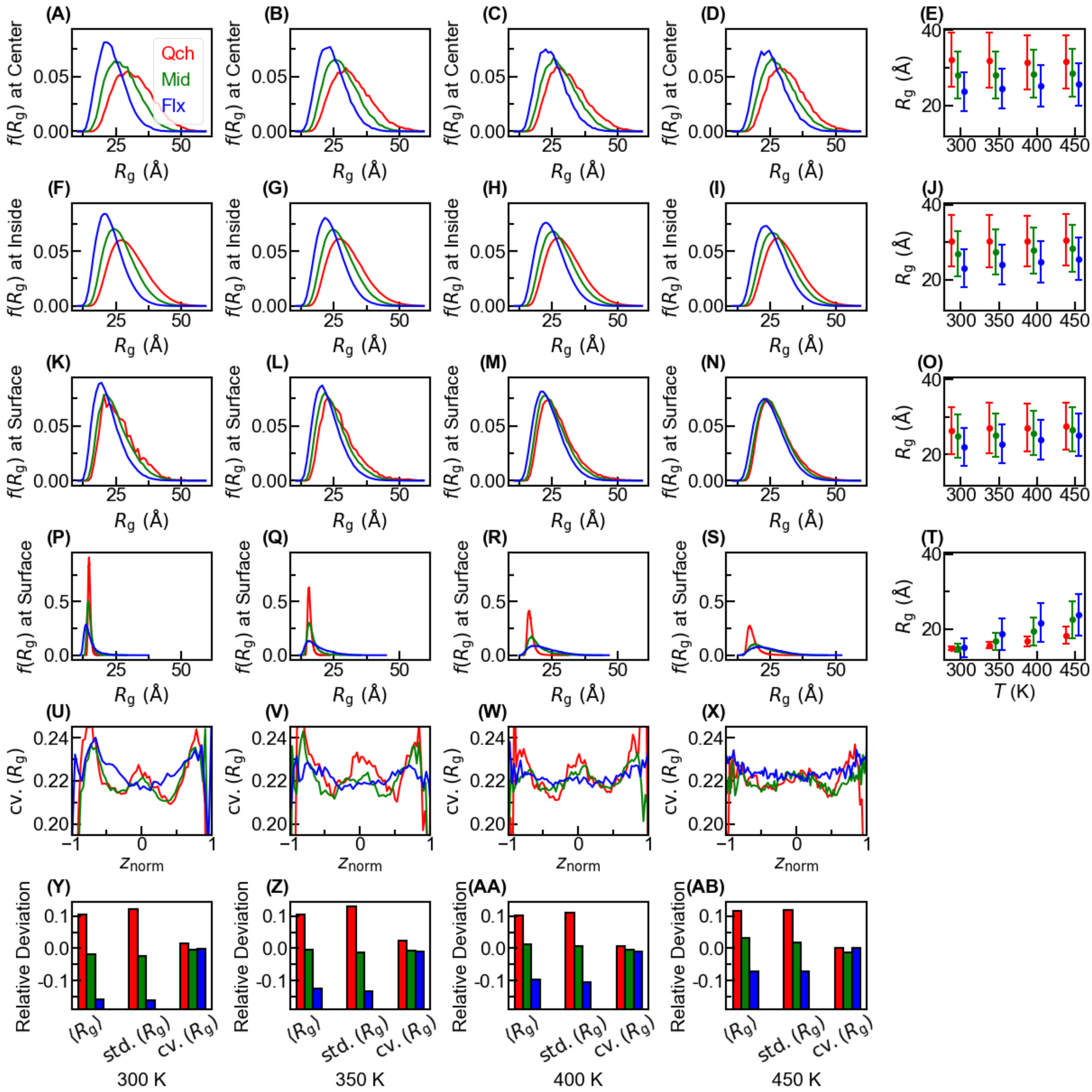

Figure S22: Model comparison of radius of gyration (R_g) distributions and moments at fixed temperature. Columns correspond to 300, 350, 400, and 450 K, with a summary column on the right. Rows show R_g distributions at the slab center, inside the condensed phase, at the surface, and in isolated-chain simulations; the local coefficient of variation cv.(R_g) along z_{norm}; and relative deviations of $\langle R_g \rangle$, std.(R_g), and cv.(R_g).

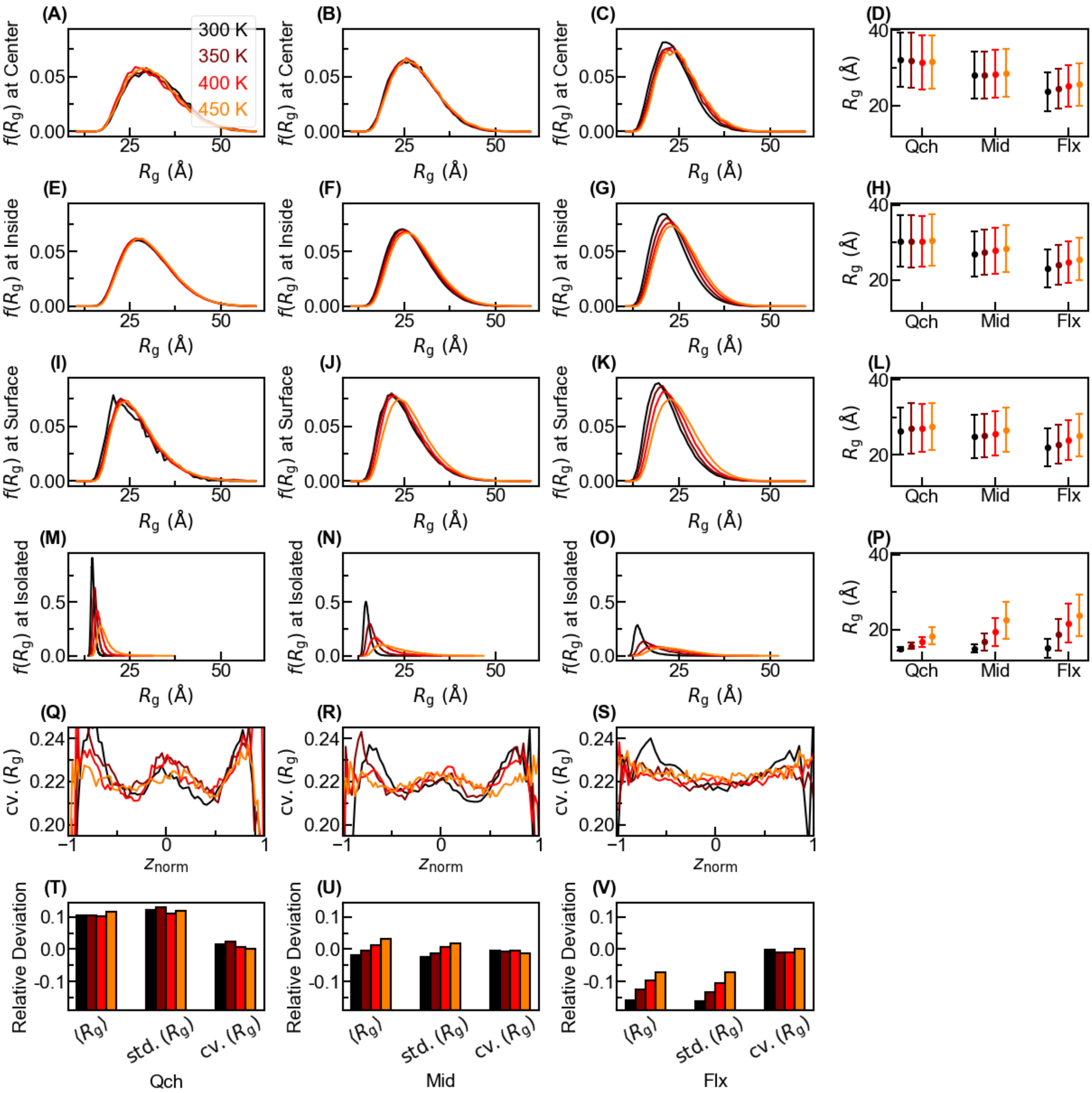

Figure S23: Temperature comparison of radius of gyration (R_g) distributions and moments within each model. Columns correspond to Qch, Mid, and Flx, with a summary column on the right. Rows show R_g distributions at the slab center, inside the condensed phase, at the surface, and in isolated-chain simulations; the local coefficient of variation cv.(R_g) along z_{norm}; and relative deviations of $\langle R_g \rangle$, std.(R_g), and cv.(R_g) across temperatures.

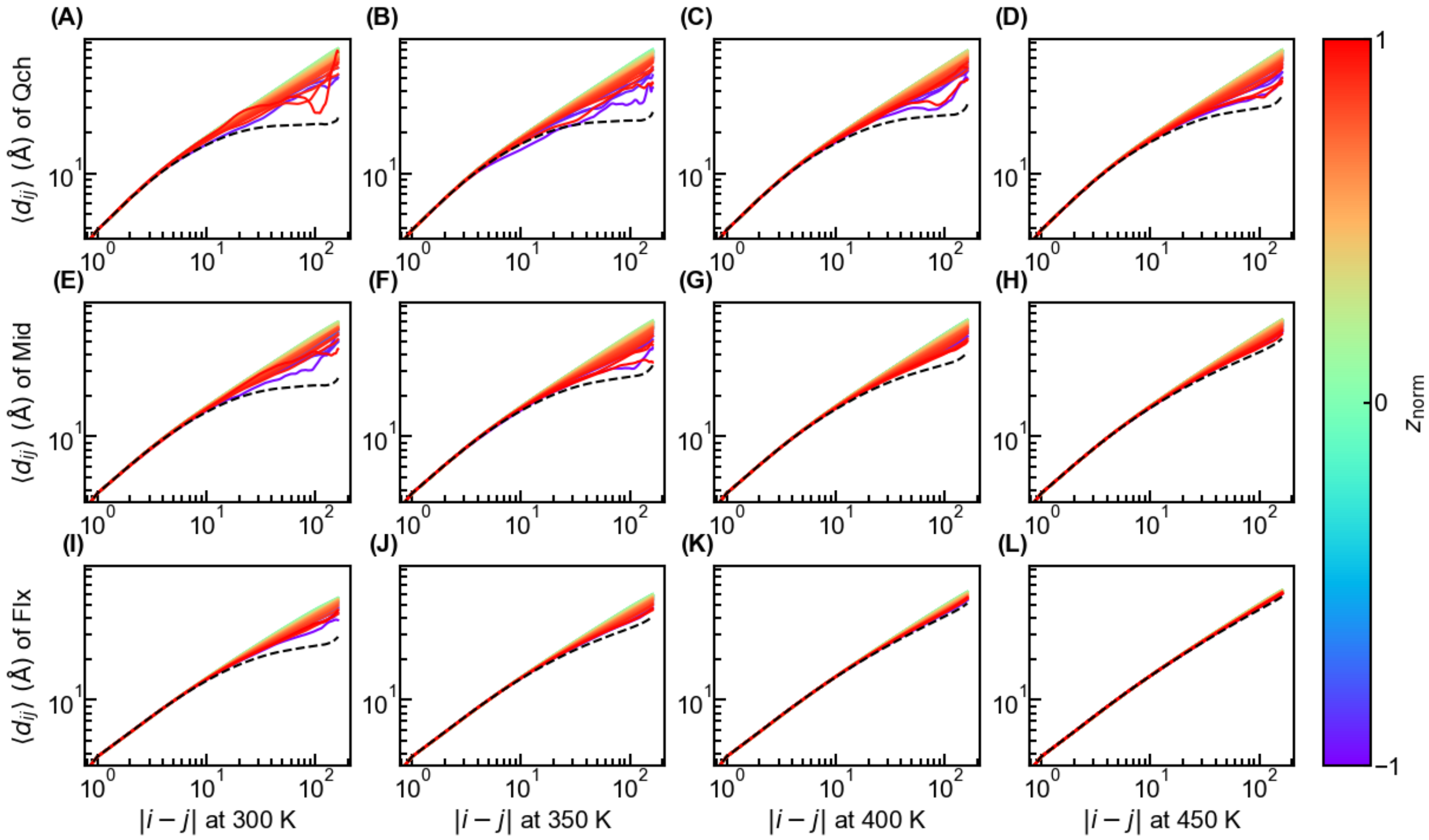

Figure S24: Position-resolved intrachain residue-pair distances in slab simulations. Rows correspond to Qch, Mid, and Flx; columns correspond to 300, 350, 400, and 450 K. Colored curves show the mean distance $\langle d_{ij} \rangle$ as a function of sequence separation $|i - j|$ for different normalized slab positions z_{norm} inside the dense slab, and black dashed curves show the corresponding isolated-chain reference at the same temperature.

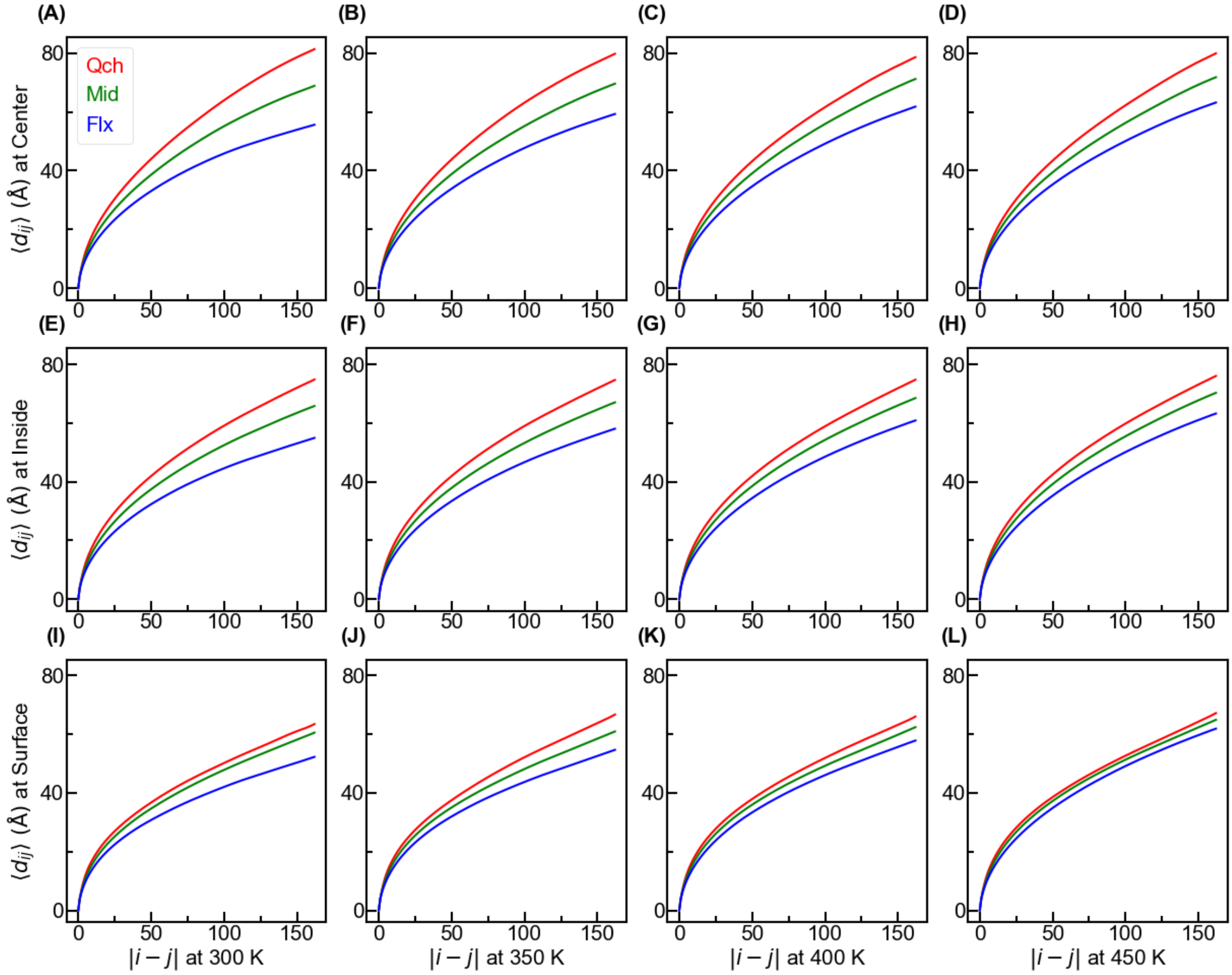

Figure S25: Model comparison of intrachain residue-pair distances at fixed temperature. Columns correspond to 300, 350, 400, and 450 K. Rows show mean distance $\langle d_{ij} \rangle$ as a function of sequence separation $|i - j|$ at the slab center, inside the condensed phase, and at the surface.

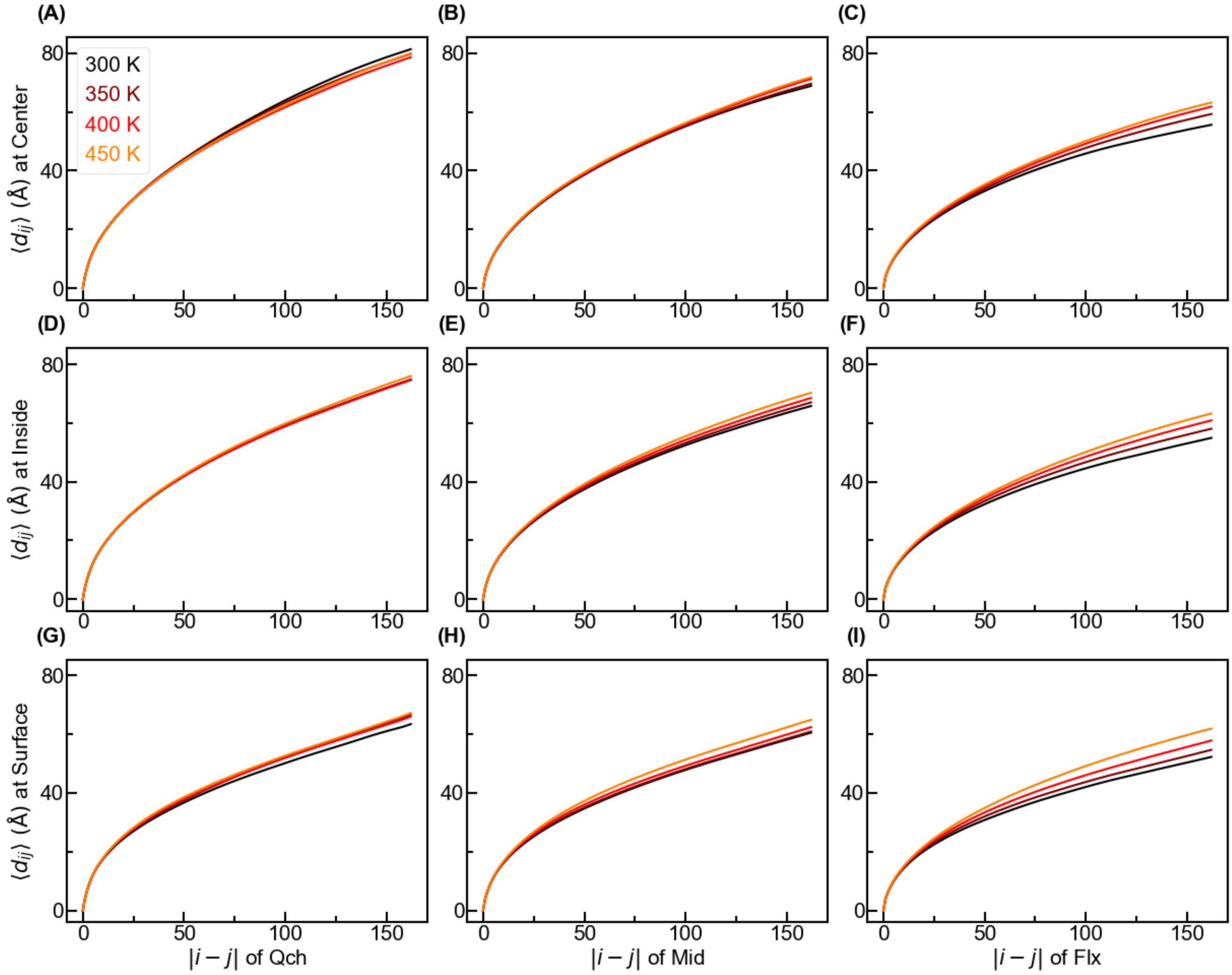

Figure S26: Temperature comparison of intrachain residue-pair distances within each model. Columns correspond to Qch, Mid, and Flx. Rows show mean distance $\langle d_{ij} \rangle$ as a function of sequence separation $|i - j|$ at the slab center, inside the condensed phase, and at the surface.

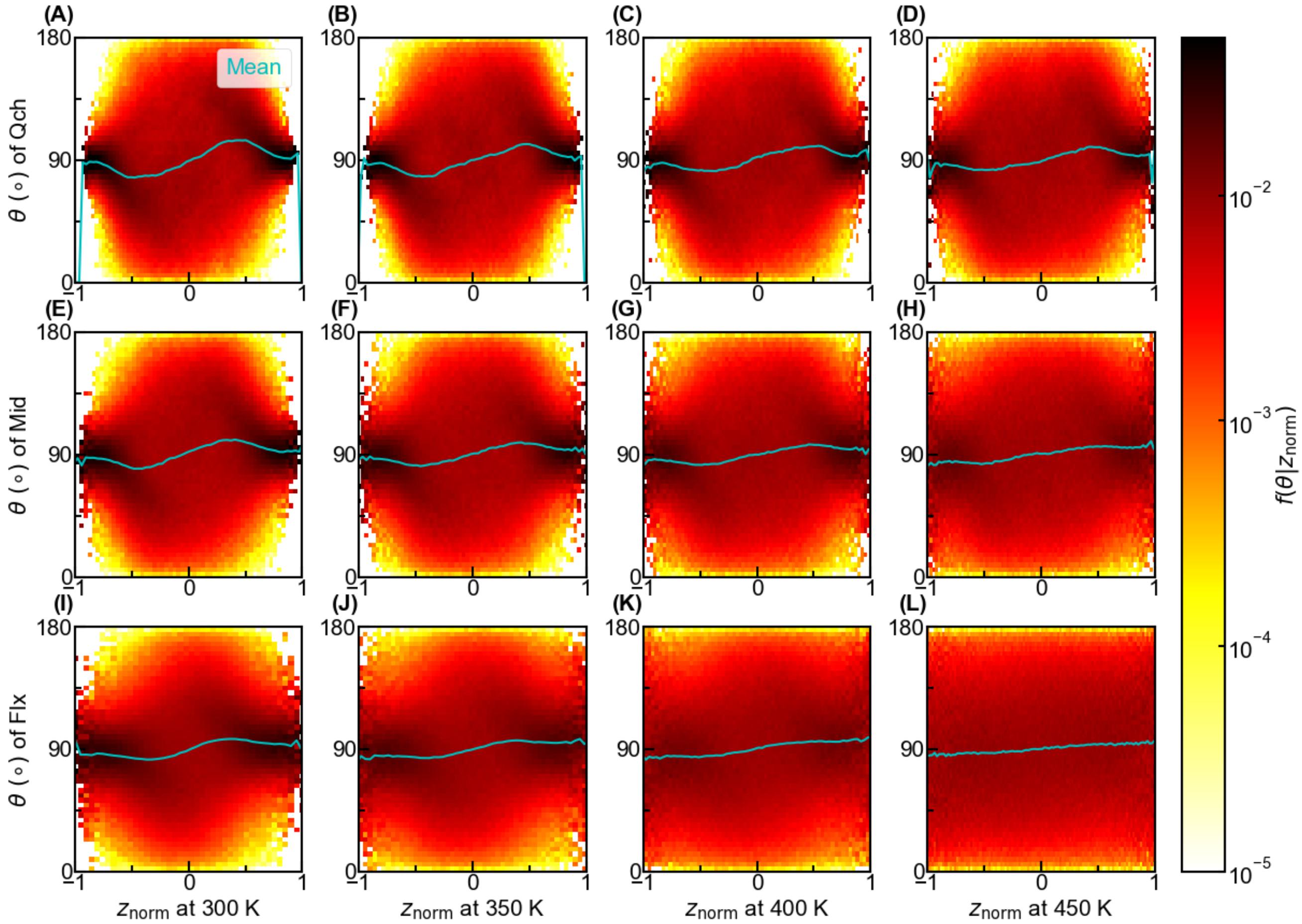

Figure S27: Position-resolved chain polar-angle distributions. Rows correspond to Qch, Mid, and Flx; columns correspond to 300, 350, 400, and 450 K. Heat maps show the probability density $f(\theta|z_{\text{norm}})$ at specific z_{norm}, where θ is the angle between the chain orientation axis and the slab-normal direction. Cyan curves indicate the local mean angle.

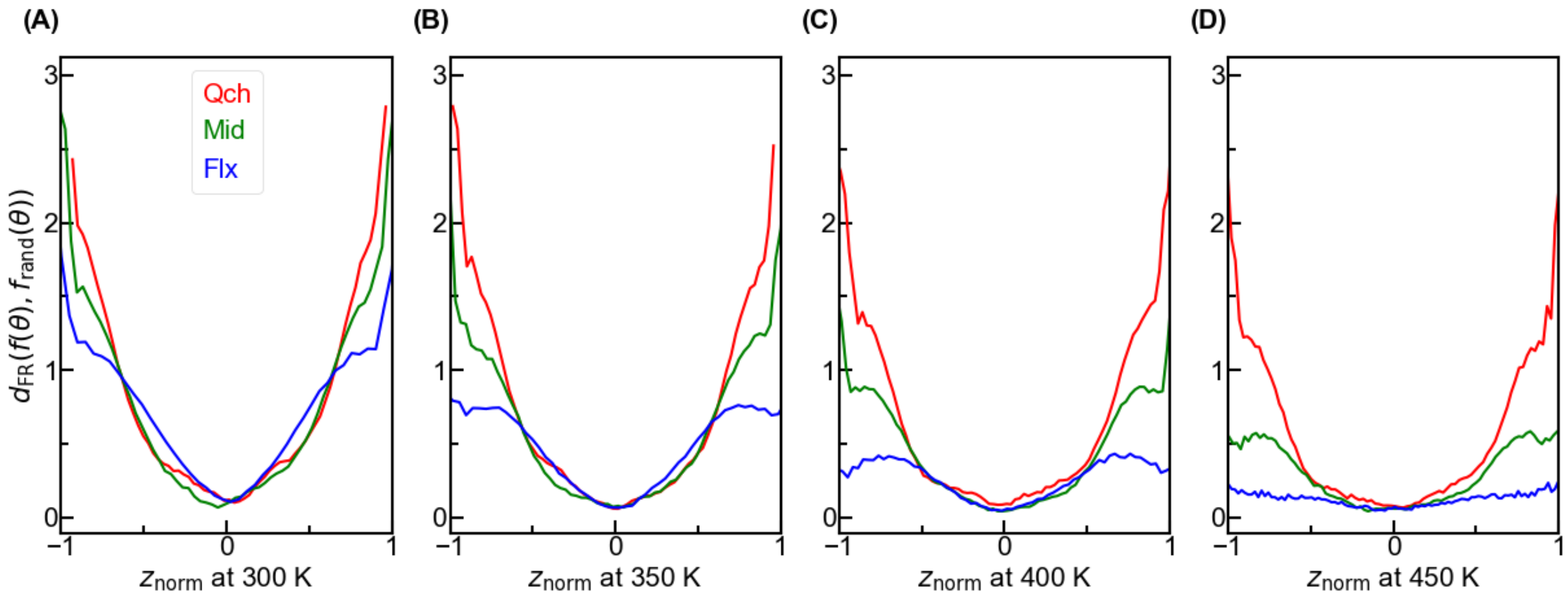

Figure S28: Position-resolved model comparison of deviations from random chain orientation at fixed temperature. Columns correspond to 300, 350, 400, and 450 K. Curves show the Fisher–Rao distance $d_{FR}(f(\theta), f_{rand}(\theta))$ as a function of z_{norm} for Qch, Mid, and Flx.

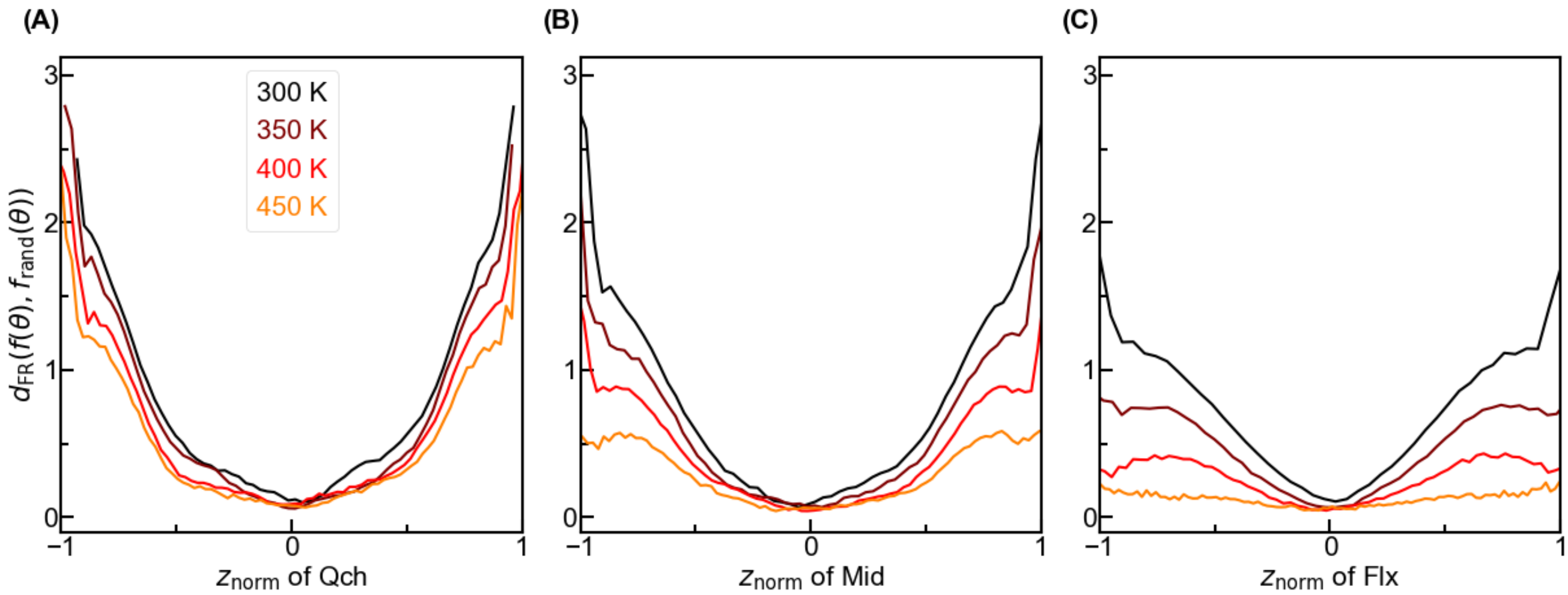

Figure S29: Position-resolved temperature comparison of deviations from random chain orientation within each model. Columns correspond to Qch, Mid, and Flx. Curves show the Fisher–Rao distance $d_{FR}(f(\theta), f_{rand}(\theta))$ as a function of z_{norm} at 300, 350, 400, and 450 K.

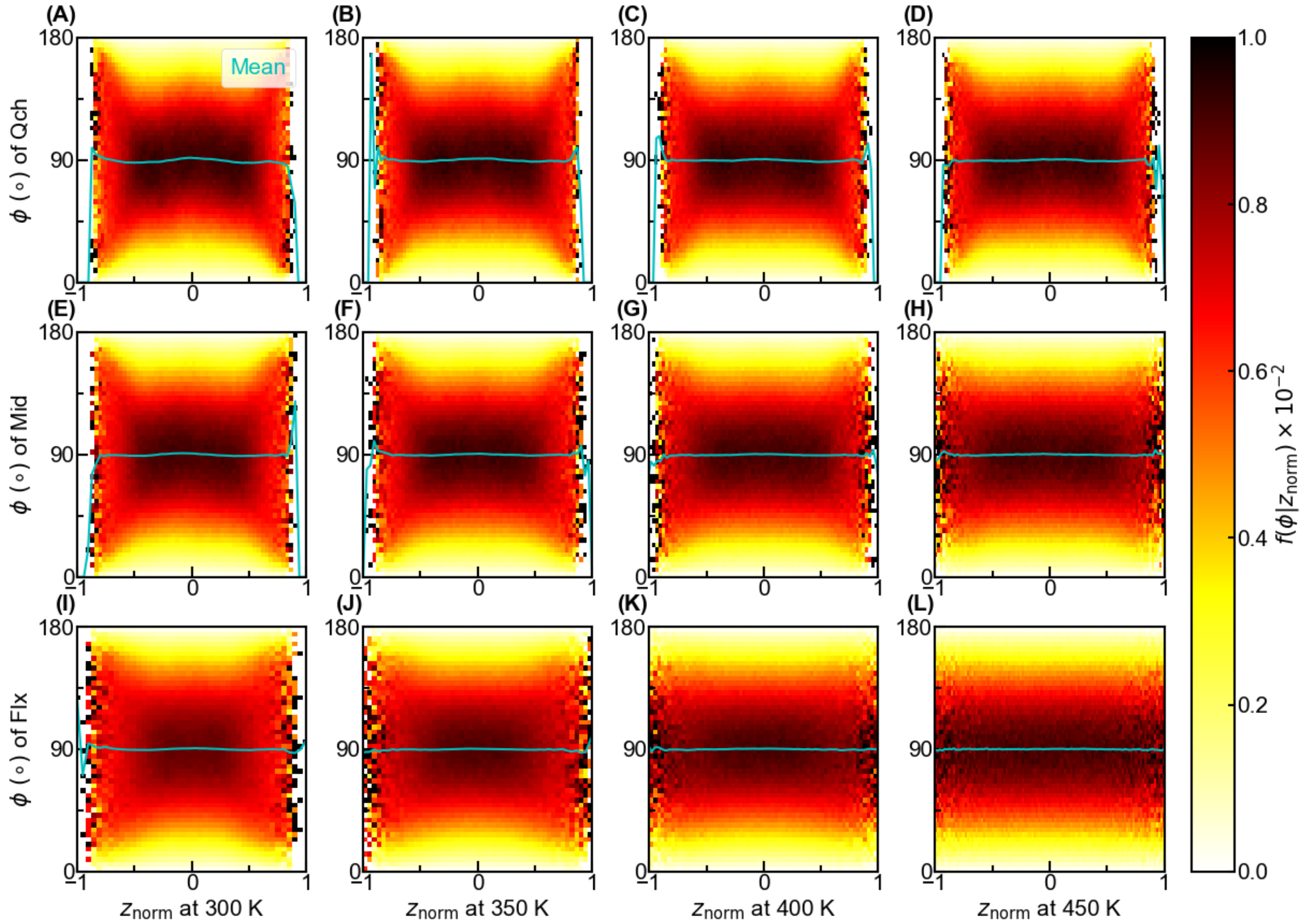

Figure S30: Position-resolved relative-orientation distributions of contacted chains. Rows correspond to Qch, Mid, and Flx; columns correspond to 300, 350, 400, and 450 K. Heat maps show the probability density $f(\phi|z_{\text{norm}})$ at specific z_{norm}, where ϕ is the angle between orientation axes of contacted chain pairs. Cyan curves indicate the local mean relative angle.

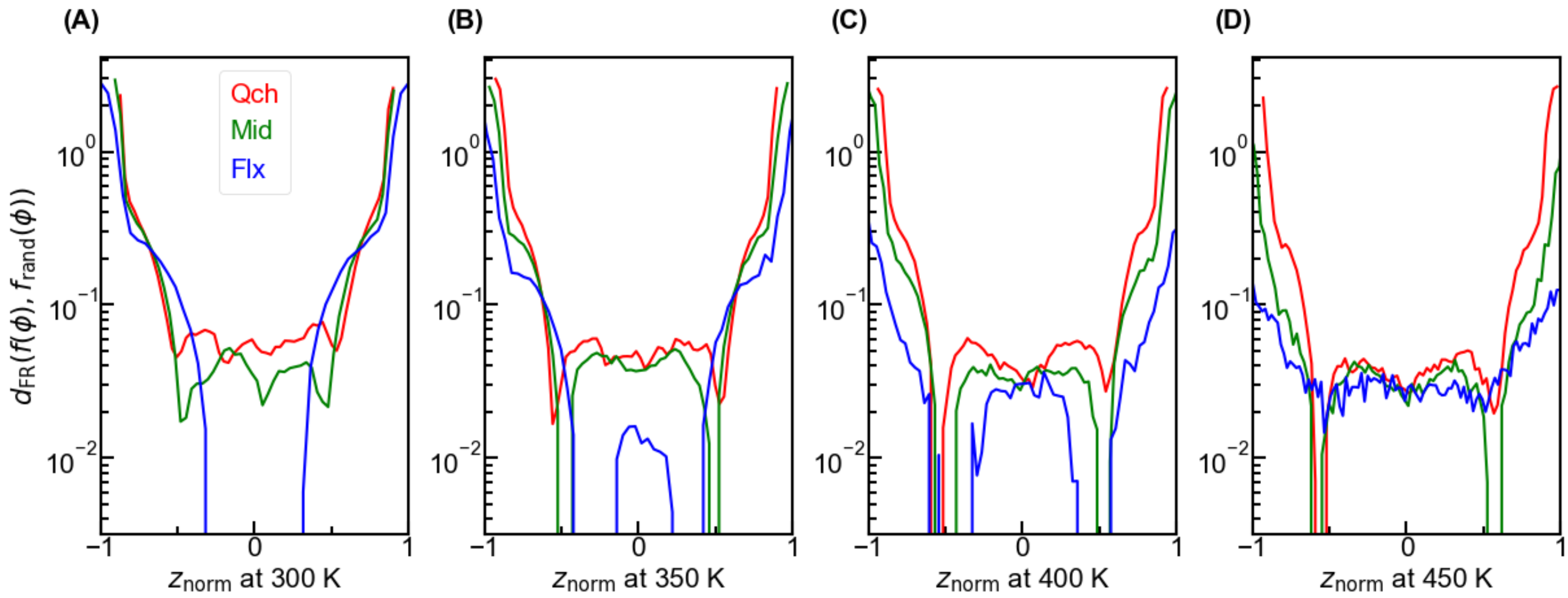

Figure S31: Position-resolved model comparison of deviations from random relative orientation at fixed temperature. Columns correspond to 300, 350, 400, and 450 K. Curves show the Fisher–Rao distance $d_{\mathrm{FR}}(f(\phi), f_{\mathrm{rand}}(\phi))$ as a function of z_{norm} for Qch, Mid, and Flx.

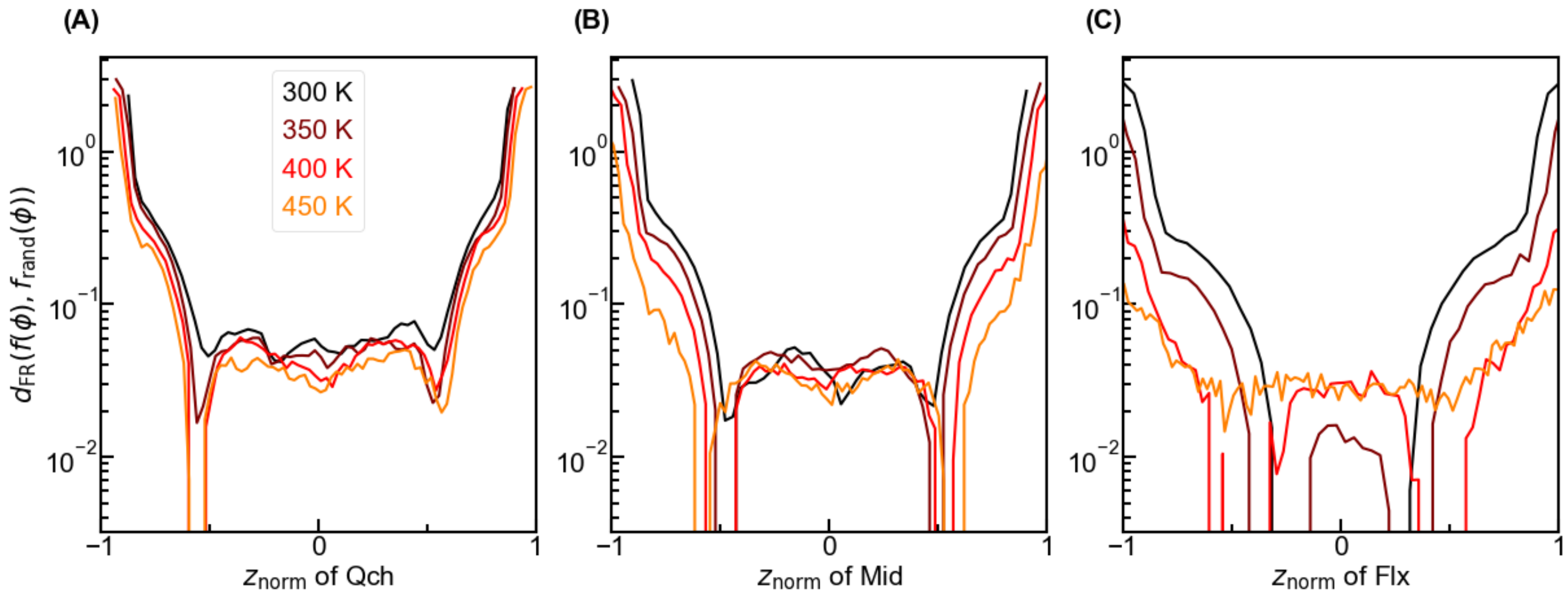

Figure S32: Position-resolved temperature comparison of deviations from random relative orientation within each model. Columns correspond to Qch, Mid, and Flx. Curves show the Fisher–Rao distance $d_{FR}(f(\phi), f_{rand}(\phi))$ as a function of z_{norm} at 300, 350, 400, and 450 K.

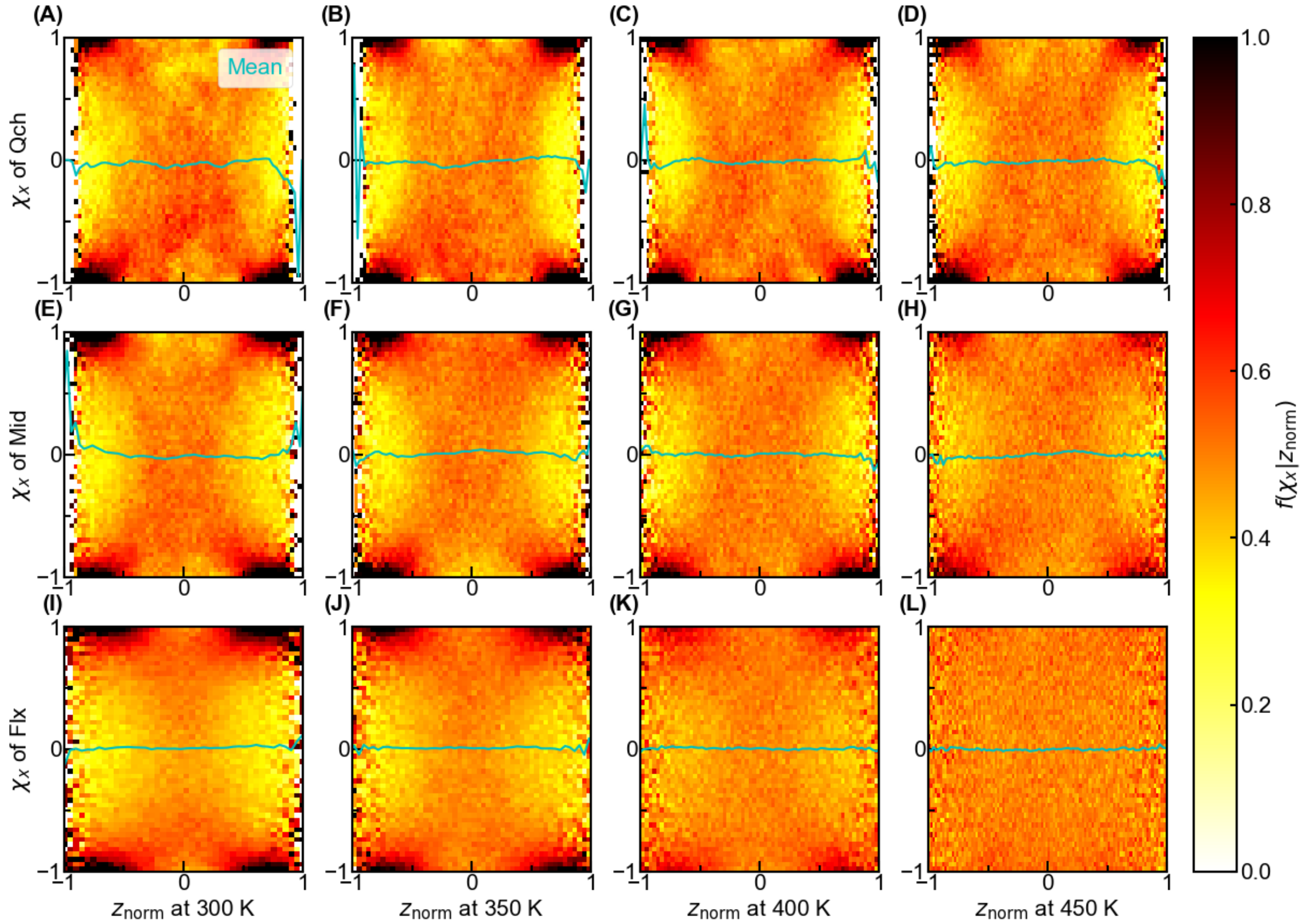

Figure S33: Position-resolved distribution of the x component of the local orientational polarization. Rows correspond to Qch, Mid, and Flx; columns correspond to 300, 350, 400, and 450 K. Heat maps show the probability density $f(\chi_x|z_{\mathrm{norm}})$ at specific z_{norm}, where χ_x is the x component of the local chain-orientation polarization. Cyan curves indicate the local mean.

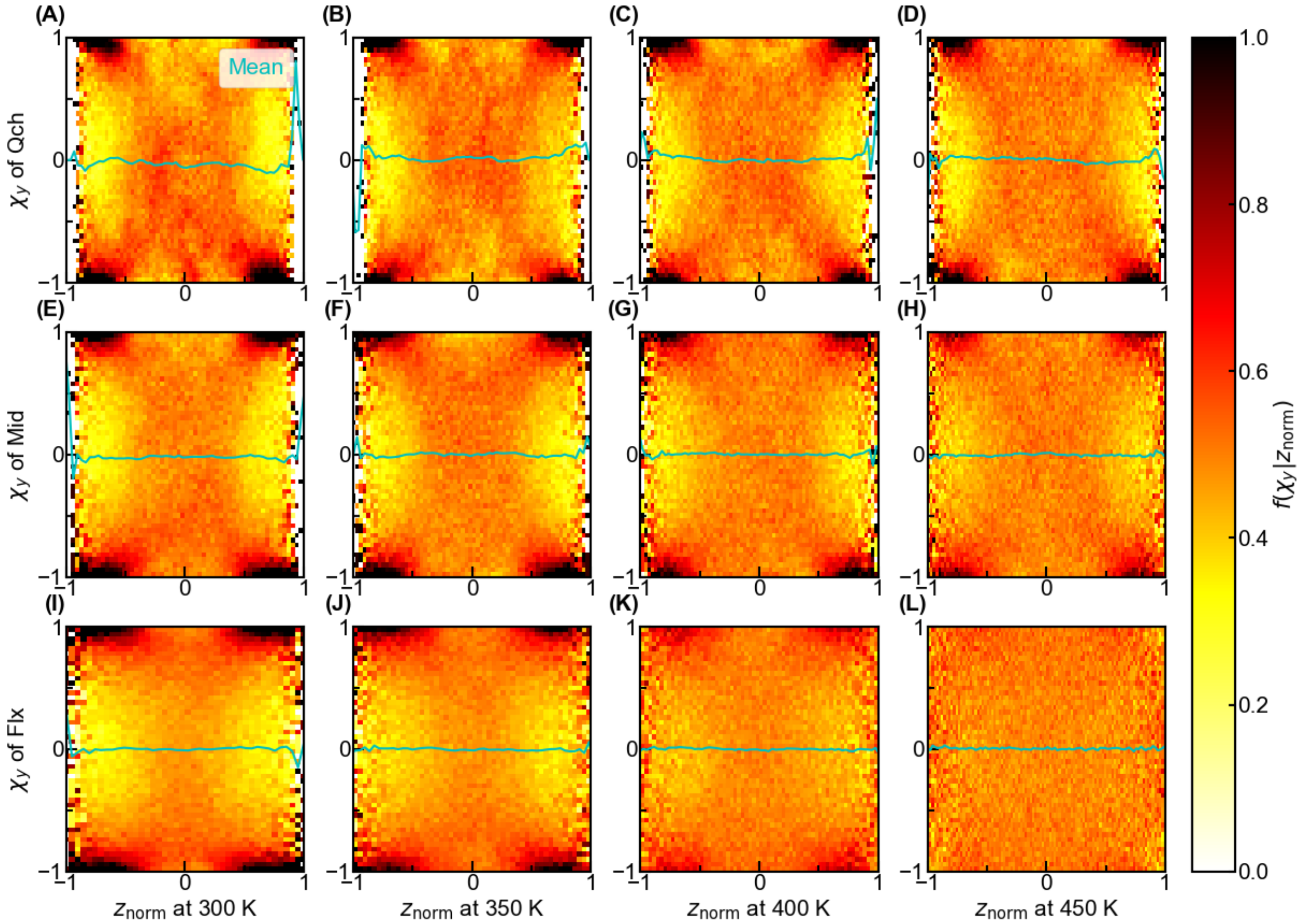

Figure S34: Position-resolved distribution of the y component of the local orientational polarization. Rows correspond to Qch, Mid, and Flx; columns correspond to 300, 350, 400, and 450 K. Heat maps show the probability density $f(\chi_y|z_{\mathrm{norm}})$ at specific z_{norm}, where χ_y is the y component of the local chain-orientation polarization. Cyan curves indicate the local mean.

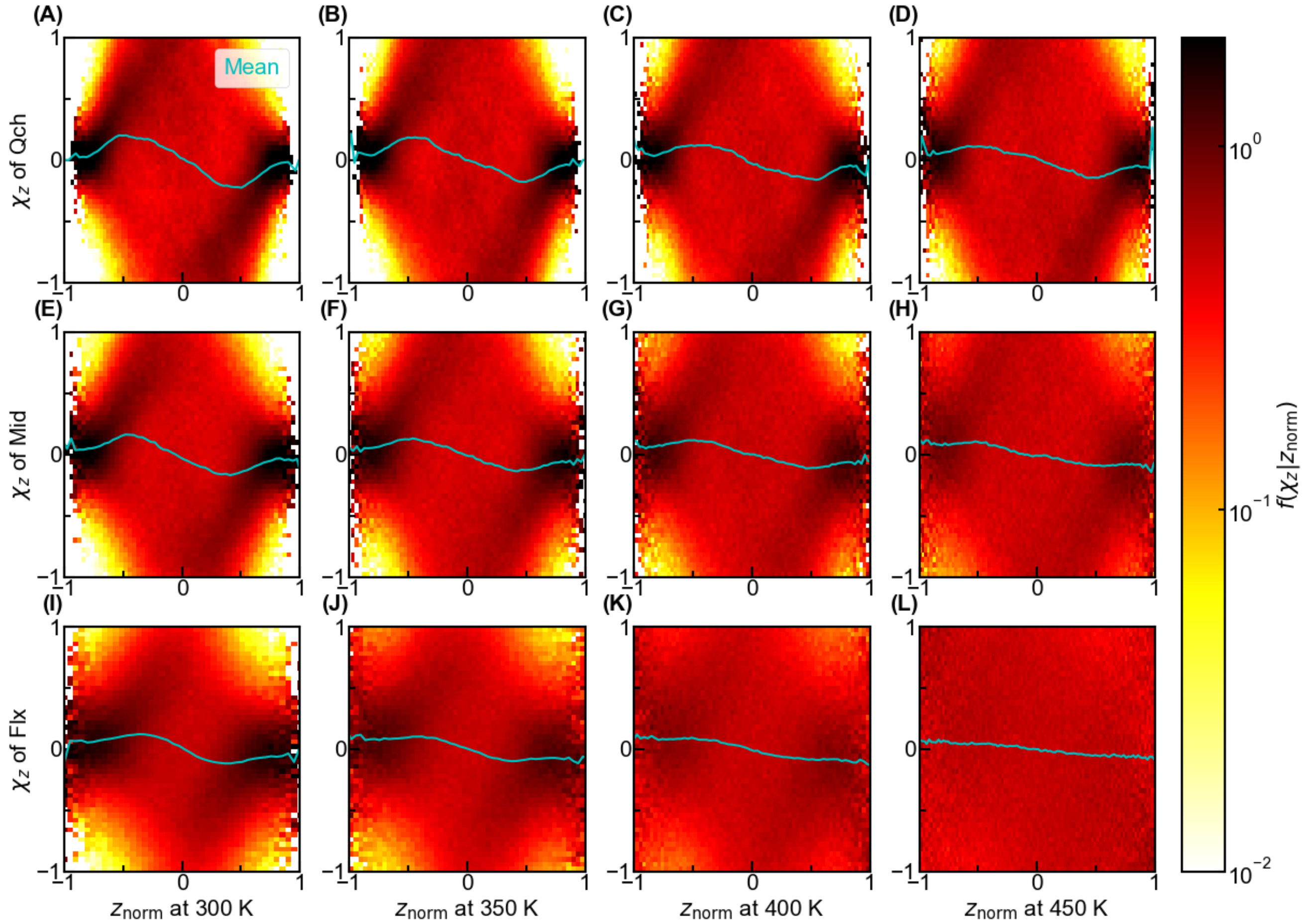

Figure S35: Position-resolved distribution of the z component of the local orientational polarization. Rows correspond to Qch, Mid, and Flx; columns correspond to 300, 350, 400, and 450 K. Heat maps show the probability density $f(\chi_z|z_{\text{norm}})$ at specific z_{norm}, where χ_z is the z component of the local chain-orientation polarization. Cyan curves indicate the local mean.

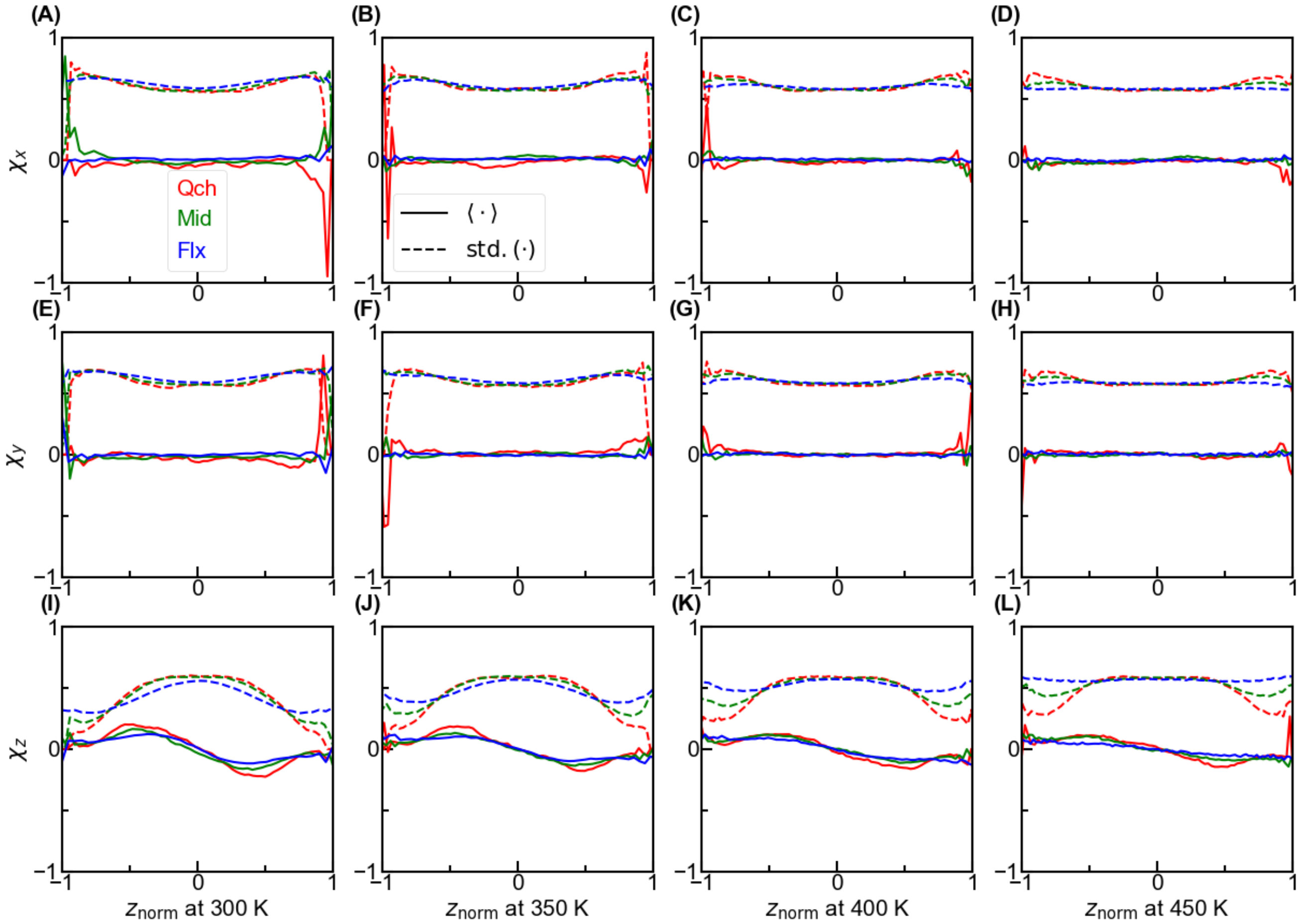

Figure S36: Position-resolved model comparison of local orientational polarization components at fixed temperature. Rows correspond to χ_x, χ_y, and χ_z, and columns correspond to 300, 350, 400, and 450 K. Curves compare Qch, Mid, and Flx. Solid and dashed curves denote the local mean $\langle \chi_\alpha \rangle$ and standard deviation std.(χ_α), respectively, where $\alpha = x, y, z$.

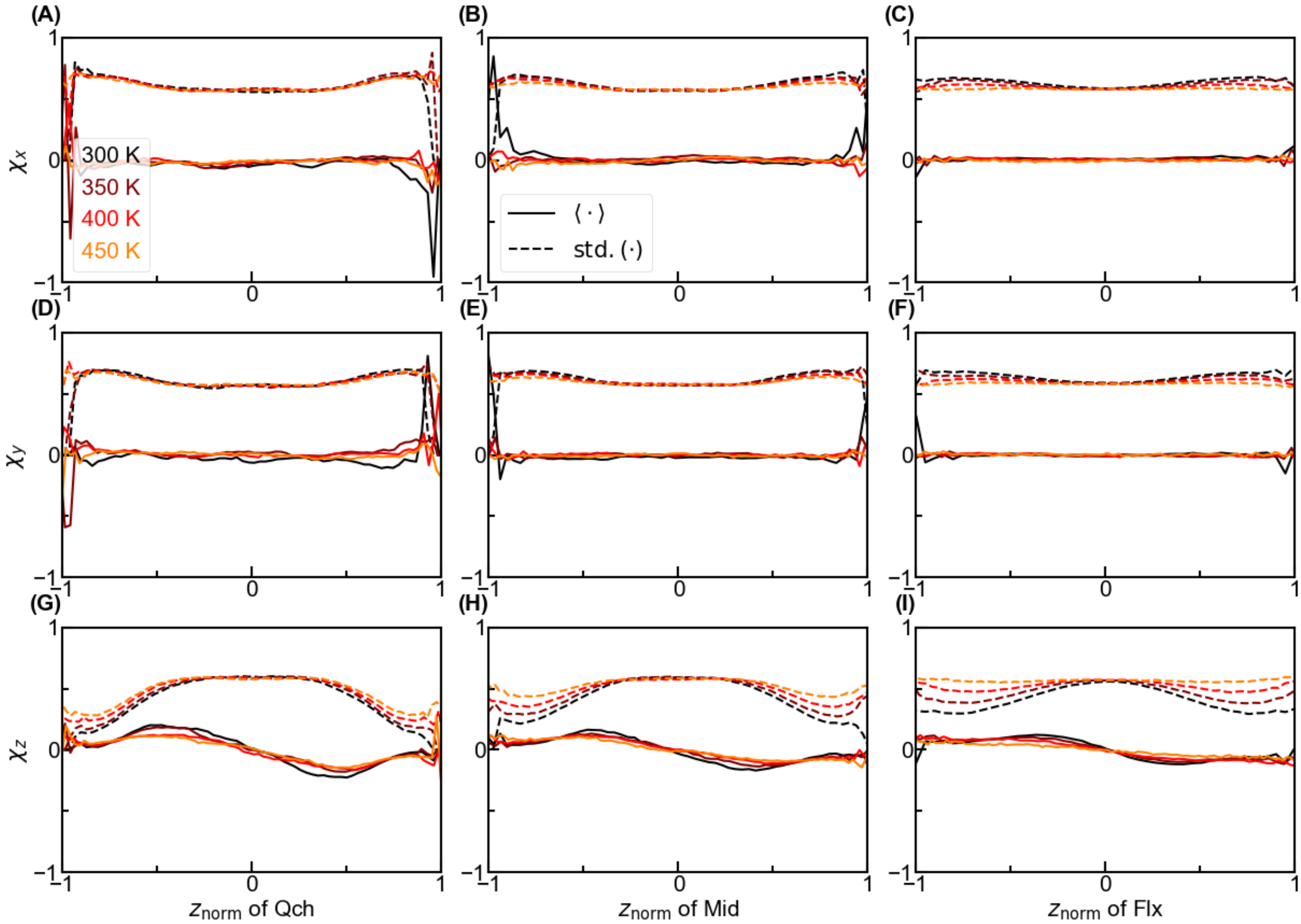

Figure S37: Position-resolved temperature comparison of local orientational polarization components within each model. Rows correspond to χ_x, χ_y, and χ_z, and columns correspond to Qch, Mid, and Flx. Curves compare 300, 350, 400, and 450 K. Solid and dashed curves denote the local mean $\langle \chi_\alpha \rangle$ and standard deviation std.(χ_α), respectively, where $\alpha = x, y, z$.

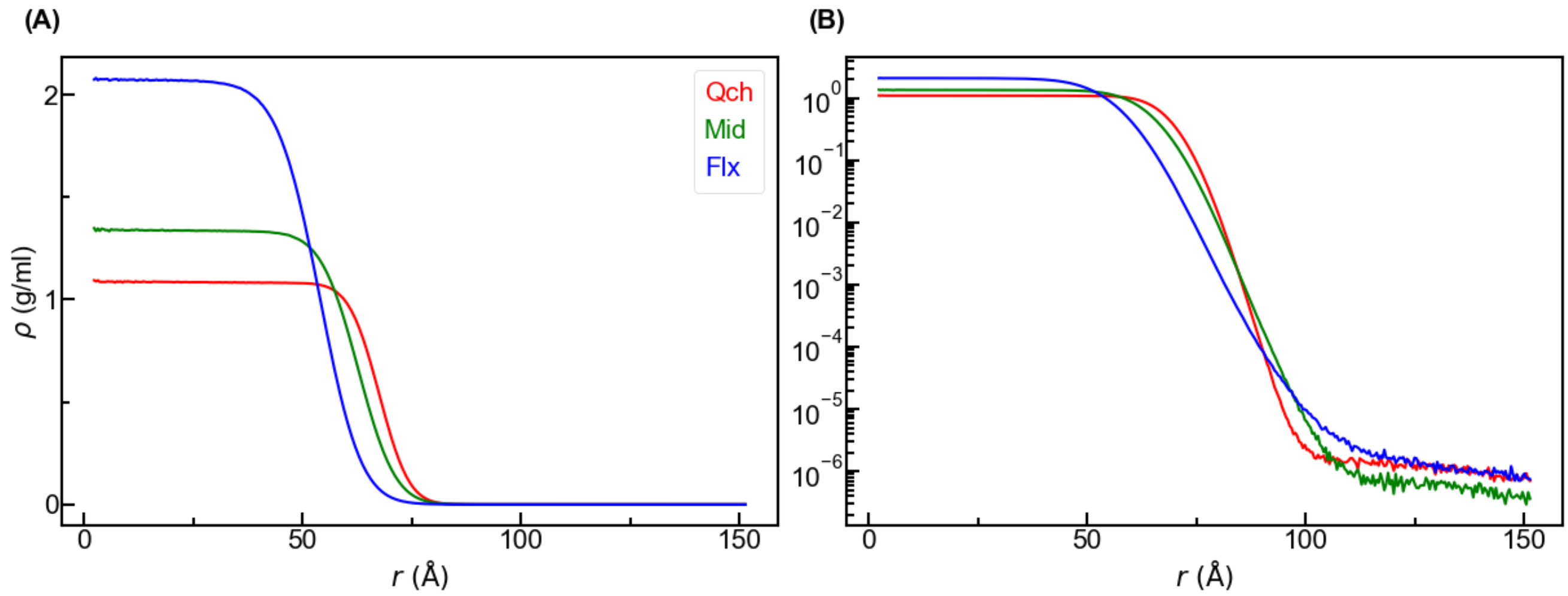

Figure S38: Radial density distribution $\rho(r)$ of a spherical condensate shown on (A) linear and (B) logarithmic scales.

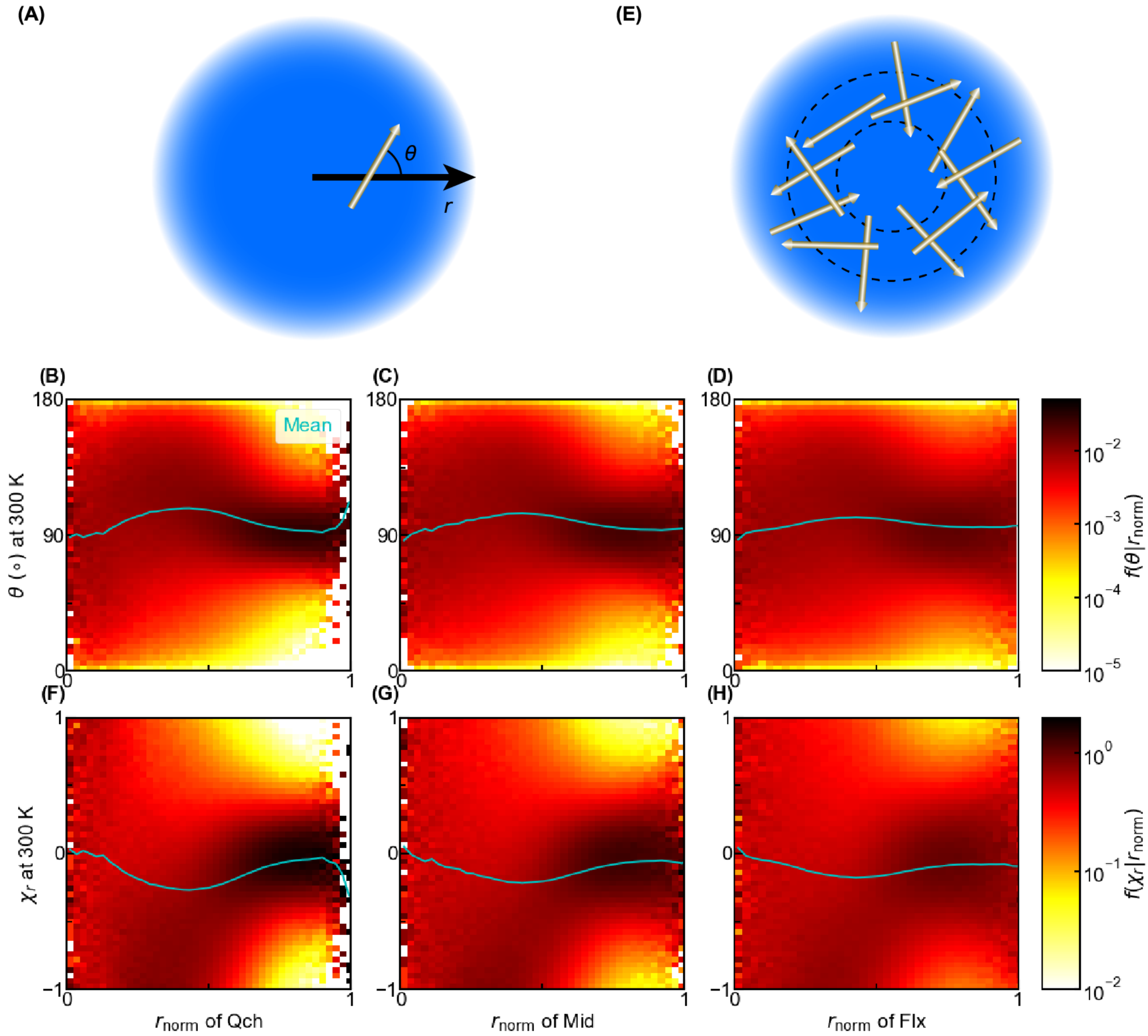

Figure S39: Position-resolved radial orientational distributions at 300 K. (A) Definition of the orientation angle θ between the chain vector and the radial r direction. The arrow denotes the orientation unit vector along the first principal axis of a chain, directed from the N-terminus to the C-terminus. The blue region denotes the condensed phase. (B-D) The probability density $f(\theta|r_{\text{norm}})$ of the chain polar angle θ at specific r_{norm}, for Qch, Mid, and Flx respectively. (E) Definition of local orientational polarization. For a position r and bin width dr, the distribution of components of chain-orientation vectors $\boldsymbol{\chi}(r)$ whose centers of mass lie within the bin represents the polarization. (F-H) The probability density $f(\chi_r|r_{\text{norm}})$ of the radial component χ_r at specific r_{norm}. Cyan curves indicate the corresponding local means.

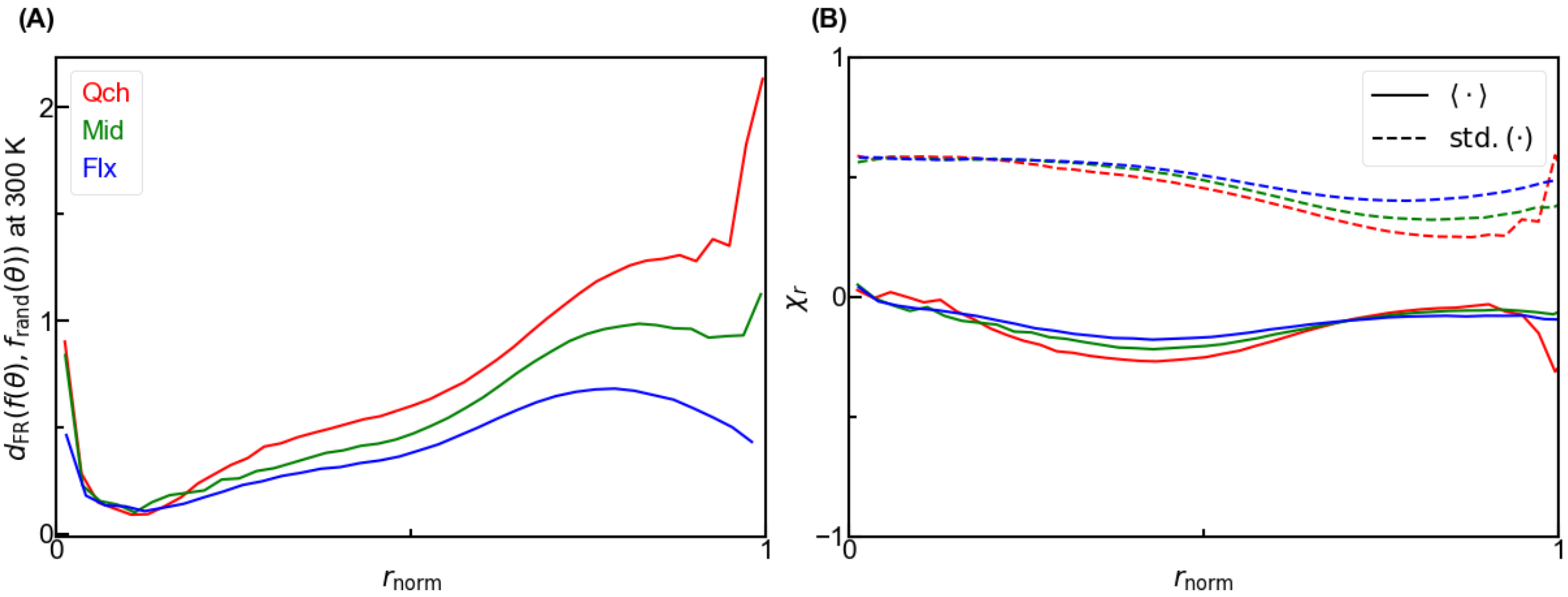

Figure S40: Position-resolved radial orientational properties at 300 K. The left panel shows the Fisher–Rao distance $d_{\mathrm{FR}}(f(\theta), f_{\mathrm{rand}}(\theta))$ from the random-orientation distribution as a function of r_{norm} for Qch, Mid, and Flx. The right panel shows the radial component χ_r of the local orientational polarization, with solid and dashed curves denoting the local mean $\langle \chi_r \rangle$ and standard deviation std.(χ_r), respectively.

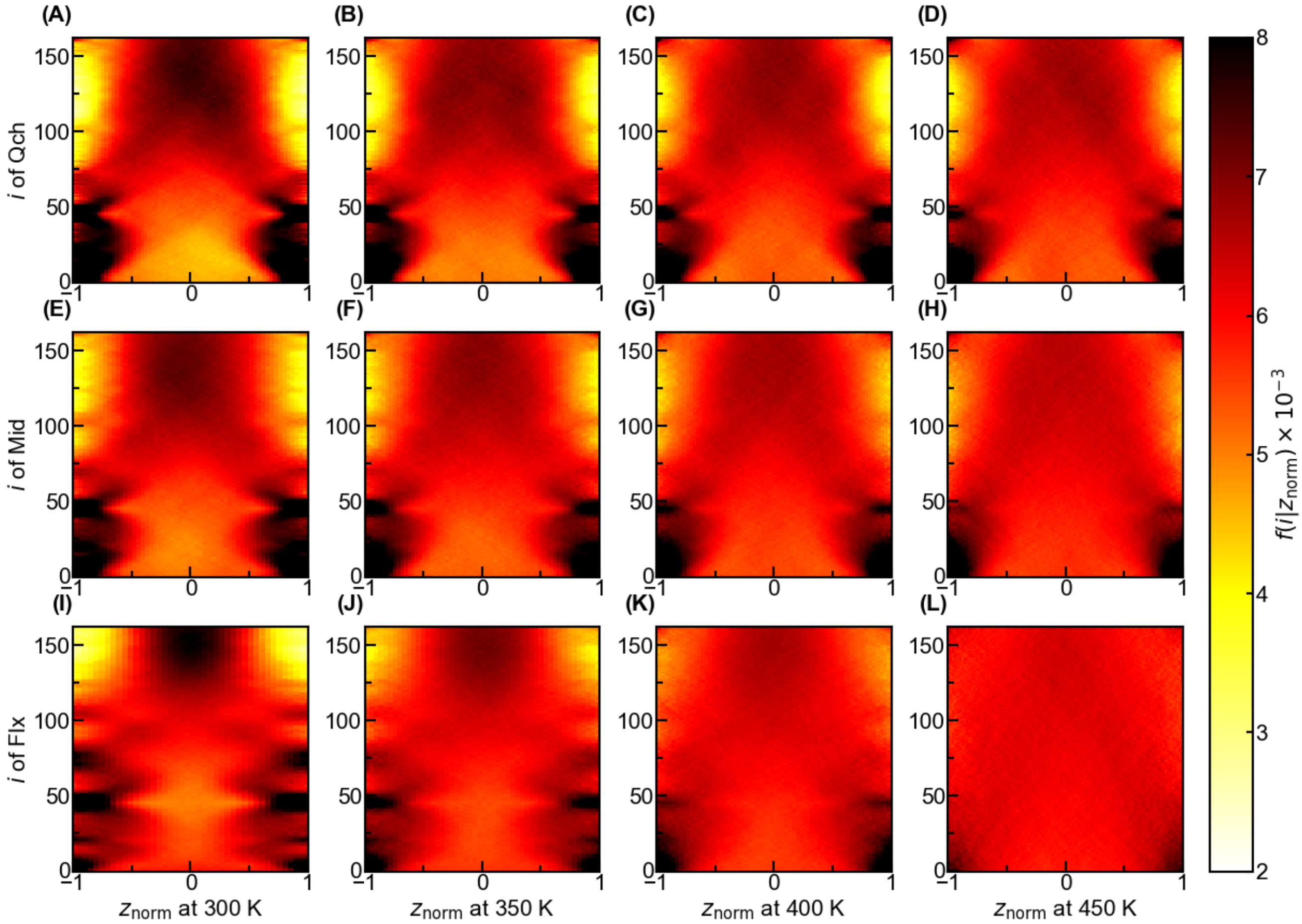

Figure S41: Residue-resolved localization within slab simulations. Rows correspond to Qch, Mid, and Flx; columns correspond to 300, 350, 400, and 450 K. Each column of each heat map reports the probability for residue index i at the specific normalized slab coordinate z_{norm}.

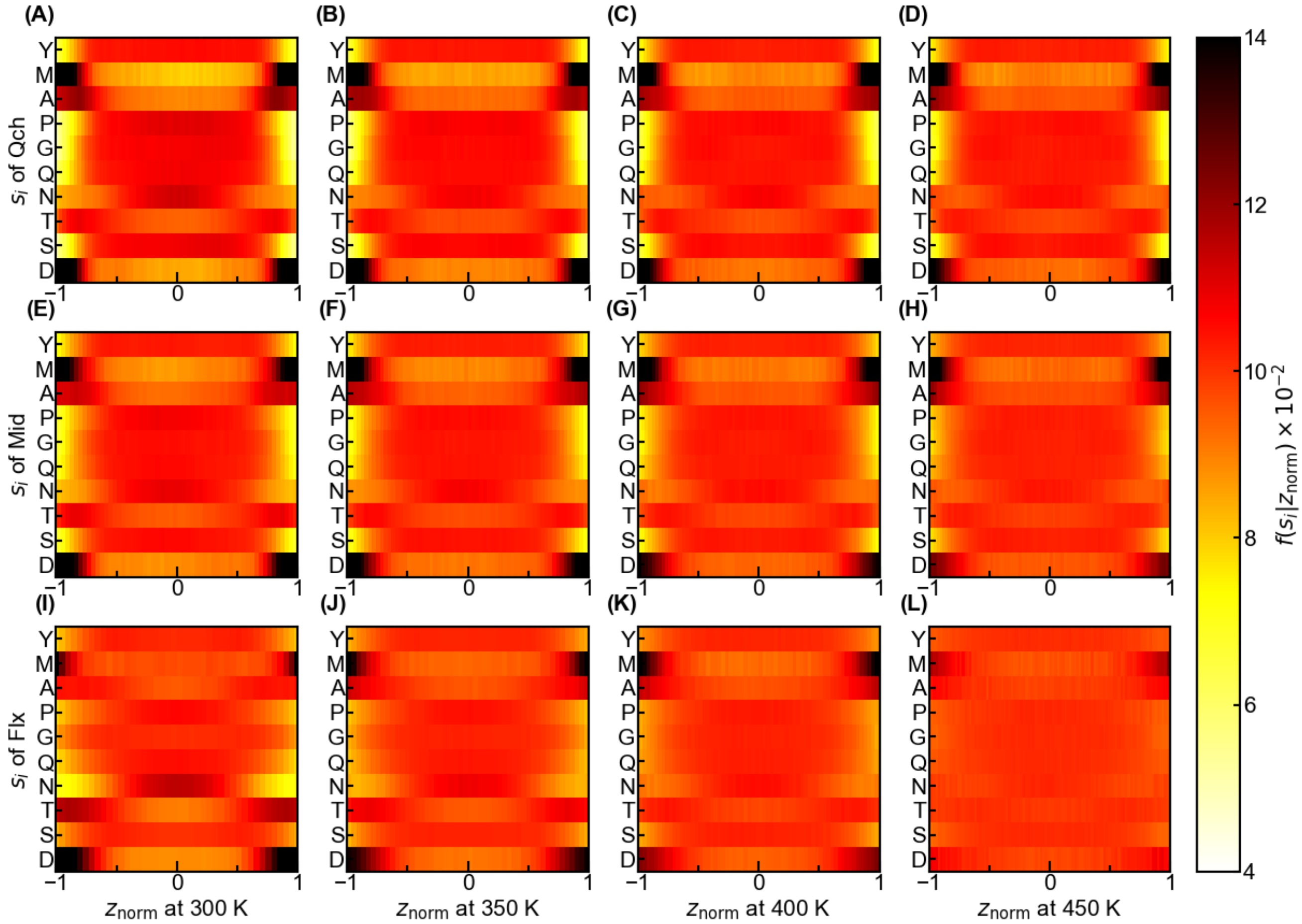

Figure S42: Residue-type-resolved localization within slab simulations. Rows correspond to Qch, Mid, and Flx; columns correspond to 300, 350, 400, and 450 K. Each column of each heat map reports the probability at specific z_{norm} for residue types ordered by physicochemical class.

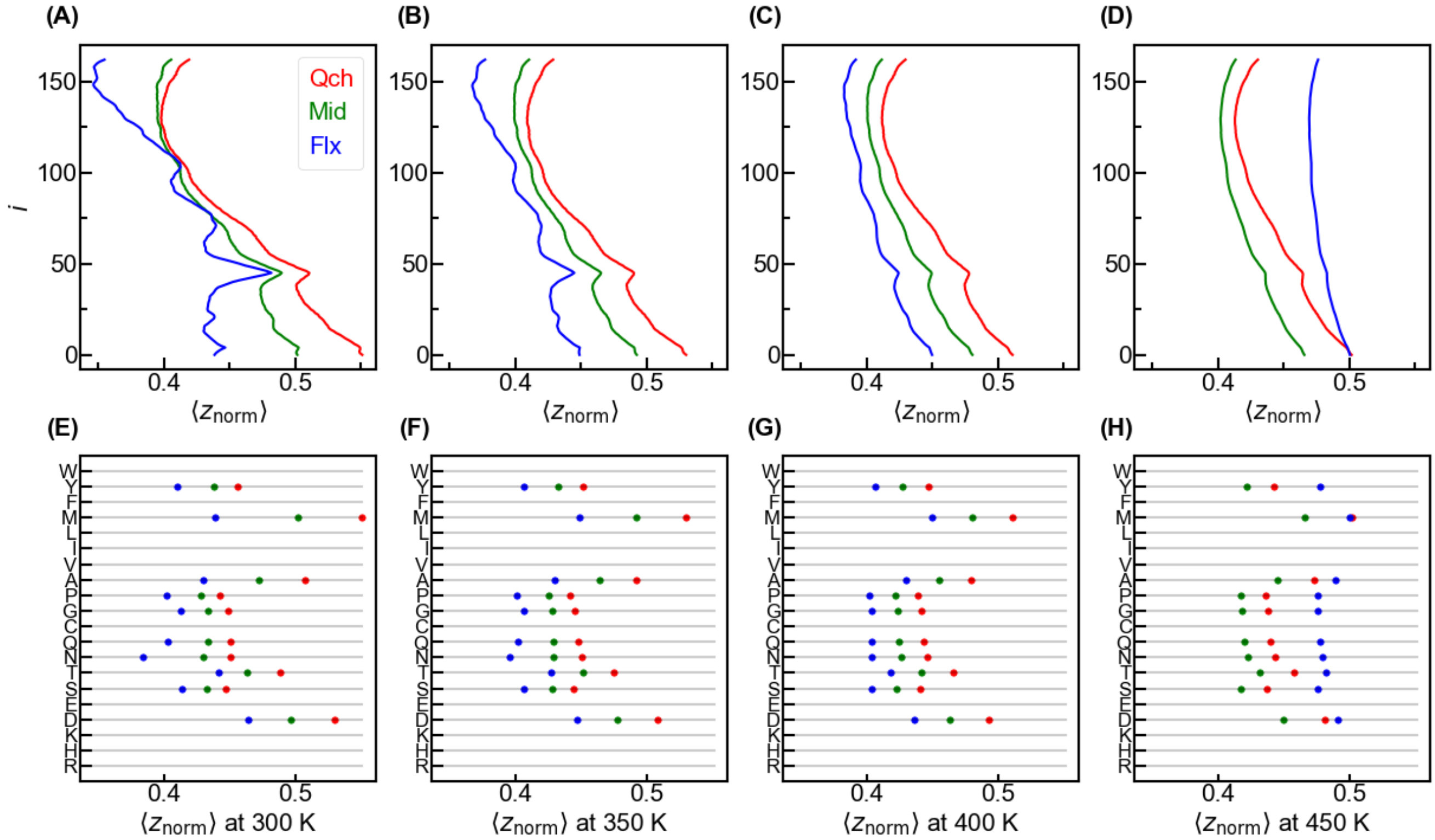

Figure S43: Model comparison of mean residue localization at fixed temperature. (A–D) Sequence profiles of the mean absolute normalized position $\langle|z_{\mathrm{norm}}|\rangle$ for each residue at 300, 350, 400, and 450 K. (E–H) Residue-type averaged $\langle|z_{\mathrm{norm}}|\rangle$ for the same temperatures, with residue types ordered by physicochemical class.

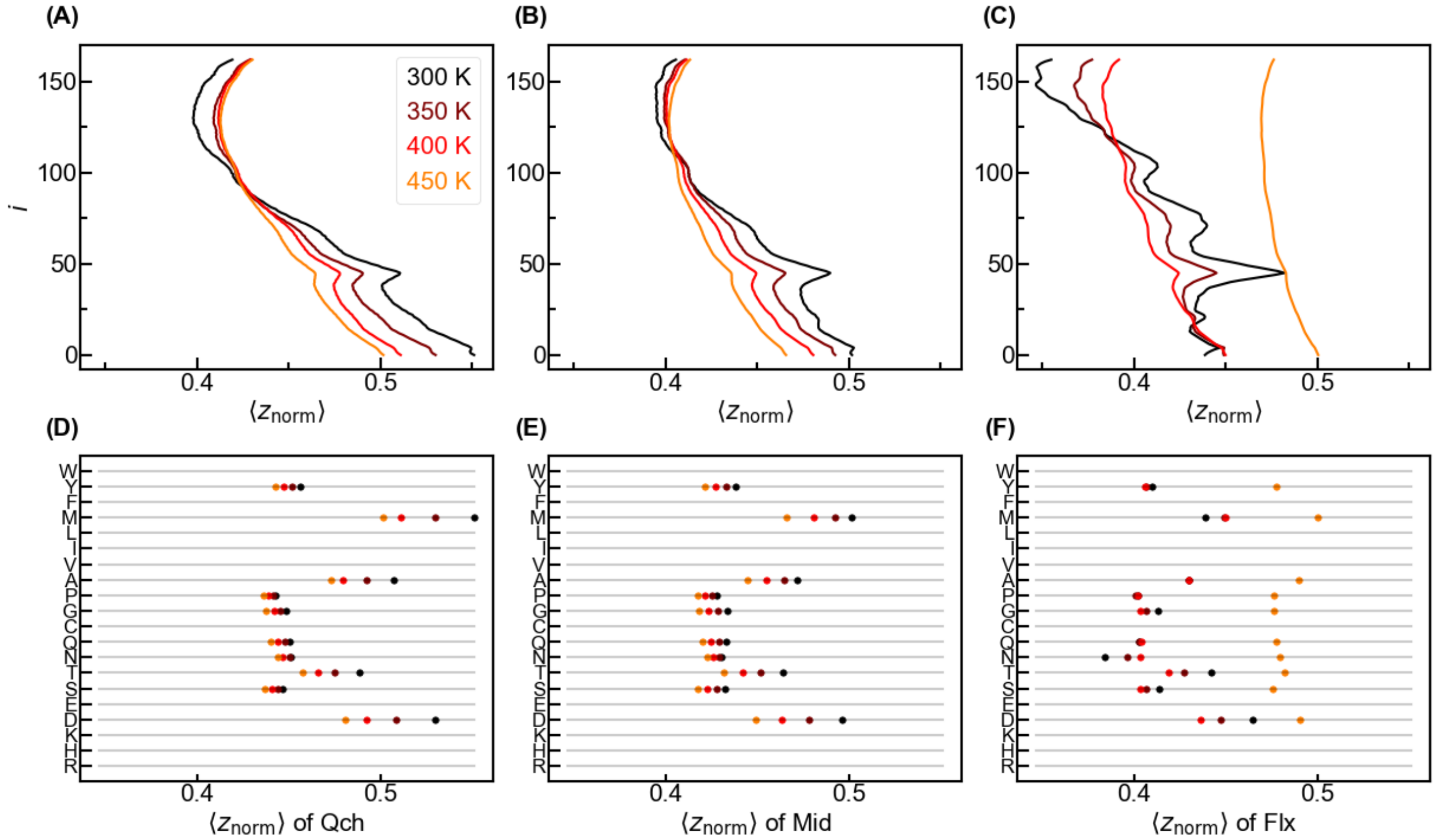

Figure S44: Temperature comparison of mean residue localization within each model. (A–C) Sequence profiles of the mean absolute normalized position $\langle|z_{\mathrm{norm}}|\rangle$ for Qch, Mid, and Flx. (D–F) Residue-type averaged $\langle|z_{\mathrm{norm}}|\rangle$ for the same models, with points colored by temperature and residue types ordered by physicochemical class.

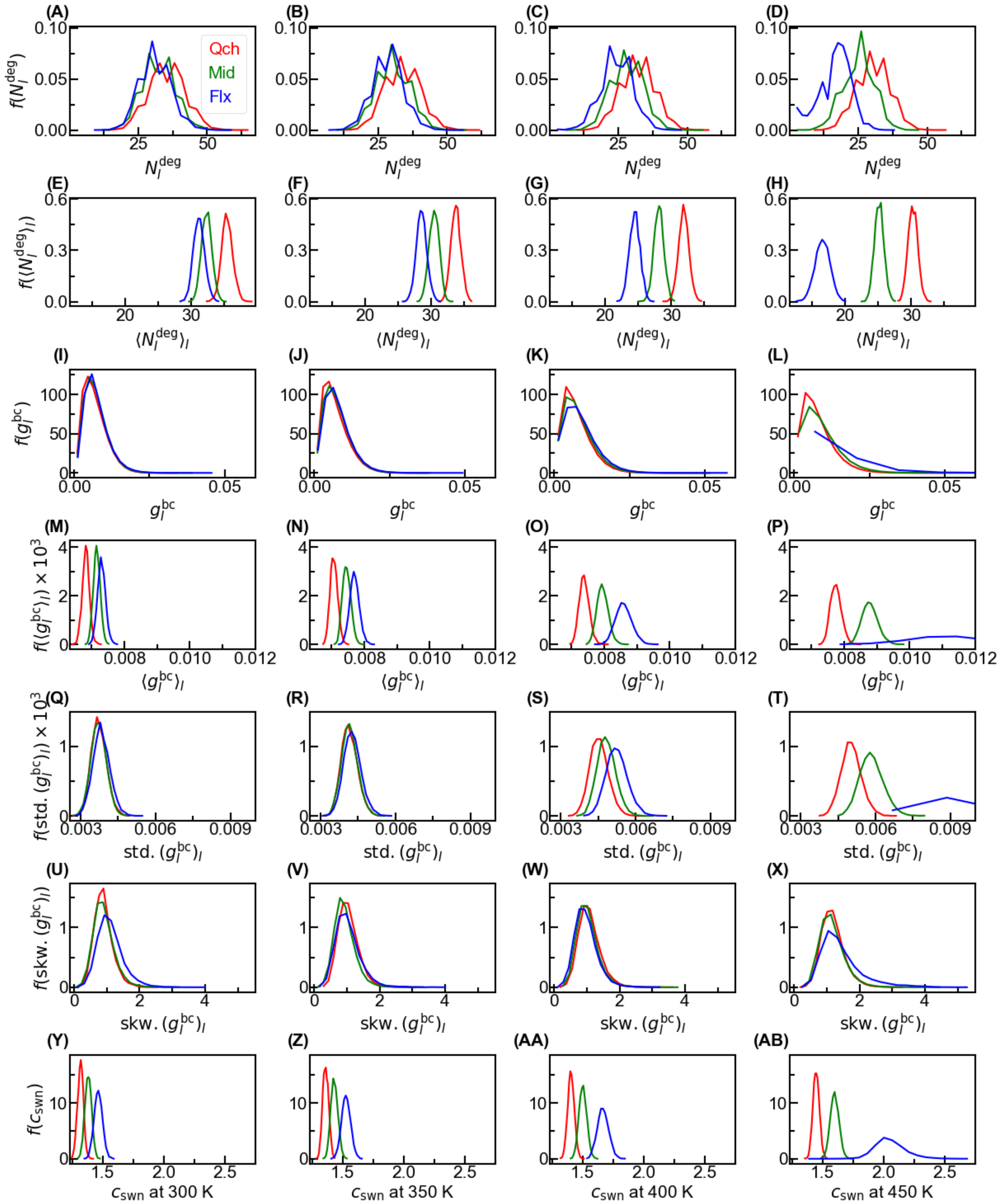

Figure S45: Model comparison of chain-contact network metrics at fixed temperature. Columns correspond to 300, 350, 400, and 450 K. Rows show probability distributions of node degree N_I^{deg}, system-level average degree $\langle N_I^{\text{deg}} \rangle_I$, node betweenness centrality g_I^{bc}, system-level average betweenness $\langle g_I^{\text{bc}} \rangle_I$, system-level standard deviation std.$(g_I^{\text{bc}})_I$, system-level skewness skw.$(g_I^{\text{bc}})_I$, and small-world coefficient c_{swn}. Curves compare Qch, Mid, and Flx.

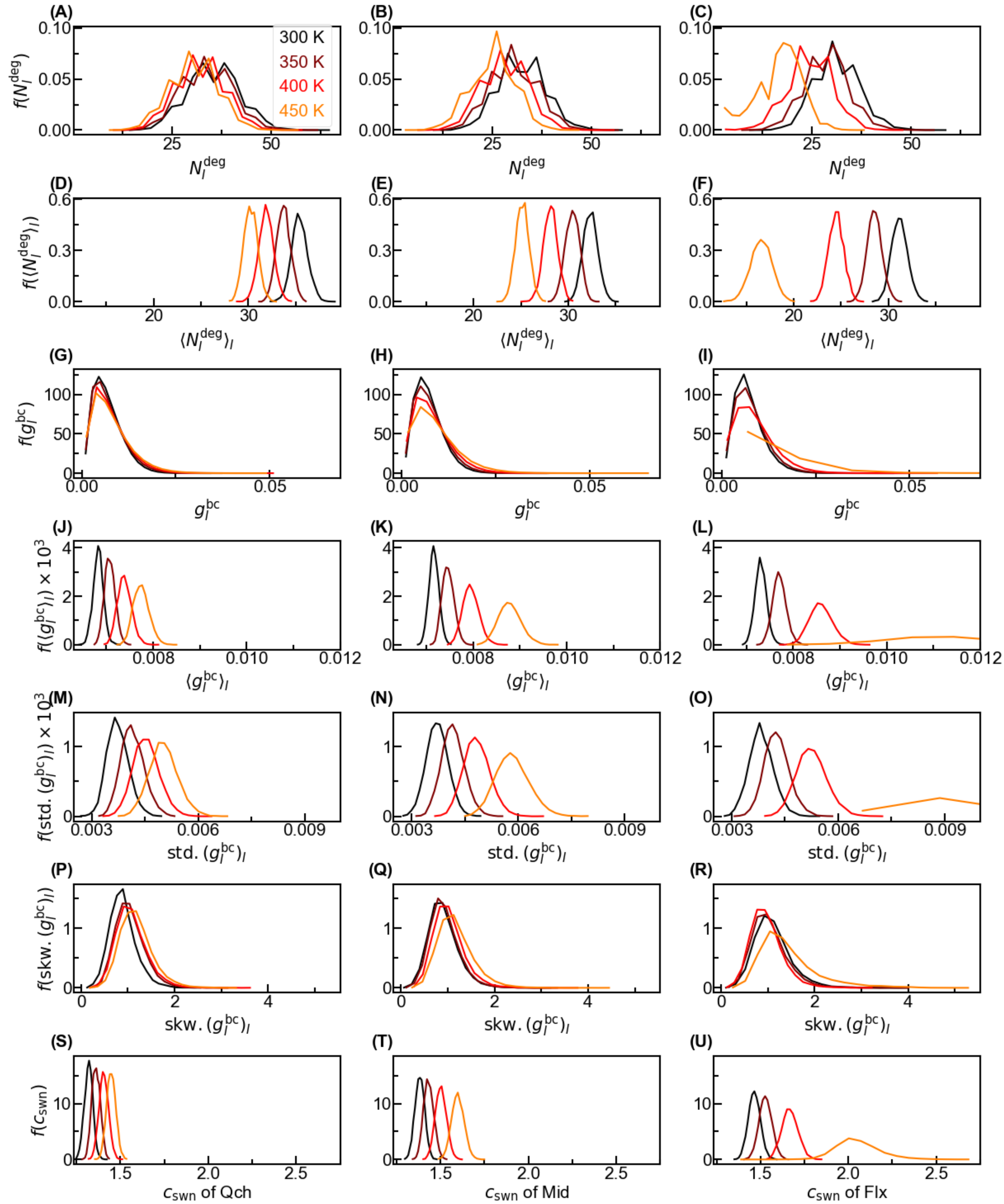

Figure S46: Temperature comparison of chain-contact network metrics within each model. Columns correspond to Qch, Mid, and Flx. Rows show probability distributions of node degree N_I^{deg}, system-level average degree $\langle N_I^{\mathrm{deg}}\rangle_I$, node betweenness centrality g_I^{bc}, system-level average betweenness $\langle g_I^{\mathrm{bc}}\rangle_I$, system-level standard deviation $\mathrm{std.}(g_I^{\mathrm{bc}})_I$, system-level skewness $\mathrm{skw.}(g_I^{\mathrm{bc}})_I$, and small-world coefficient c_{swn}. Curves compare 300, 350, 400, and 450 K.

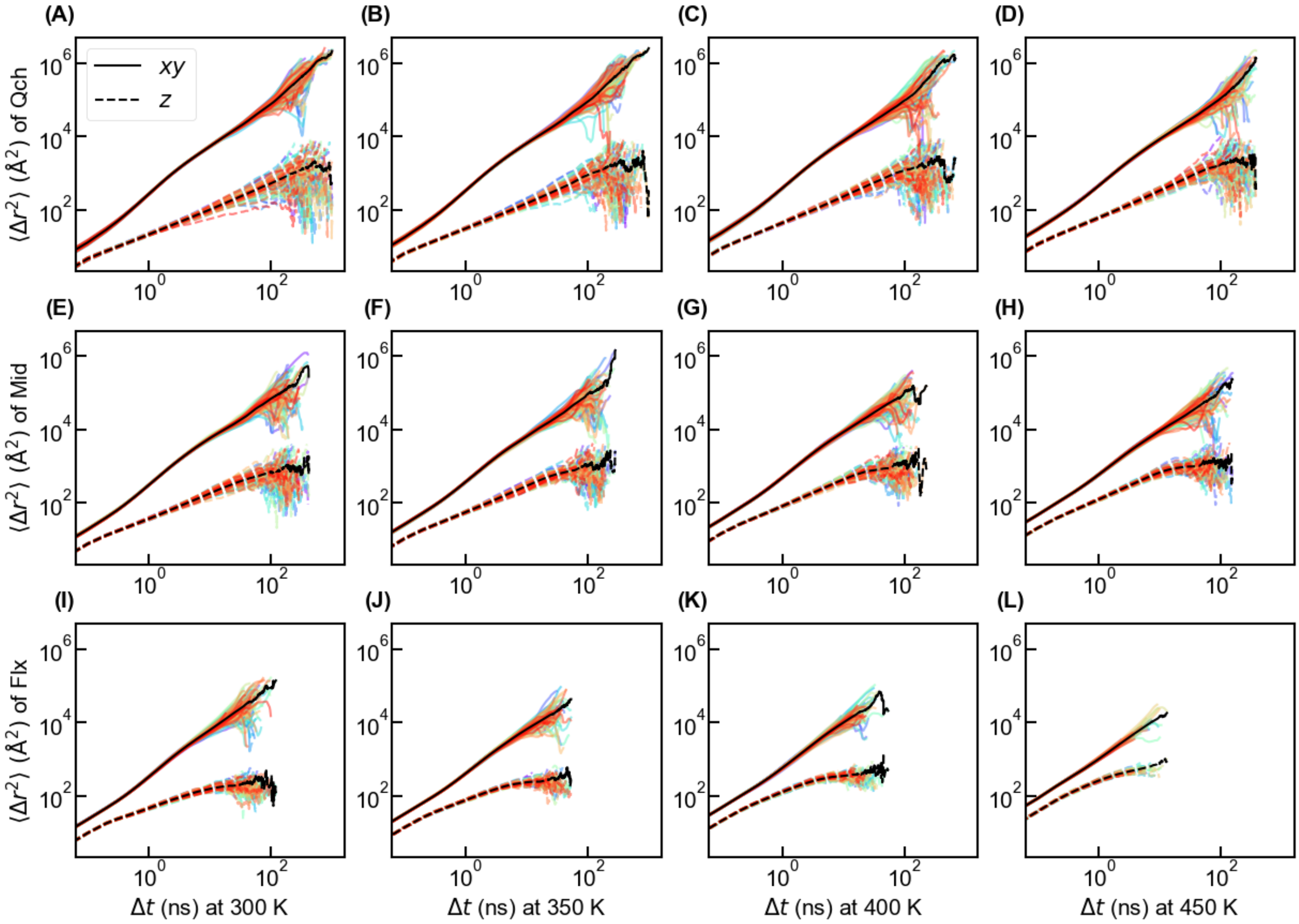

Figure S47: Chain-level mean-squared displacement (MSD) inside the slab. Rows correspond to Qch, Mid, and Flx; columns correspond to 300, 350, 400, and 450 K. Individual chain center-of-mass MSD curves are colored by chain index; solid curves show in-plane (xy) motion and dashed curves show slab-normal (z) motion. Black curves show chain-averaged MSD components.

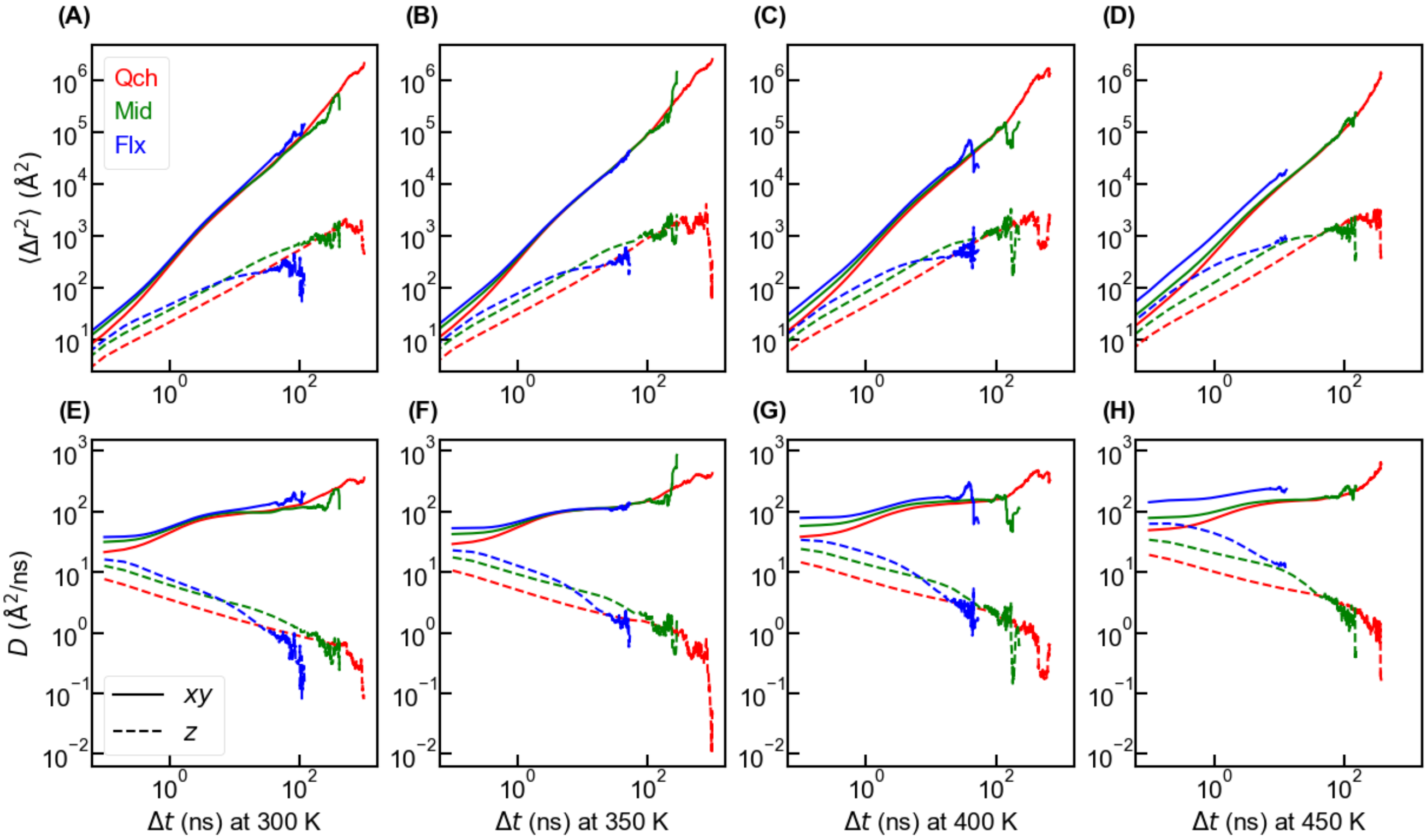

Figure S48: Model comparison of chain-level diffusion dynamics at fixed temperature. (A–D) Chain-averaged mean-squared displacement (MSD) inside the slab at 300, 350, 400, and 450 K. (E–H) Apparent diffusion coefficient from the same trajectories. Solid curves show in-plane (xy) motion and dashed curves show slab-normal (z) motion.

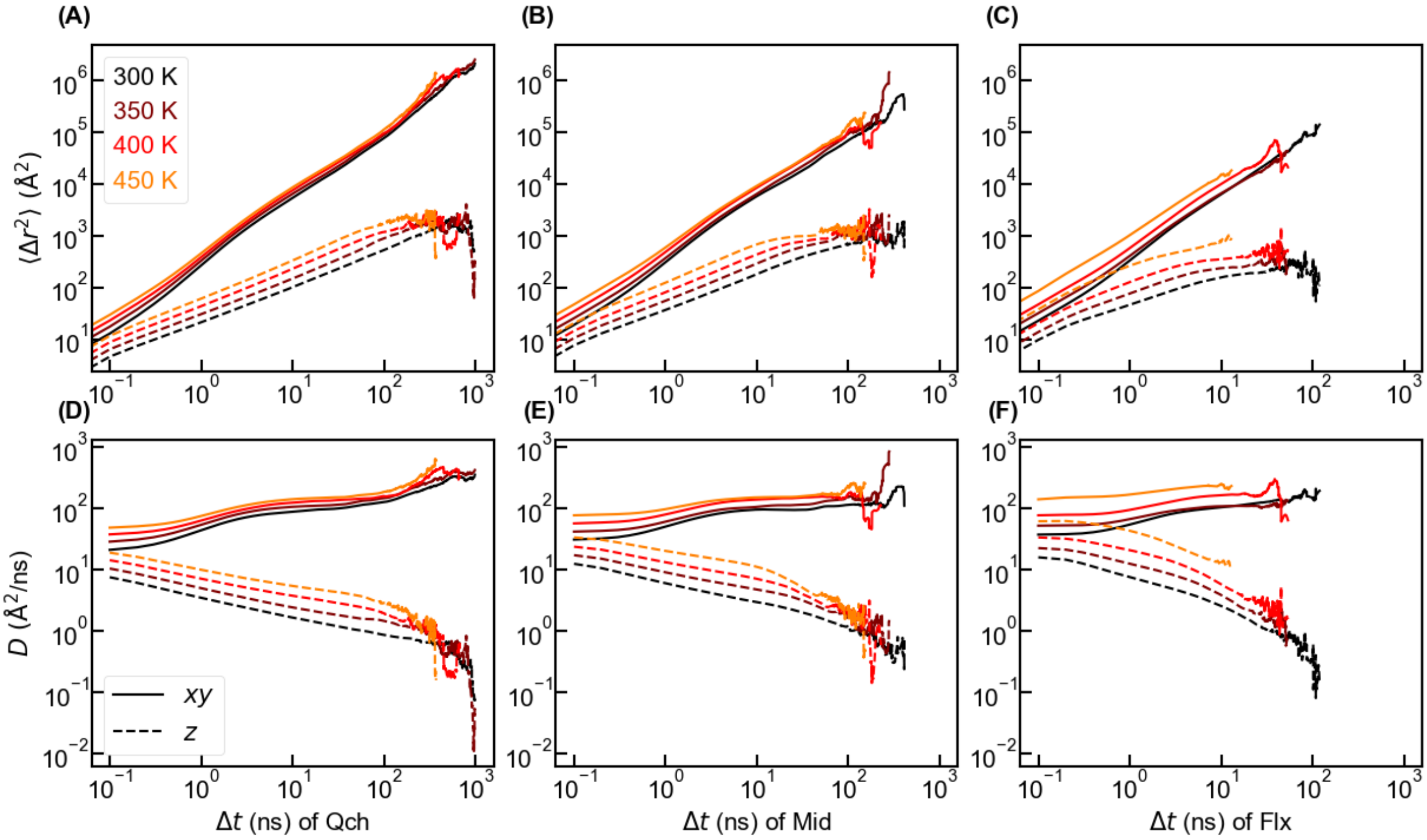

Figure S49: Temperature comparison of chain-level diffusion dynamics within each model. (A–C) Chain-averaged mean-squared displacement (MSD) inside the slab for Qch, Mid, and Flx. (D–F) Apparent diffusion coefficient from the same trajectories. Solid curves show in-plane (xy) motion and dashed curves show slab-normal (z) motion; colors denote temperature.

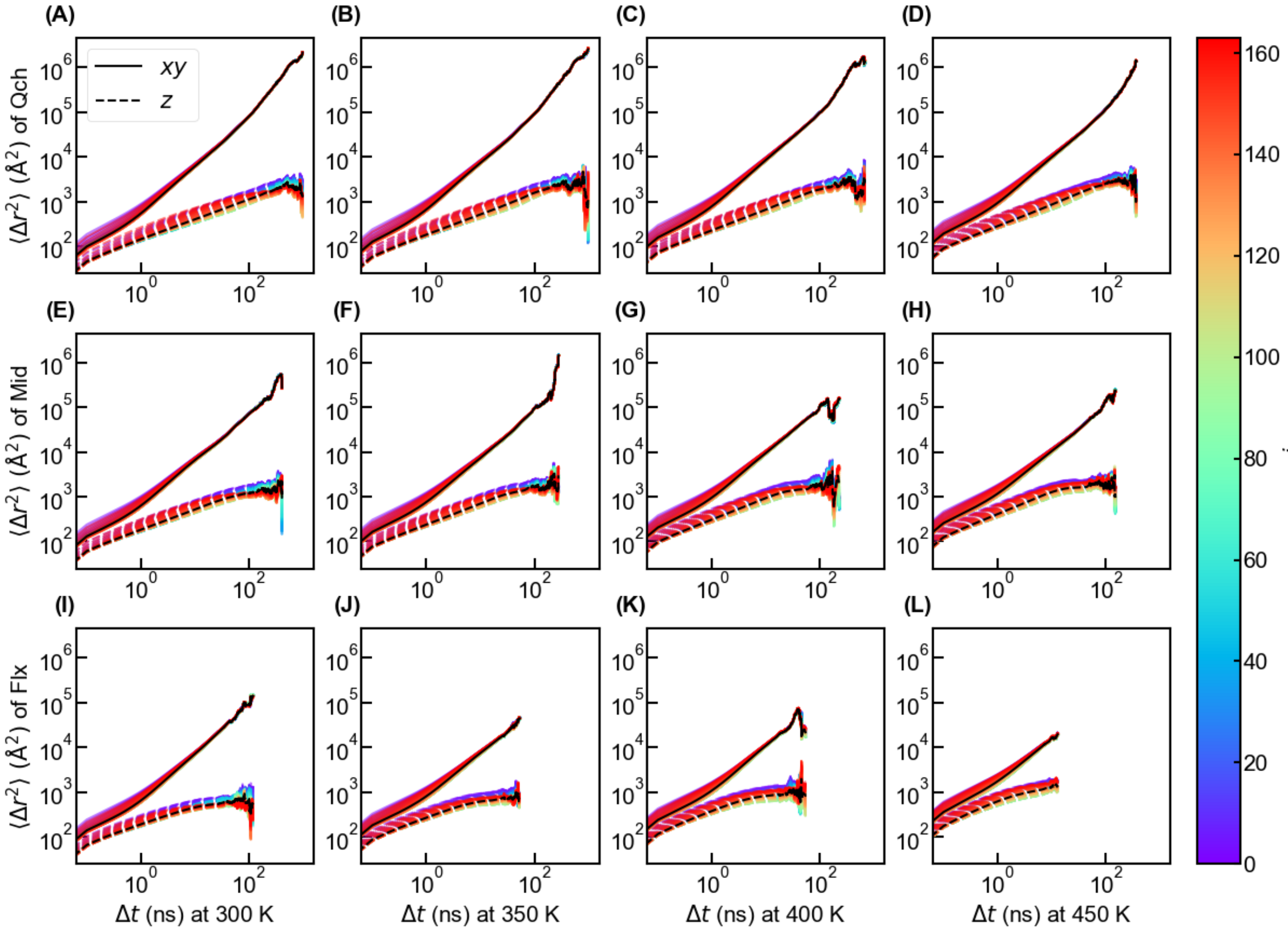

Figure S50: Residue-level mean-squared displacement (MSD) inside the slab. Rows correspond to Qch, Mid, and Flx; columns correspond to 300, 350, 400, and 450 K. Individual residue MSD curves are colored by residue index; solid curves show in-plane (xy) motion and dashed curves show slab-normal (z) motion. Black curves show residue-averaged MSD components.

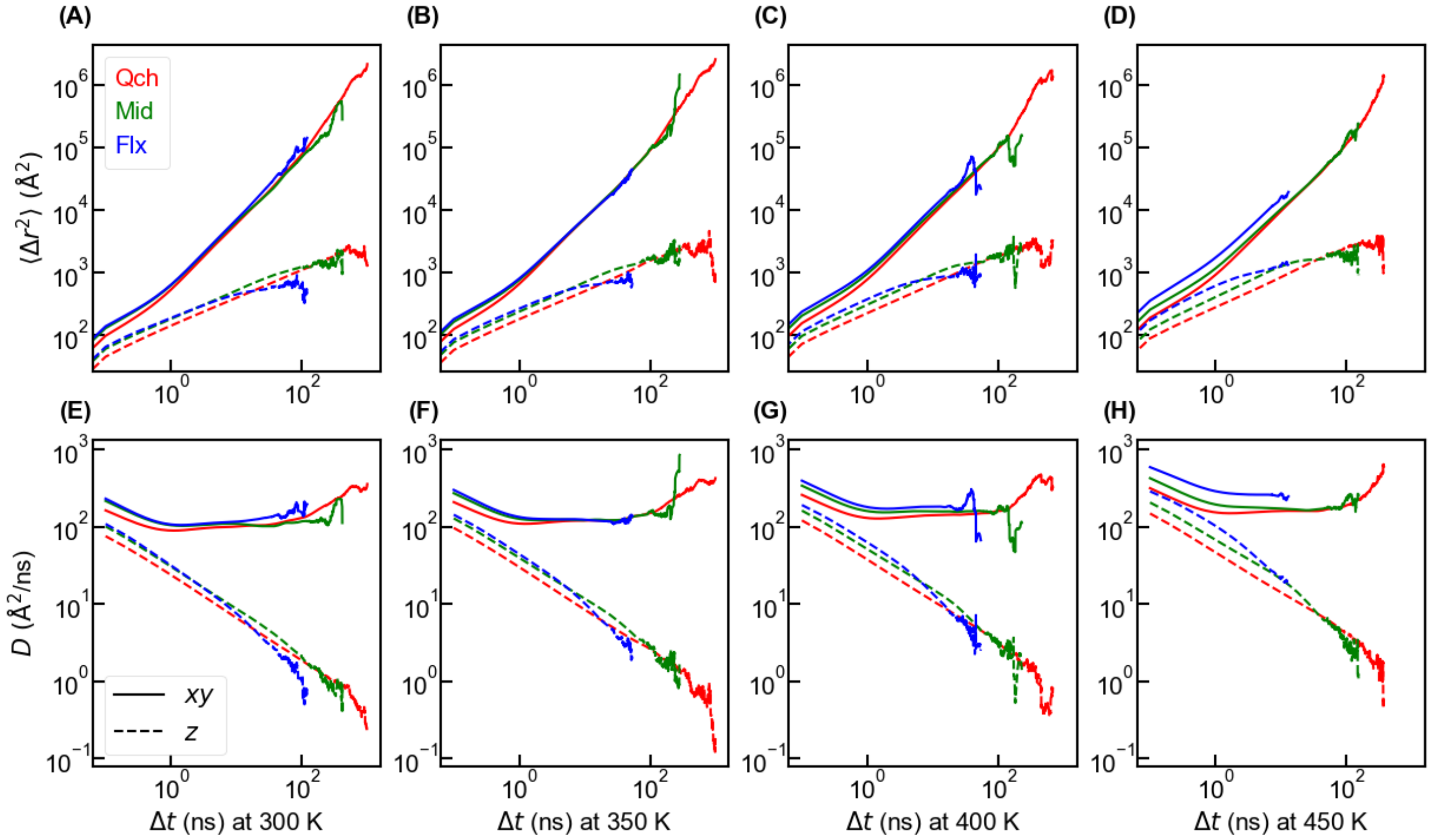

Figure S51: Model comparison of residue-level dynamics at fixed temperature. (A–D) Residue-averaged mean-squared displacement (MSD) inside the slab at 300, 350, 400, and 450 K. (E–H) Apparent diffusion coefficient from the same trajectories. Solid curves show in-plane (xy) motion and dashed curves show slab-normal (z) motion.

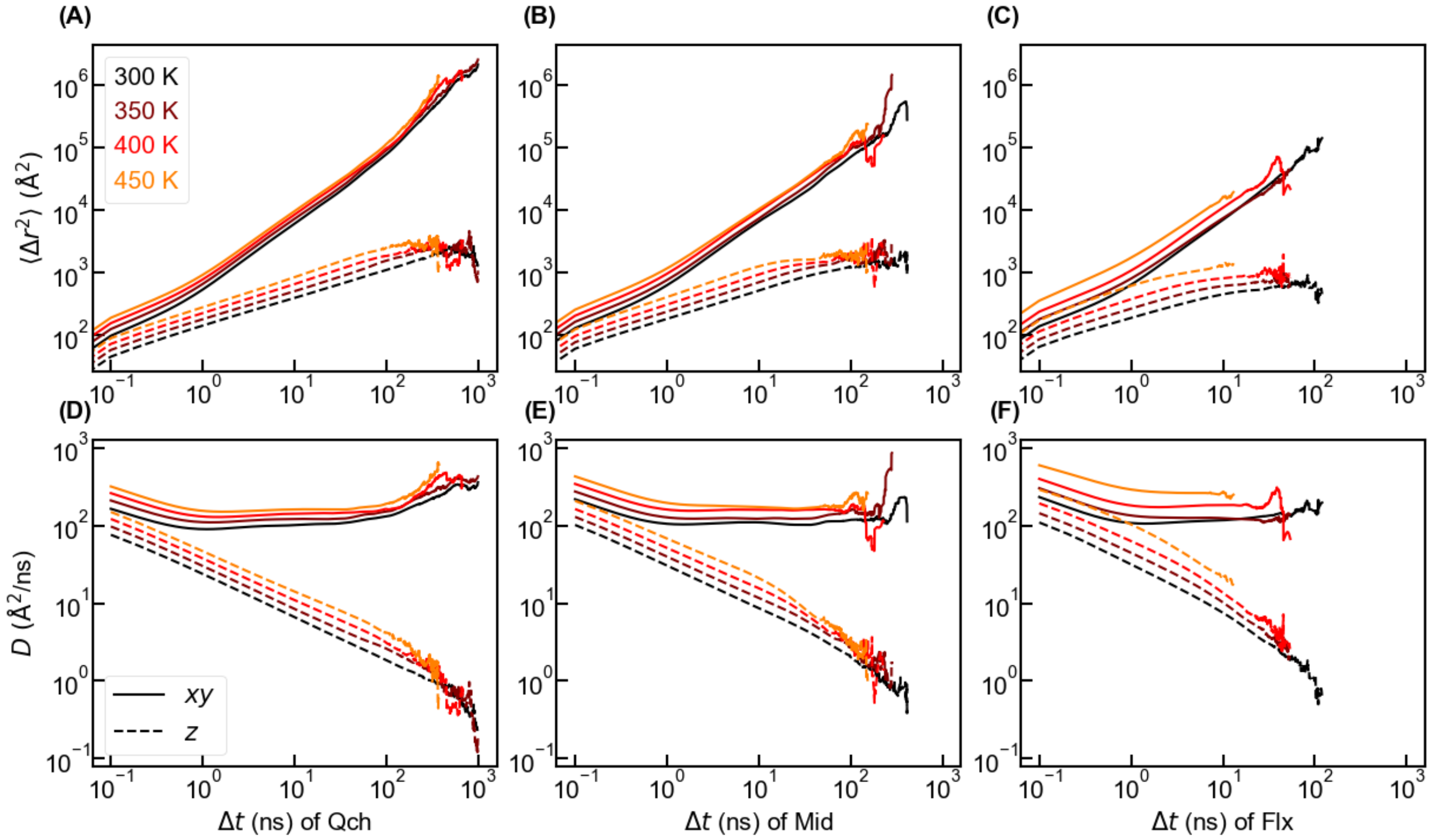

Figure S52: Temperature comparison of residue-level dynamics within each model. (A–C) Residue-averaged mean-squared displacement (MSD) inside the slab for Qch, Mid, and Flx. (D–F) Apparent diffusion coefficient from the same trajectories. Solid curves show in-plane (xy) motion and dashed curves show slab-normal (z) motion; colors denote temperature.

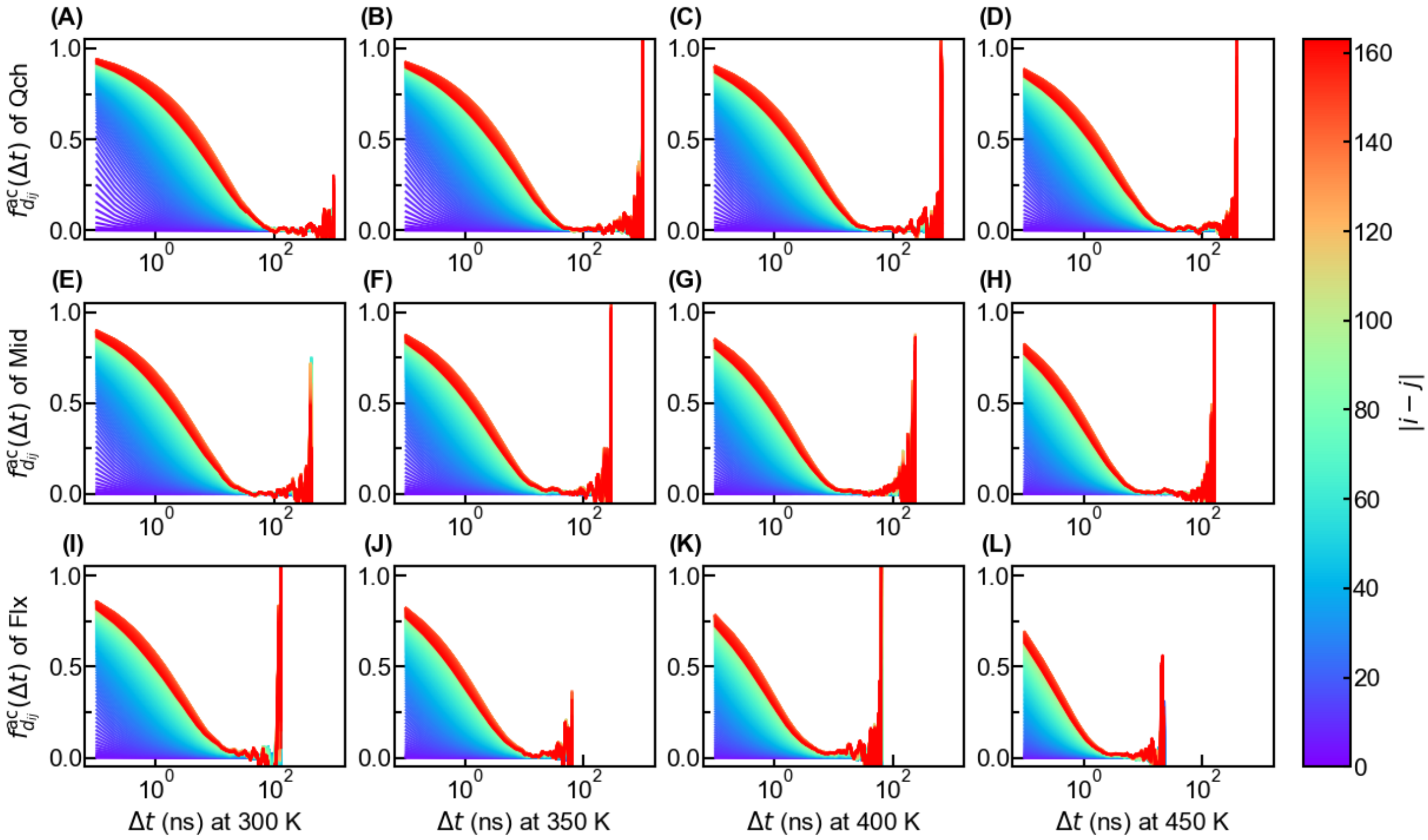

Figure S53: Autocorrelation of intrachain residue-pair distances inside the condensed slab. Rows correspond to Qch, Mid, and Flx; columns correspond to 300, 350, 400, and 450 K. Curves show the distance autocorrelation function $f^{\mathrm{ac}}_{d_{ij}}(\Delta t)$ averaged over residue pairs with the same sequence separation and colored by $|i-j|$.

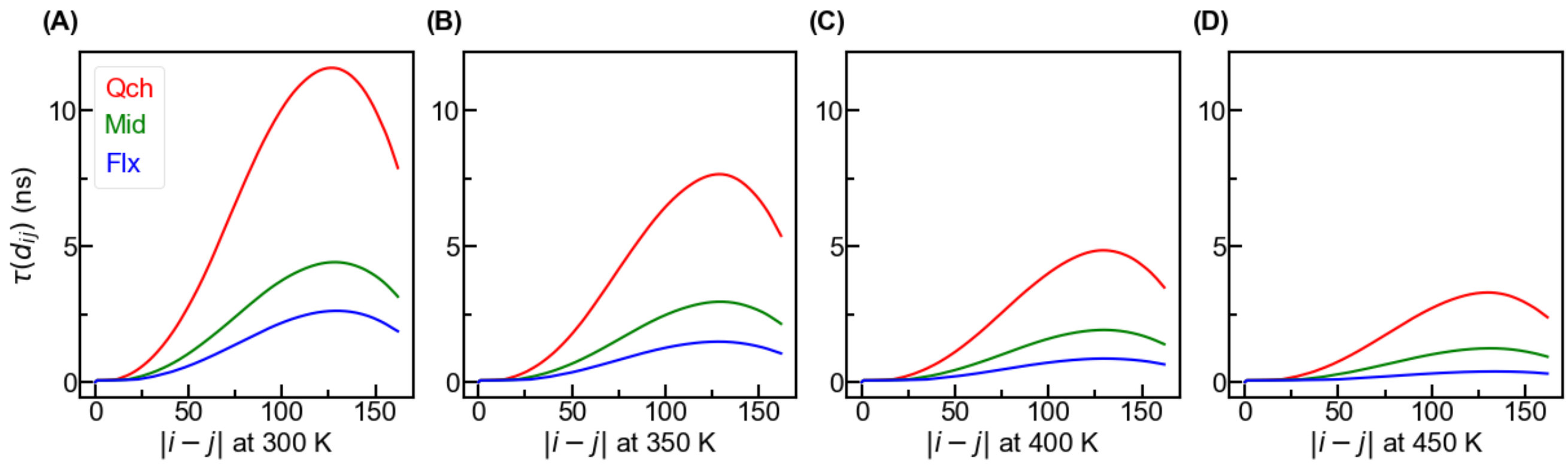

Figure S54: Model comparison of intrachain distance relaxation inside the condensate at fixed temperature. (A–D) Relaxation time $\tau(d_{ij})$ as a function of sequence separation $|i - j|$ at 300, 350, 400, and 450 K for Qch, Mid, and Flx.

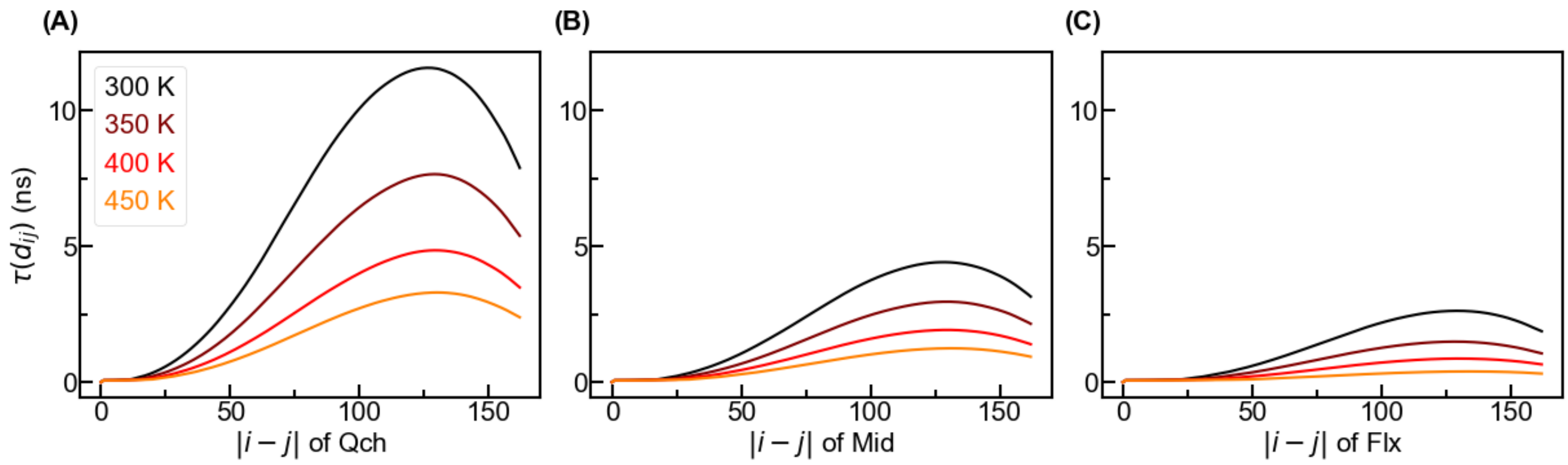

Figure S55: Temperature comparison of intrachain distance relaxation inside the condensate within each model. (A–C) Relaxation time $\tau(d_{ij})$ as a function of sequence separation $|i - j|$ for Qch, Mid, and Flx, with curves colored by temperature.

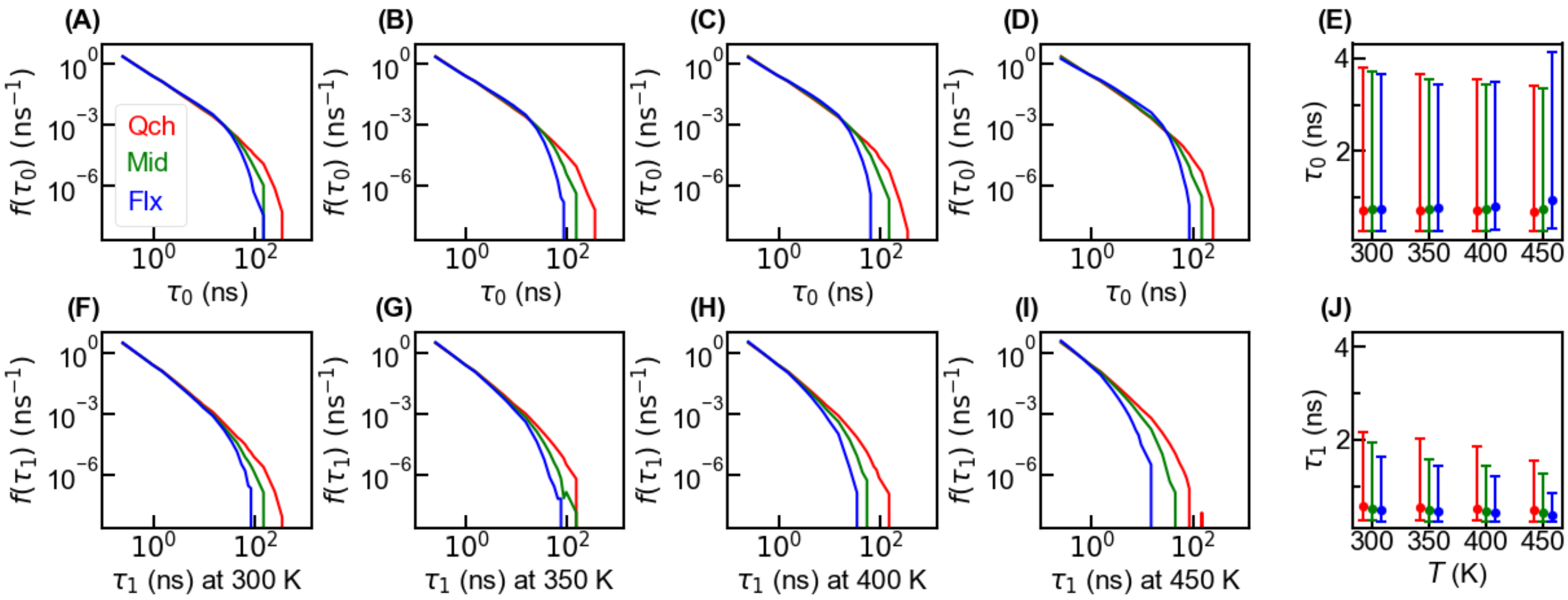

Figure S56: Model comparison of chain-pair contact-state lifetimes at fixed temperature. (A–D) Probability densities of non-contact lifetimes τ_0 at 300, 350, 400, and 450 K. (F–I) Probability densities of contact lifetimes τ_1 at the same temperatures. (E,J) Mean non-contact and contact lifetimes grouped by temperature for Qch, Mid, and Flx. Error bars denote asymmetric log-space dispersion.

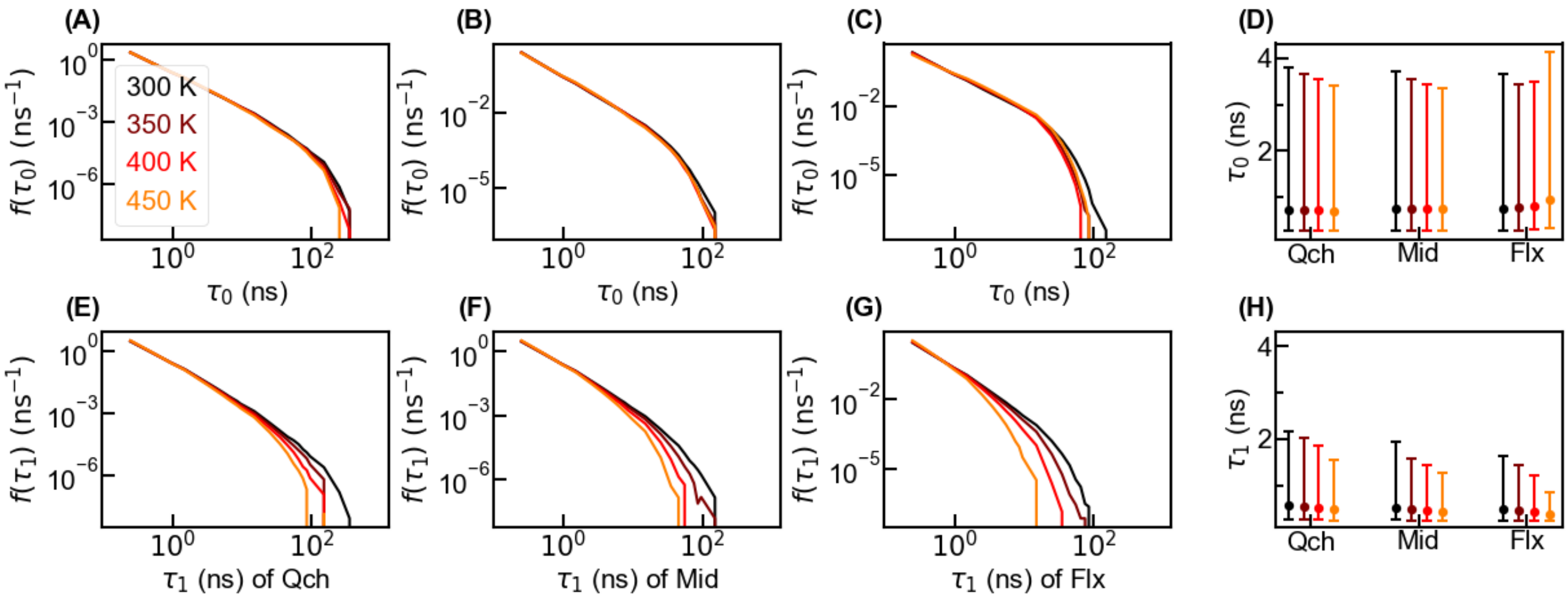

Figure S57: Temperature comparison of chain-pair contact-state lifetimes within each model. (A–C) Probability densities of non-contact lifetimes τ_0 for Qch, Mid, and Flx. (E–G) Probability densities of contact lifetimes τ_1 for the same models. (D,H) Mean non-contact and contact lifetimes grouped by model, with curves and symbols colored by temperature. Error bars denote asymmetric log-space dispersion.

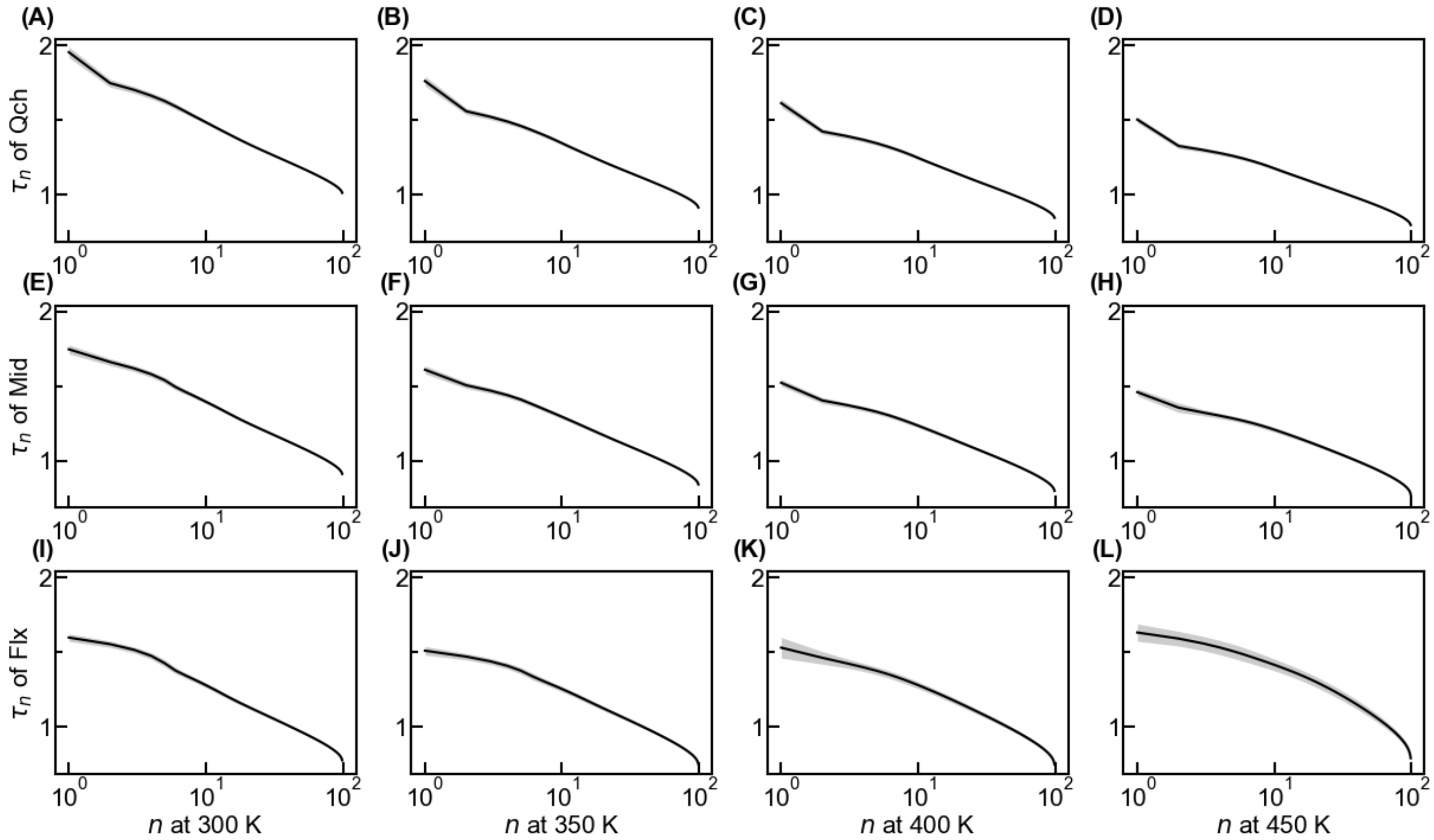

Figure S58: Mode-resolved relaxation times from network-mode analysis. Rows correspond to Qch, Mid, and Flx; columns correspond to 300, 350, 400, and 450 K. Black curves show the mean mode relaxation time τ_n as a function of mode index n, and gray bands show one standard deviation.

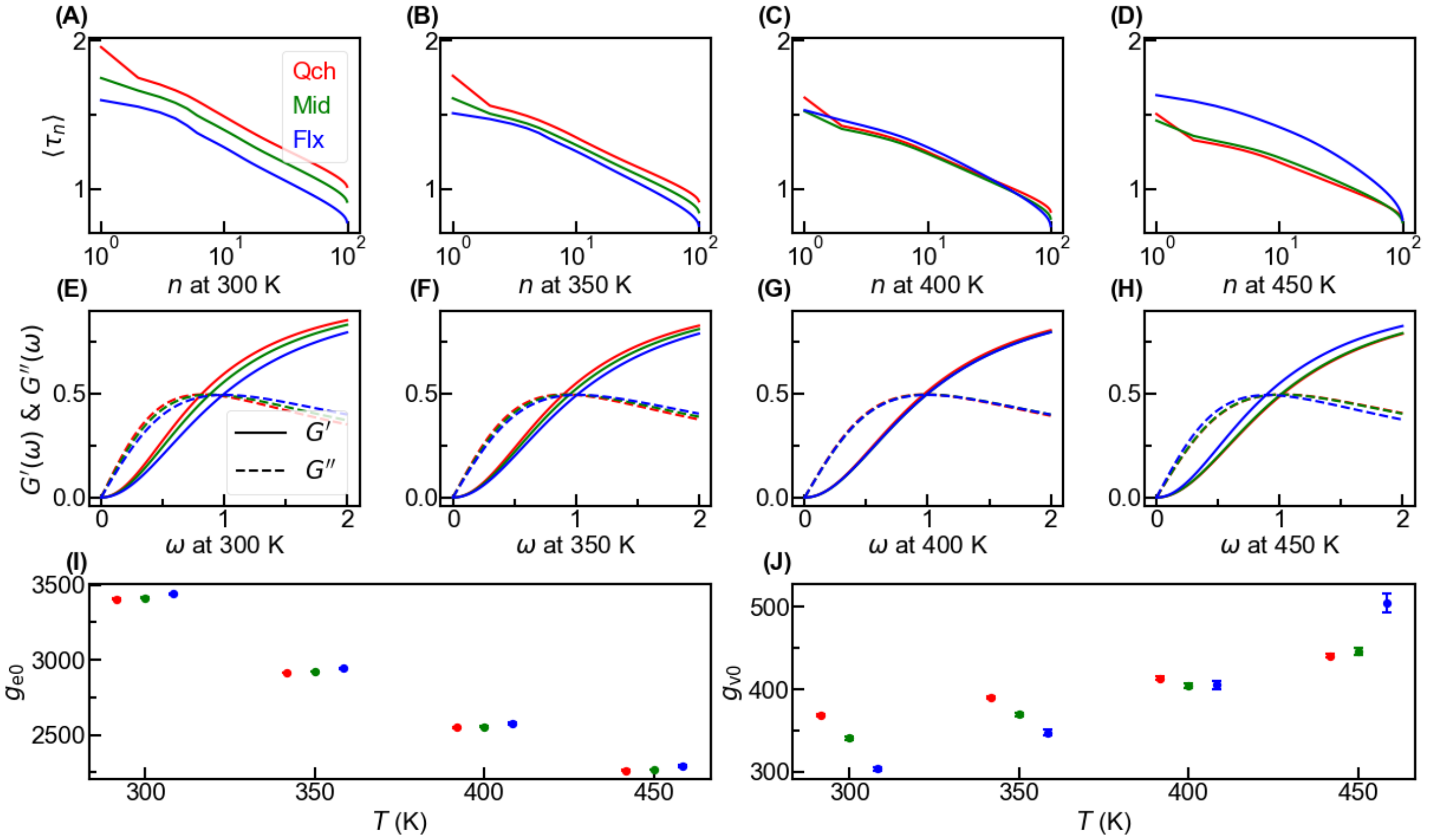

Figure S59: Model comparison of viscoelastic spectra at fixed temperature. Columns in the first two rows correspond to 300, 350, 400, and 450 K. Row 1 shows mean mode relaxation time $\langle \tau_n \rangle$ versus mode index n. Row 2 shows storage and loss moduli, $G'(\omega)$ and $G''(\omega)$. Row 3 summarizes elastic and viscous prefactors, g_{e0} and g_{v0}, as functions of temperature.

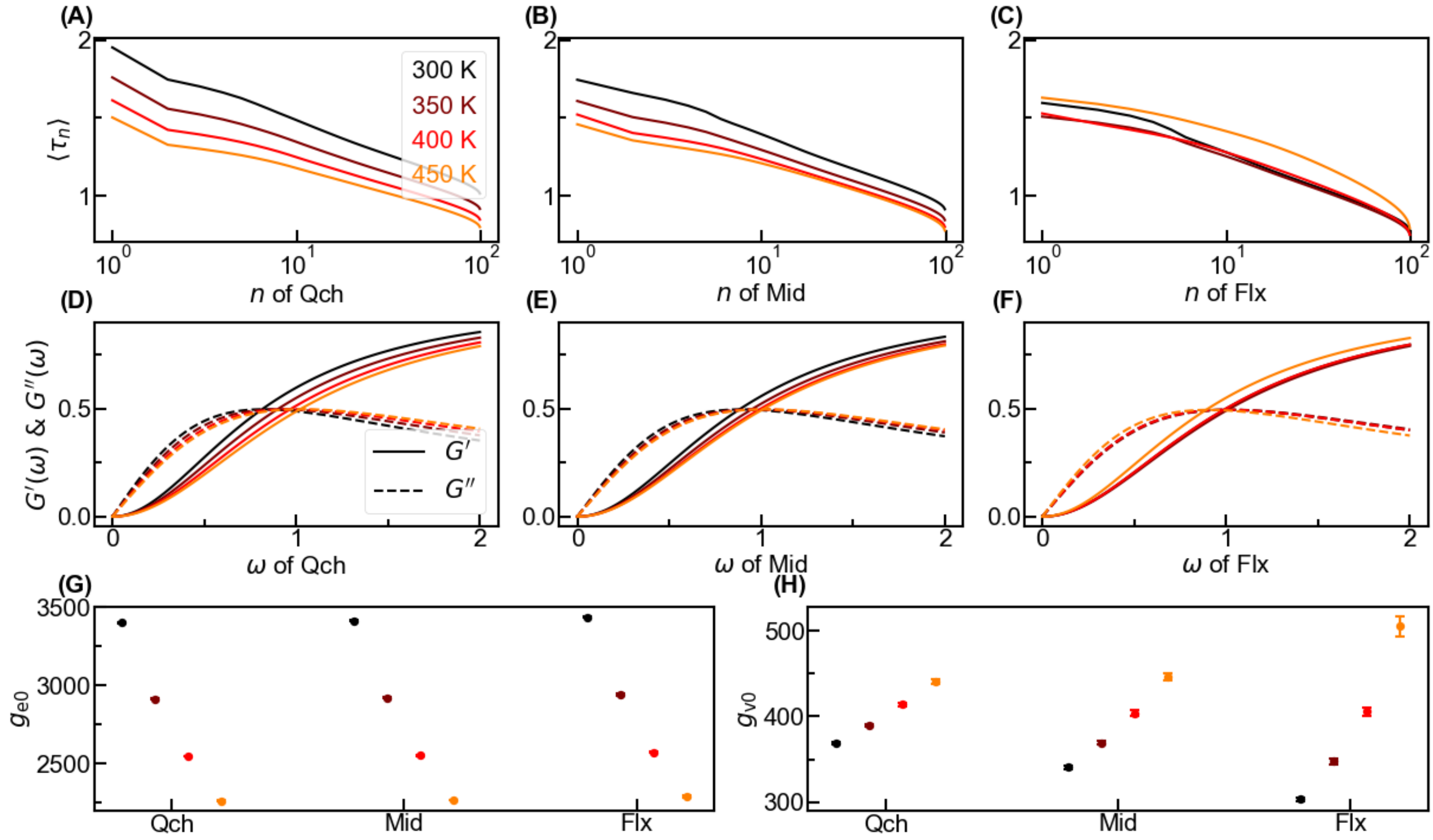

Figure S60: Temperature comparison of viscoelastic spectra within each model. Columns in the first two rows correspond to Qch, Mid, and Flx. Row 1 shows mean mode relaxation time $\langle \tau_n \rangle$ versus mode index n. Row 2 shows storage and loss moduli, $G'(\omega)$ and $G''(\omega)$. Row 3 summarizes elastic and viscous prefactors, g_{e0} and g_{v0}, grouped by model with symbols colored by temperature.

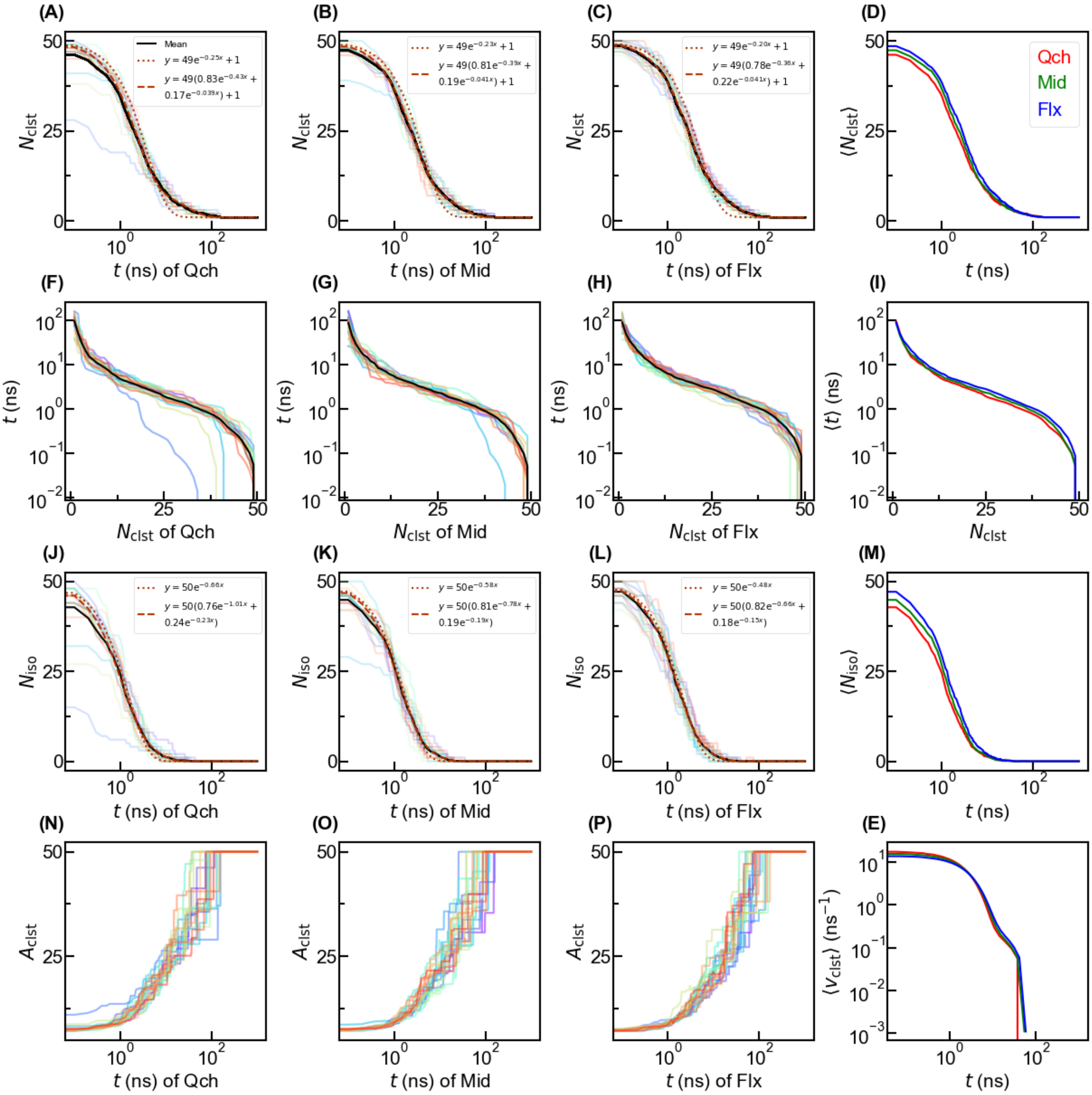

Figure S61: Detailed analysis of 50-chain condensation kinetics. Row 1 shows the number of clusters $N_{\mathrm{clst}}(t)$ for individual repeats, exponential fits, and the model-averaged comparison. Row 2 shows the required time t_{req} to reach each cluster number. Row 3 shows the number of isolated chains $N_{\mathrm{iso}}(t)$ and exponential fits. Row 4 shows the cluster-size amplitude A_{clst} and fitted cluster-growth velocity $\langle v_{\mathrm{clst}} \rangle$ for individual repeats. Columns 1–3 correspond to Qch, Mid, and Flx; column 4 compares the model averages.

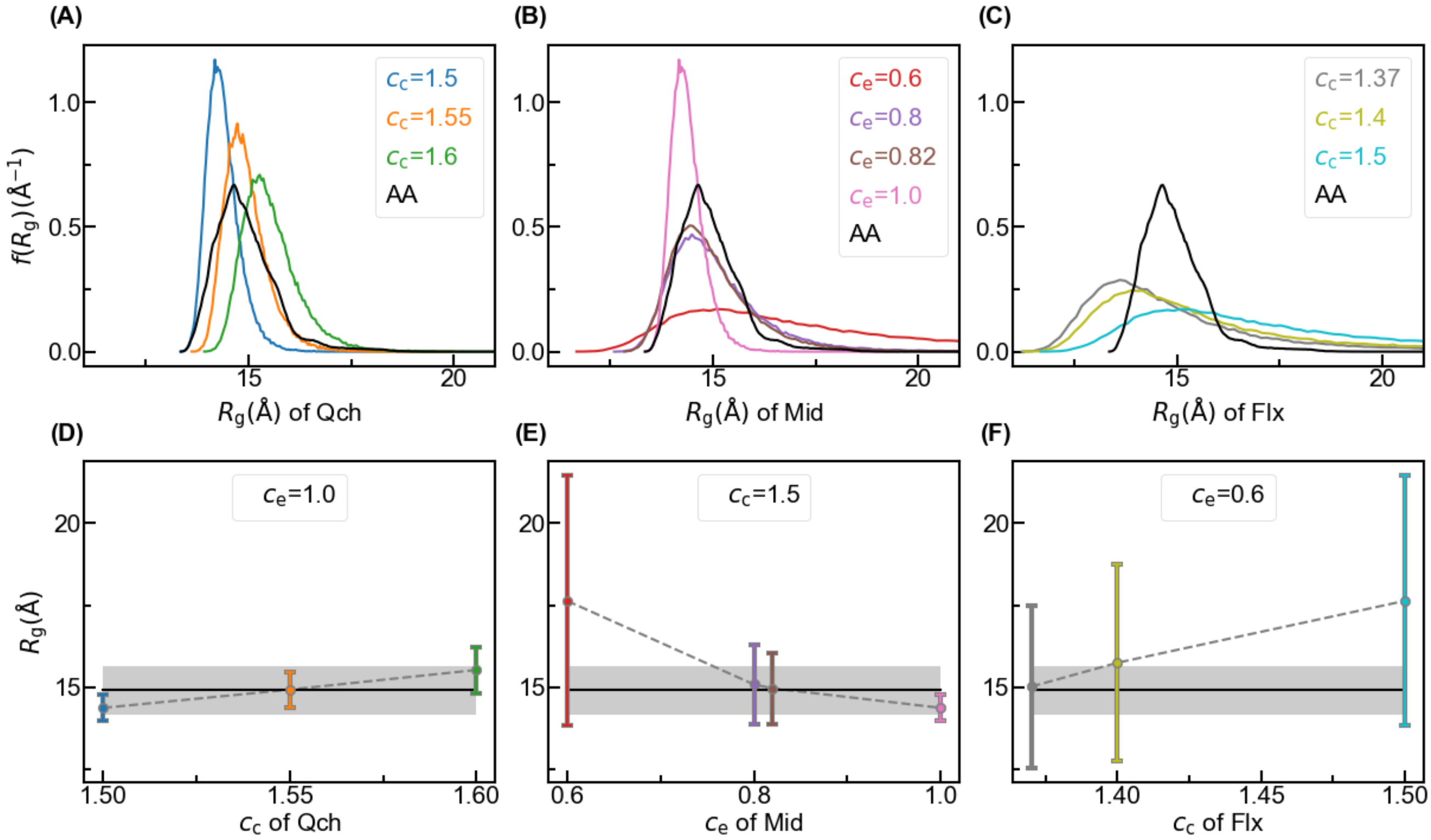

Figure S62: Calibration of isolated-chain radius of gyration (R_g) distributions during coarse-grained parameter selection. (A–C) R_g probability distributions for Qch, Mid, and Flx parameter scans compared with the all-atom (AA) reference. The varied parameter is the contact-range coefficient c_c for Qch and Flx and the excluded-volume scale coefficient c_e for Mid; the fixed companion parameter is indicated in each panel. The set with fixed $c_e = 1.0$ corresponds to the original excluded-volume potential. The set with fixed $c_c = 1.5$ follows a commonly used contact criterion for IDPs [*1*]. These two scans generate Qch and Mid. To generate the high-fluctuation Flx model, $c_e = 0.6$ is used. With one parameter fixed, scanning the other parameter allows the simulated $\langle R_g \rangle$ to match the AA reference. (D–F) Mean and standard deviation of R_g across the same parameter scans; the black line and gray band mark the AA mean and AA mean ± standard deviation.

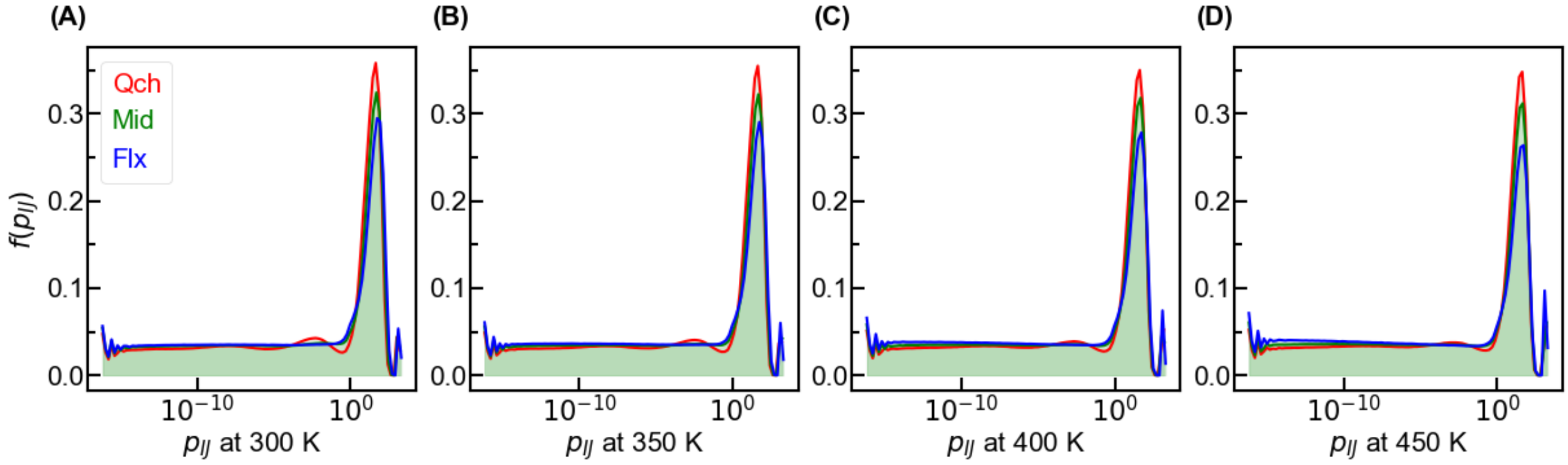

Figure S63: Model comparison of chain-pair contact-sum distributions at fixed temperature. (A–D) Probability density $f(p_{IJ})$ of the chain-pair contact sum p_{IJ} at 300, 350, 400, and 450 K for Qch, Mid, and Flx.

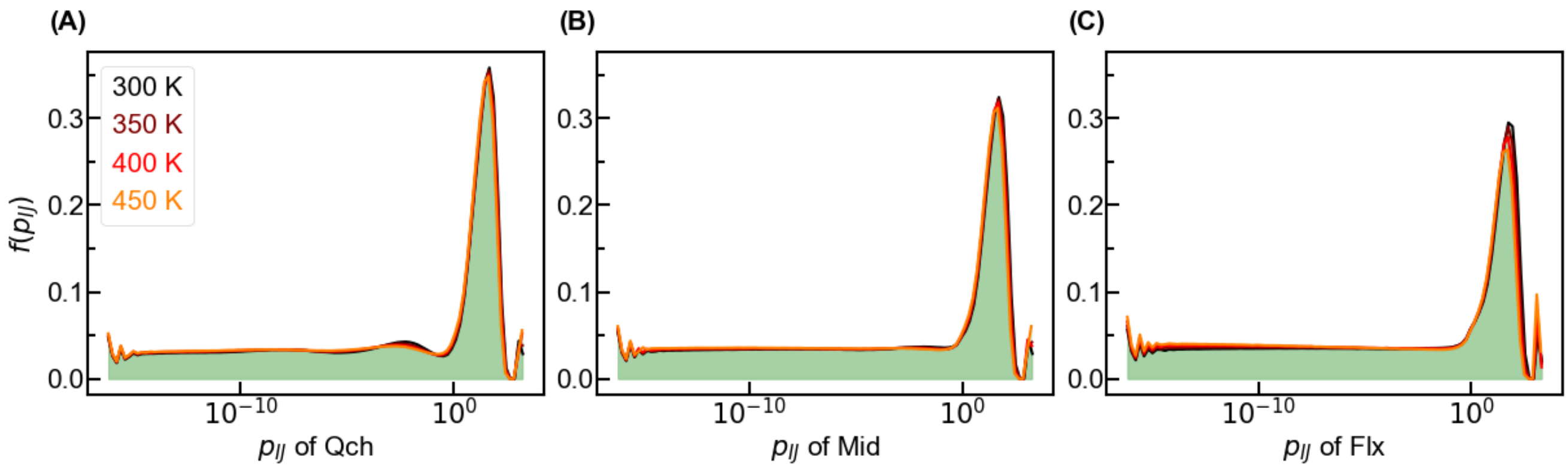

Figure S64: Temperature comparison of chain-pair contact-sum distributions within each model. (A–C) Probability density $f(p_{IJ})$ of the chain-pair contact sum p_{IJ} for Qch, Mid, and Flx, with curves colored by temperature.

References

[1] Mohanty, P.; Shenoy, J.; Rizuan, A.; Mercado-Ortiz, J. F.; Fawzi, N. L.; Mittal, J. A synergy between site-specific and transient interactions drives the phase separation of a disordered, low-complexity domain. *Proc. Natl. Acad. Sci. U.S.A.* **2023**, *120*, e2305625120.
